# Multicomponent synthesis of novel imidazole-pyran derivatives: *in silico* and *in vitro* studies

**DOI:** 10.1039/d5ra05264e

**Published:** 2026-01-02

**Authors:** Zahra Jamshidi, Seyed Mohammad Taghdisi, Khalil Abnous, Razieh Ghodsi, Farzin Hadizadeh

**Affiliations:** a Department of Medicinal Chemistry, School of Pharmacy, Mashhad University of Medical Sciences Mashhad Iran hadizadehf@mums.ac.ir +98 51 38823251 +98 51 31801128; b Student Research Committee, Mashhad University of Medical Sciences Mashhad Iran; c Targeted Drug Delivery Research Center, Pharmaceutical Technology Institute, Mashhad University of Medical Sciences Mashhad Iran; d Department of Pharmaceutical Biotechnology, School of Pharmacy, Mashhad University of Medical Sciences Mashhad Iran; e Pharmaceutical Research Center, Pharmaceutical Technology Institute, Mashhad University of Medical Sciences Mashhad Iran; f Biotechnology Research Center, Pharmaceutical Technology Institute, Mashhad University of Medical Sciences Mashhad Iran

## Abstract

Herein, a three-component reaction was used for the synthesis of imidazole-pyran derivatives (4a–n) through the reaction between imidazol-5-carbaldehyde (1a–n), malononitrile (2), and methyl acetoacetate (3). The reaction process was simple, quick, proceeded without the need for any purification technique and used green solvents. The synthesized substances (4a–n) were evaluated for their potential anticancer effects on the MCF-7 (breast cancer), HT29 (colon cancer), and A2780cis (cisplatin-resistant ovarian cancer) cell lines, and a control normal cell line, CHO (Chinese hamster ovary). Notably, compounds 4e and 4h demonstrated pronounced effects on the MCF-7 cell line, with an IC_50_ value of 11.74 ± 0.17 µM and 9.44 ± 0.17 µM, respectively. Compounds 4e and 4h also showed appropriate toxicity in the HT-29 and A2780cis cell lines. These two compounds (4e and 4h) also demonstrated the ability to suppress colony formation and trigger apoptosis in MCF-7 cells. Additionally, *in silico* studies, such as molecular docking and molecular dynamics, were conducted on VEGFR2. This approach investigated the interaction and binding types of the synthesized compounds in the receptor, their stability, and the change in the protein structure during molecular docking and molecular dynamics.

## Abstract 2

New imidazole-pyran derivatives were designed, synthesized, and evaluated for their biological activity. 4e and 4h demonstrated cytotoxic activity on MCF-7 cells. *In silico* studies displayed their good binding at the active site of VEGFR2.

## Graphical abstract

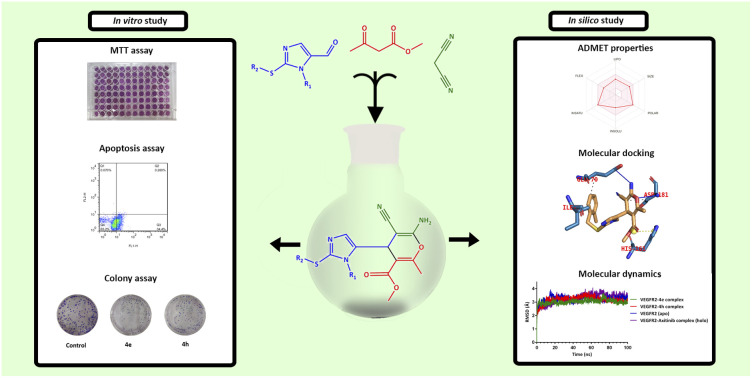

## 1. Introduction

Cancer can spread to nearby or distant organs, which makes it a serious threat to life.^1^ Sufficient nutrients and oxygen are supplied by the expanded new vascular network, which also facilitates the removal of waste products from cancer cells.^2^ The process of generating new blood vessels, originating from the pre-existing vascular network, is referred to as angiogenesis. Some angiogenic activators have been identified, for example, angiogenin 3, vascular endothelial growth factor (VEGF),^4^ transforming growth factor (TGF)-α,^5^ and platelet-derived endothelial growth factor.^6^ One of the important neoplastic vascularizations is the VEGF family and their receptors (VEGFR). In the cancerous tissues and near stroma, the VEGF family is released under the effect of certain cytokines and different growth factors.^7^ Placental growth factor (PGF), VEGF-A, VEGF-B, VEGF-C and VEGF-D are members of the human VEGF family. VEGFR1 (Flt-1), VEGFR3 (Flt-4), and VEGFR2 (KDR) are three main VEGF receptors, and two non-protein kinase co-receptors include Neuropilin-1 (NRP1) and Neuropilin-2 (NRP2).^8,9^ The role of VEGFR2 in angiogenesis is well established, and more comprehensive reviews on the role of VEGFR2 in angiogenesis have been recently published.^10–12^

Imidazoles exhibit a wide spectrum of biological effects, including, anticancer,^13^ anti-depressant,^14^ antiviral,^15^ anti-tubercular,^16^ anti-inflammatory,^17^ anti-fungal,^18^ and antimicrobial^19^ properties. In 2023, Mannich-based imidazole derivatives were also reported to possess larvicidal, antibacterial, and antifungal activities.^20^ Well-known anti-cancer drugs contain imidazole units, such as zoledronic acid, tipifarnib, axitinib, dacarbazine, and azathioprine.^13^ The pyran scaffold exists in various natural products, such as xanthones, coumarins, and flavonoids, exists.^21^

Some compounds containing a pyran unit show anticancer activity with an IC_50_ in the micromolar range. For example, the antitumor activity of novel imidazole–pyridine hybrid molecules against liver (HepG2, PLC/PRF/5, and HUH-7), lung (H1299), and colon (HCT116) tumor cell lines was previously investigated.^22^

One of the strong strategies in synthetic organic chemistry is multicomponent reactions (MCRs). In MCRs, at least three reagents are mixed to produce a new product in one step. MCRs usually save energy and time and also offer appropriate yields.^23–25^ It seems valuable to synthesize and characterize certain imidazoles fused with other heterocyclic rings and evaluate their properties. A novel compound of tetrasubstituted imidazole bearing pyrimidine sulfonamide pharmacophores was rationalized, synthesized, and screened for its anticancer properties. These compounds demonstrated efficacy against HER2 and EGFR (two mutants L858R and T790M, respectively).^26^

According to the biological importance of imidazole compounds and in follow-up our research programs, a number of novel imidazole-pyran derivatives was designed, synthesized, and investigated as VEGFR2 kinase inhibitors. To investigate the potential of synthetic compounds as drug candidates, *in vitro* and *in silico* studies were performed. *In vitro* studies, including MTT assay, colony assay, and apoptosis assay, were performed. *In silico* studies, including ADMET properties, target prediction, molecular docking, and molecular dynamics simulation, were also conducted.

## 2. Results and discussion

### 2.1. Chemistry

**Fig. 1:**
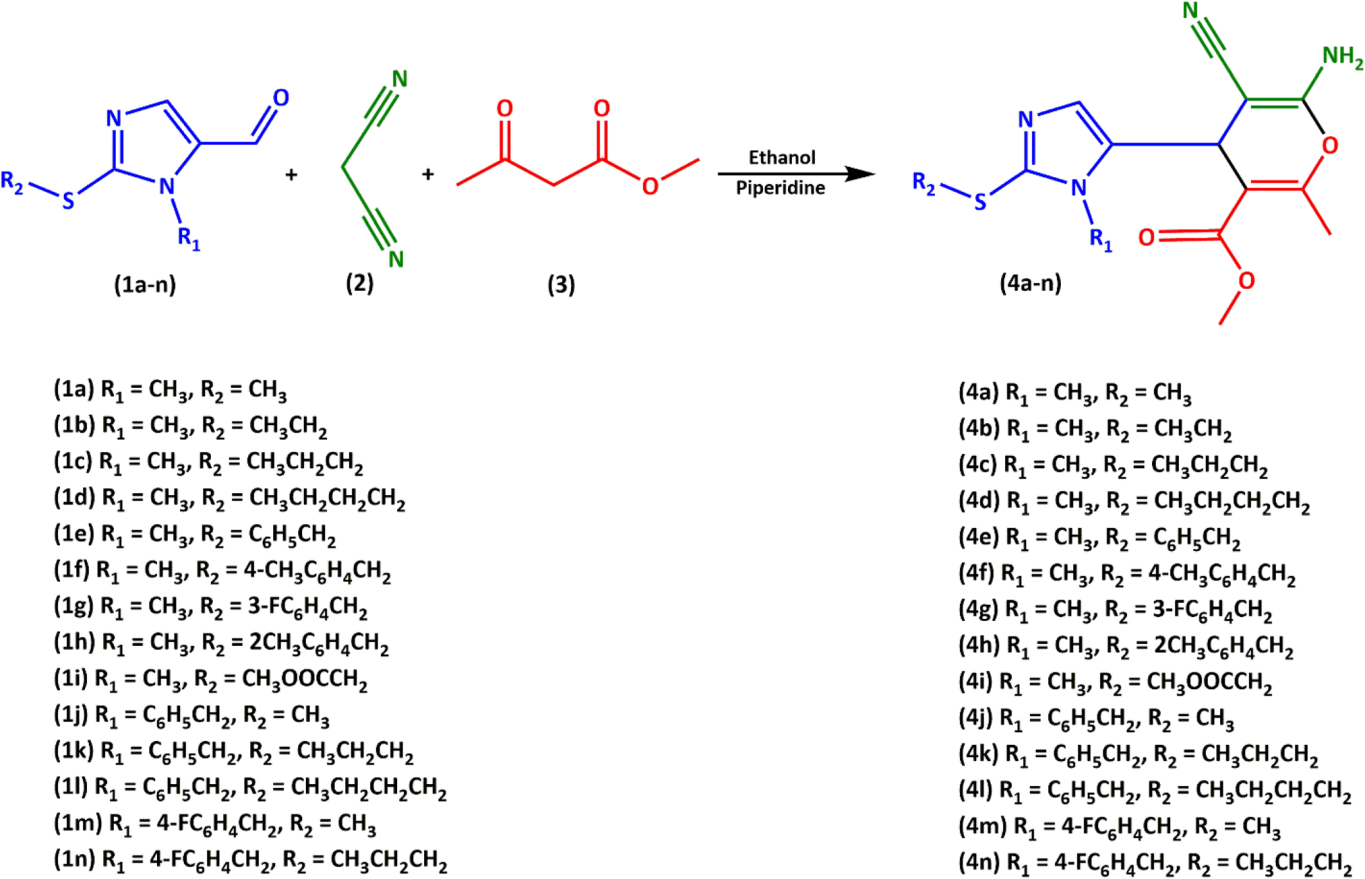
Reaction of imidazol-5-carbaldehyde (1a–n), malononitrile (2), and methyl acetoacetate (3).

Imidazole-5-carbaldehydes (1a–n) were synthesized following a previously developed method.^27^ We investigated the three-component reaction of imidazol-5-carbaldehydes (1a–n), malononitrile (2), methyl acetoacetate (3) under various conditions including basic, acidic, and metal catalysts in different solvents (Table 1). To optimize the reaction conditions, the three-component reaction was investigated in water, ethanol, methanol (polar/protic solvents), acetonitrile (non-protic solvent), and chloroform (non-polar solvent). Unfortunately, under catalyst-free conditions (Table 1, entry 6), no product was observed in the three-component reaction. Also, the reaction was carried out at temperatures ranging from reflux to room temperature (RT) in ethanol solvent, and we observed that the product yield decreased as the temperature increased (Table 1, entry 3). Finally, the best reaction conditions of piperidine catalyst, ethanol solvent, and room temperature were chosen (Table 1, entry 2) (Fig. 1). ^13^C NMR, ^1^H NMR, elemental analysis, IR, and MS spectroscopy (Fig. S1–S56) were used for the characterization of the chemical structures of the compounds (4a–n).

**Table 1:** Optimization of the reaction conditions

Entry	Solvent	Catalyst	Temp./°C	Time	Yield%
1	H_2_O	Piperidine	RT	24 h	—
2	Ethanol	Piperidine	RT	5 min	57
3	Ethanol	Piperidine	Reflux	5 min	Trace
4	Ethanol	CuCl_2_	RT	24 h	Trace
5	Ethanol	H_2_SO_4_	RT	24 h	—
6	Ethanol	—	RT	24 h	—
7	Methanol	Piperidine	RT	5 min	55
8	Chloroform	Piperidine	RT	5 min	20
9	Acetonitrile	Piperidine	RT	5 min	17

**Fig. 2:**
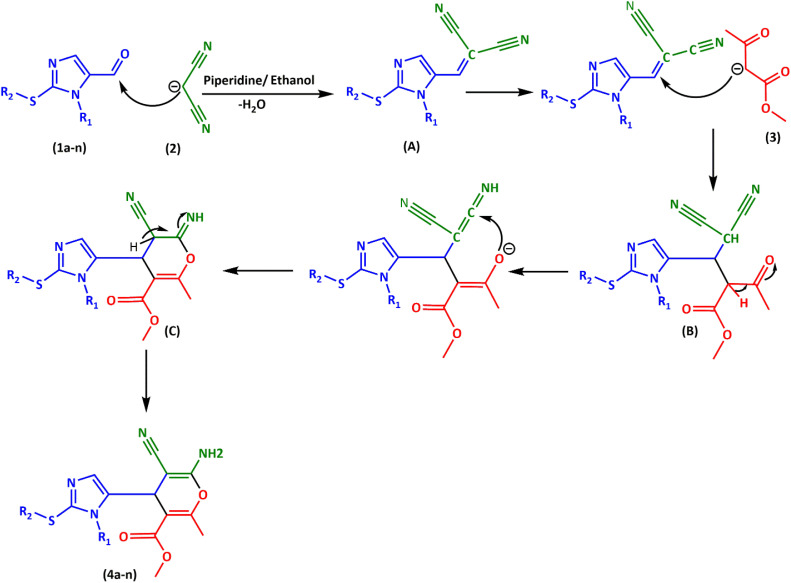
Proposed mechanism for the synthesis of imidazole-pyran 4a–n.

As shown in Fig. 2, based on the produced compounds (4a–n) of the three-component reaction, a possible mechanism was suggested. We think that initially, intermediate A is generated by Knoevenagel condensation between malononitrile (2) and imidazol-5-carbaldehydes (1a–n). Next, methyl acetoacetate (3) and intermediate A generated intermediate B by Michael addition. Under the influence of intramolecular cyclization, intermediate B was converted to intermediate C. Finally, tautomerization of intermediate C gave products 4a–n.

### 2.2. *In silico* studies

#### 2.2.1 *In silico* prediction of ADMET properties, physicochemical parameters, and target prediction

Absorption/human intestinal absorption (probability), distribution/fraction unbound/human (predictions), metabolism/CYP 2C19_substrate (predictions), excretion/drug half-life (probability), and toxicity/carcinogenesis (probability) of all the synthetic compounds (4a–n) were measured using Deep-PK (https://biosig.lab.uq.edu.au/deeppk/prediction). The predictions and probability of the ADMET properties are shown in Table 2. The synthesized compounds showed great absorption probability in the human intestine, ranging from 0.875 to 0.971. All the synthetic compounds (4a–n) were not substrates of CYP2C19. Consequently, we did not expect drug interaction upon their administration.

**Table 2:** *In silico* ADMET prediction of the derivatives (4a–n) using Deep-PK and SwissADME

Compound	Absorption/human intestinal absorption (probability)	Distribution/fraction unbound/human (predictions)	Metabolism/CYP 2C19_substrate (predictions)	Excretion/half-life of drug (probability)	PAINS (alert)
4a	0.918	0.63	Non-substrate	0.47	0
4b	0.946	0.57	Non-substrate	0.419	0
4c	0.936	0.61	Non-substrate	0.414	0
4d	0.917	0.67	Non-substrate	0.401	0
4e	0.929	1.25	Non-substrate	0.467	0
4f	0.933	1.31	Non-substrate	0.419	0
4g	0.95	1.25	Non-substrate	0.493	0
4h	0.924	1.24	Non-substrate	0.379	0
4i	0.875	0.58	Non-substrate	0.419	0
4j	0.947	1.11	Non-substrate	0.381	0
4k	0.958	1.14	Non-substrate	0.323	0
4l	0.941	1.22	Non-substrate	0.314	0
4m	0.96	1.13	Non-substrate	0.41	0
4n	0.971	1.2	Non-substrate	0.344	0

The pan-assay interference (PAINS) assay can identify false positive compounds. Consequently, it prevents the waste of resources and time. PAINS with the SwissADME server was used and the results are summarized in last column of Table 2. The title compounds showed no PAINS.^28,29^ Also Lipinski's rule of five was applied to determine the drug-likeness property of the title compounds (4a–n) (Table S1). All the synthetic compounds (4a–n) passed Lipinski's rule and had acceptable parameters in the range of Lipinski's rule of five, including molecular mass ≤ 500 g mol^−1^, log *P* ≤ 5, hydrogen bond donors ≤ 5, hydrogen bond acceptors ≤ 5, and molar refractivity between 40–130.

**Fig. 3:**
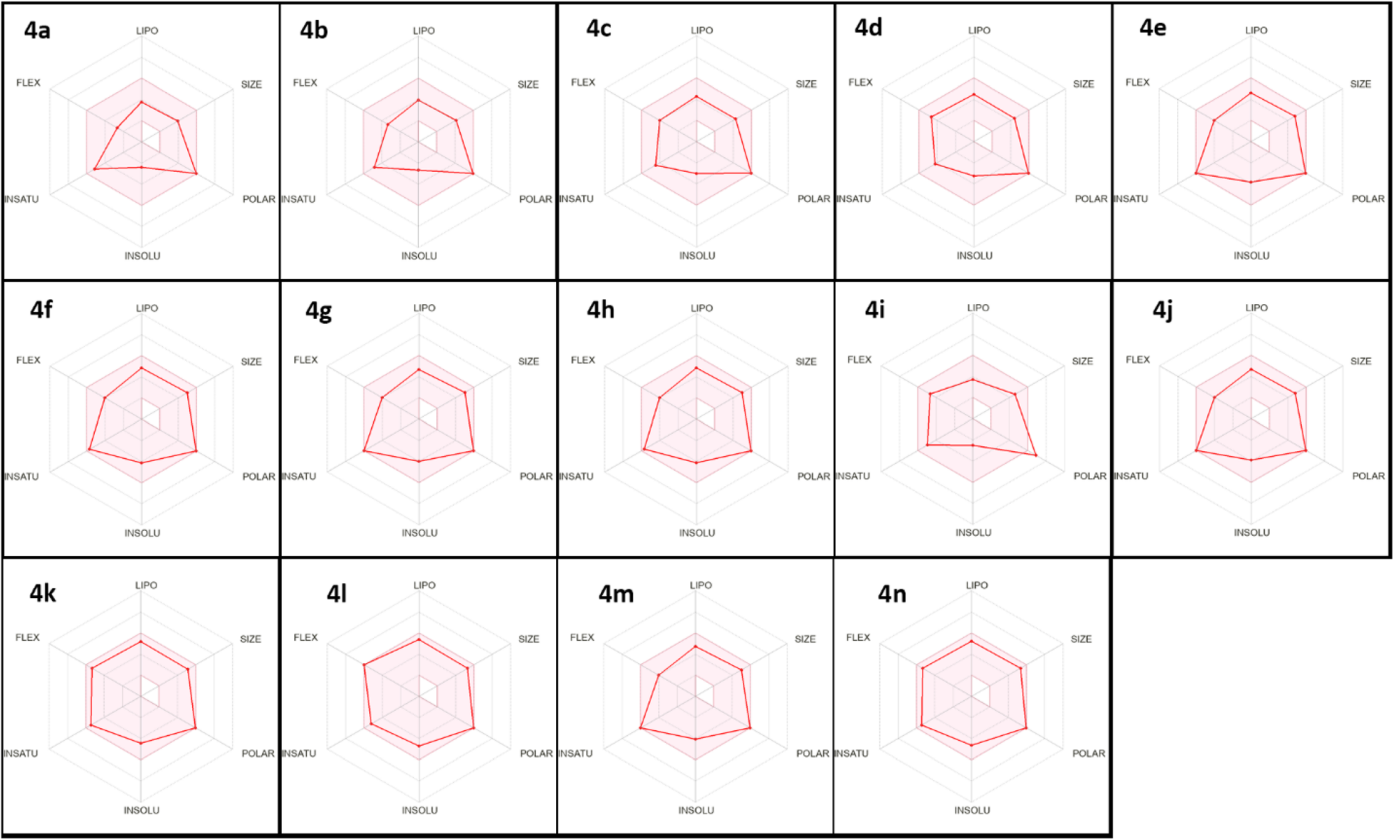
Bioavailability radar plot of compounds 4a–n using the SwissADME predictor.

Using the SwissADME web server based on six different physicochemical parameters consisting of lipophilicity (LIPO), flexibility (FLEX), polarity (POLAR), molecular size (SIZE), solubility (INSOLU), and saturation (INSATU), a radar image was obtained (Fig. 3). The compound located in the pink area and can be considered drug-like.

**Fig. 4:**
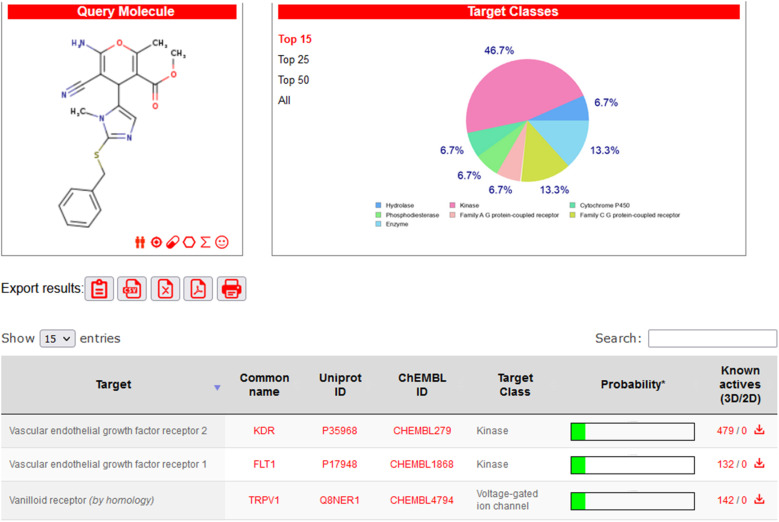
Target predicted for compound 4e by the SwissTargetPrediction online tool.

The SwissTargetPrediction website tool (https://www.swisstargetprediction.ch/) was used to predict the most suitable target for compound 4e. It was found, as seen in Fig. 4, that the anticipated derivatives may have kinase receptor inhibitory action, with a probability of 46.7%. Other receptors were hydrolase, cytochrome P450, phosphodiesterase, family C G-protein coupled receptor, family A G-protein coupled receptor, and enzyme, each with a probability in the range of 6.7% to 13.3%. VEGFR2 belongs to the class V receptor tyrosine kinases and is encoded by the KDR gene. It is predominantly found in vascular endothelial cells, where its expression levels are the highest. Based on SwissTargetPrediction, VEGFR2 may be the target for compound 4e.

The above-mentioned prediction was reconfirmed by CODD-PRED (https://fca_icdb.mpu.edu.mo/codd/works/bioactivity_prediction) (Fig. S57).

#### 2.2.2 Molecular docking

Molecular docking studies were conducted utilizing MOE (Molecular Operating Environment https://www.chemcomp.com) with Amber force field. The X-ray crystal structures of the VEGFR2 kinase domain bound to axitinib were obtained from the Protein Data Bank (PDB 4AG8). Among three PDB codes (4ASE, 3WZD, and 4AG8), based on the docking scores and structure similarity (https://chemtoolshub.com/en/) between title compound and the X-ray ligand, 4AG8 was chosen (Tables S2, S3, and Fig. S58–S60). To validate the docking procedure, re-docking of the native ligand at the active site of VEGFR2 (4AG8) was performed. As illustrated in Fig. S61, there is a significant correspondence between the native and docked ligands, with an RMSD of 0.42 Å observed. The RMSD value was also checked in https://zhanggroup.org/DockRMSD and the result was similar (Fig. S62). Also, we redocked X-ray ligands of 11 different PDB IDs for VEGFR2 (1Y6B, 2OH4, 3C7Q, 3CJF, 3CJG, 3VHE, 3VNT, 3VO3, 5EW3, 6GQO, and 6GQQ) and the RMSD values are summarized in Table S4.

**Fig. 5:**
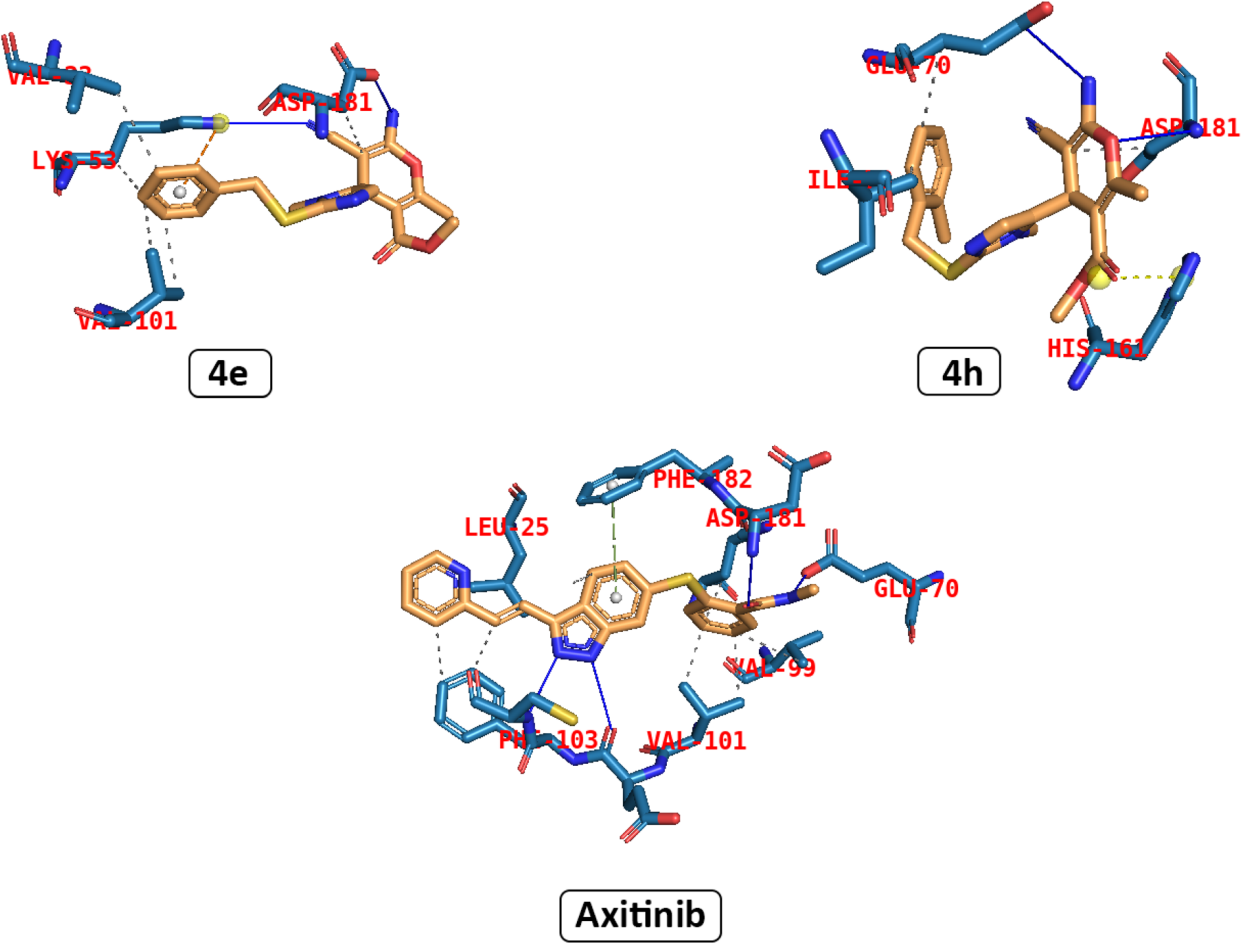
3D description of the interaction between compounds 4e and 4h and axitinib in the crystal structure of VEGFR2.

The interaction of the title compounds in the active site of VEGFR2 was evaluated by docking studies. As illustrated in Fig. 5, the binding mode of axitinib as a co-crystalized ligand with an affinity value of −9.4 kcal mol^−1^ exhibited hydrogen interaction with residues Glu 70 and Asp 181. Also, there were pi interactions with residue Phe 182, and hydrophobic interactions with residues Val 99, Val 101, Leu 25, and Phe 103.

Molecular docking and molecular dynamics (MD) simulations were performed for two compounds, 4e and 4h, which exhibited the best cytotoxicity. Compounds 4e and 4h, similar to axitinib, showed the same interaction. Both compound 4e and 4h showed similar binding modes with the binding affinity of −8.29, and −8.20 kcal mol^−1^, respectively. Compound 4e formed pi interactions with residue Lys 53, hydrogen bonds with residues Asp 181 and Lys 53, and also hydrophobic interactions with residues Val 33 and Val 101. 4h could form a hydrogen bond with residues Glu 70 and Asp 181. 4e formed pi interactions with residue His 161, and hydrophobic interactions with residues Ile 73, Asp 181, and Glu 70.

Also, to confirm that the docking scores are not related to decoys, by means of machine-learning methods it was found that our title compounds are active as VEGFR2 inhibitors. We created a ROC (receiver operating characteristic) curve using the EDock-ML server (https://edock-ml.umsl.edu/),^30^ Firstly, we docked 4e in 11 different PDB IDs for VEGFR2 (1Y6B, 2OH4, 3C7Q, 3CJF, 3CJG, 3VHE, 3VNT, 3VO3, 5EW3, 6GQO, and 6GQQ) using the MOE software. Then, the ROC curve was created (Fig. S63) using EDock-ML (machine learning/model support vector machines, SVM). Also, the specificity and sensitivity for 3 points in the ROC curve are shown in Table S5. The title compound 4e was found to be active against decoy compounds available for VEGFR2 in the EDock-ML server.

#### 2.2.3 Molecular dynamics simulation

**Fig. 6:**
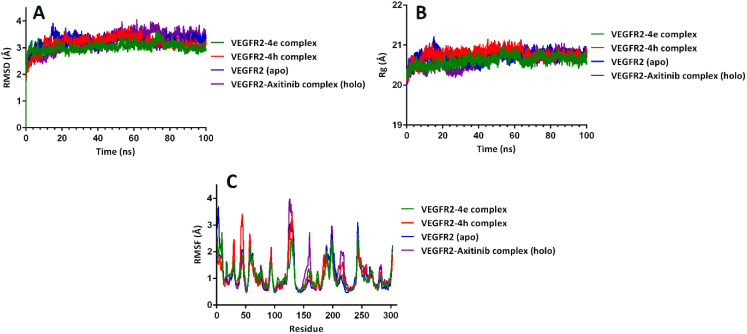
(A) RMSD, (B) *R*_g_, and (C) RMSF of VEGFR2–4e complex, VEGFR2–4h complex, VEGFR2 (apo), VEGFR2–axitinib complex (holo).

Molecular dynamics (MD) simulations were performed for two synthetic compounds, 4e and 4h.^31^ Root mean square deviation (RMSD), root mean square fluctuation (RMSF), and radius of gyration (*R*_g_) are indicators of the complexes formed between 4AG8 and axitinib, 4e, and 4h and show changes in the conformation and dynamics during MD simulations. The mean values (RMSD) for the VEGFR2–4e complex, VEGFR2–4h complex, VEGFR2–axitinib complex (holo), and VEGFR2 (apo) were 3.084 ± 0.142 Å, 3.217 ± 0.174 Å, 3.523 ± 0.149 Å, and 3.235 ± 0.127 Å, respectively. The RMSD value within 100 ns is shown in Fig. 6A. The RMSD value is an indicator of the stability of the modeled complex containing the ligand, where a reduction in the RMSD value indicates a stable system.

The increased compactness and stability of the protein backbone are proven by lower values of *R*_g_. The starting values of *R*_g_ for 4e, 4h, axitinib, and apo form were 20 Å. The *R*_g_ of the protein in the VEGFR2–4e complex, VEGFR2–4h complex, VEGFR2–axitinib complex (holo), and VEGFR2 (apo) was stable without any fluctuation. The mean values (*R*_g_) determined for the complexes involving VEGFR2–4e complex, VEGFR2–4h complex, VEGFR2–axitinib complex (holo), and VEGFR2 (apo) complex were 20.610 ± 0.122 Å, 20.758 ± 0.140 Å, 20.711 ± 0.133 Å, and 20.646 ± 0.186 Å, respectively (Fig. 6B).

The rigidity, stability, and compactness of the ligand–receptor interaction were analyzed using the RMSF plot.^32^ A low RMSF value shows greater rigidity and high stability, whereas a high RMSF value reflects greater flexibility, implying that the residues exhibited reduced stability. As shown in Fig. 6C, most of the residues fluctuated during the simulation of VEGFR2–4e complex, VEGFR2–4h complex, VEGFR2–axitinib complex (holo), and VEGFR2 (apo). The RMSF patterns of the VEGFR2–4e complex, VEGFR2–4h complex, VEGFR2–axitinib complex (holo), and VEGFR2 (apo) were similar, and this result showed that the structure of the protein was not altered during the simulation.

### 2.3. Biological evaluation

#### 2.3.1 MTT assays

To estimate the potential cytotoxicity of the novel compounds 4a–n, we employed three human tumor cell lines (MCF-7, HT29, A2780cis, and CHO). The cells were treated with diverse concentrations of compounds 4a–n, and doxorubicin was chosen as the positive control. The cells were treated with the compounds for 48 h, and then their cytotoxicity and cell viability were assessed using the MTT assay.^33^ The results showed that some compounds generally exhibited higher inhibitory activity against cancer cell lines. According to the MTT results, we measured the IC_50_ values for each synthetic compound using the GraphPad Prism 9.0 software. The results, as demonstrated in Table 3, showed that compounds 4m, 4j, 4h, and 4e had acceptable anticancer activity, with IC_50_ values of 1.90 ± 1.53 µM, 8.72 ± 1.18 µM, 9.44 ± 1.13 µM, and 11.74 ± 1.75 µM in the MCF-7 cell line, respectively. In the HT-29 cell line, compound 4e with IC_50_ of 25.63 ± 1.10 µM and compound 4h with IC_50_ of 30.66 ± 1.27 µM showed high cytotoxicity. Also, we evaluated the toxicity of all the compounds, 4a–n, against normal cells (CHO) and found that most of them exhibited an acceptable safety profile (IC_50_ > 100 µM), except for four compounds, which showed IC_50_ under 100 µM in normal cells (IC_50_, 4g = 72.59 ± 1.11 µM, 4h = 68.14 ± 1.05 µM, 4l = 58.40 ± 1.11 µM, and 4n = 55.21 ± 1.07 µM).

**Table 3:** Cytotoxic activity (IC_50_ (µM)) of compounds 4a–n and doxorubicin against MCF-7, HT29, A2780cis, and CHO cell lines

Compound	Cytotoxicity (IC_50_, µM)			
	MCF-7	HT29	A2780cis	CHO
4a	>100	39.89 ± 1.05	>100	>100
4b	>100	40.52 ± 1.14	>100	>100
4c	90.57 ± 1.22	>100	>100	>100
4d	61.18 ± 1.09	46.43 ± 1.14	>100	>100
4e	11.74 ± 1.75	25.63 ± 1.10	46.40 ± 1.17	>100
4f	84.56 ± 1.27	38.19 ± 1.04	42.87 ± 1.08	>100
4g	14.51 ± 1.12	>100	20.21 ± 1.75	72.59 ± 1.11
4h	9.44 ± 1.13	30.66 ± 1.27	46.44 ± 1.19	68.14 ± 1.05
4i	>100	>100	>100	>100
4j	8.72 ± 1.18	>100	>100	>100
4k	57.92 ± 1.06	64.92 ± 1.06	22.84 ± 1.15	>100
4l	40.59 ± 1.13	72.98 ± 1.27	32.21 ± 1.04	58.40 ± 1.11
4m	1.90 ± 0.53	>100	79.53 ± 1.11	>100
4n	42.87 ± 1.14	>100	21.00 ± 1.06	55.21 ± 1.07
Doxorubicin	5.80 ± 1.18	4.11 ± 1.30	1.56 ± 0.34	2.17 ± 1.09

#### 2.3.2 Apoptosis assay

**Fig. 7:**
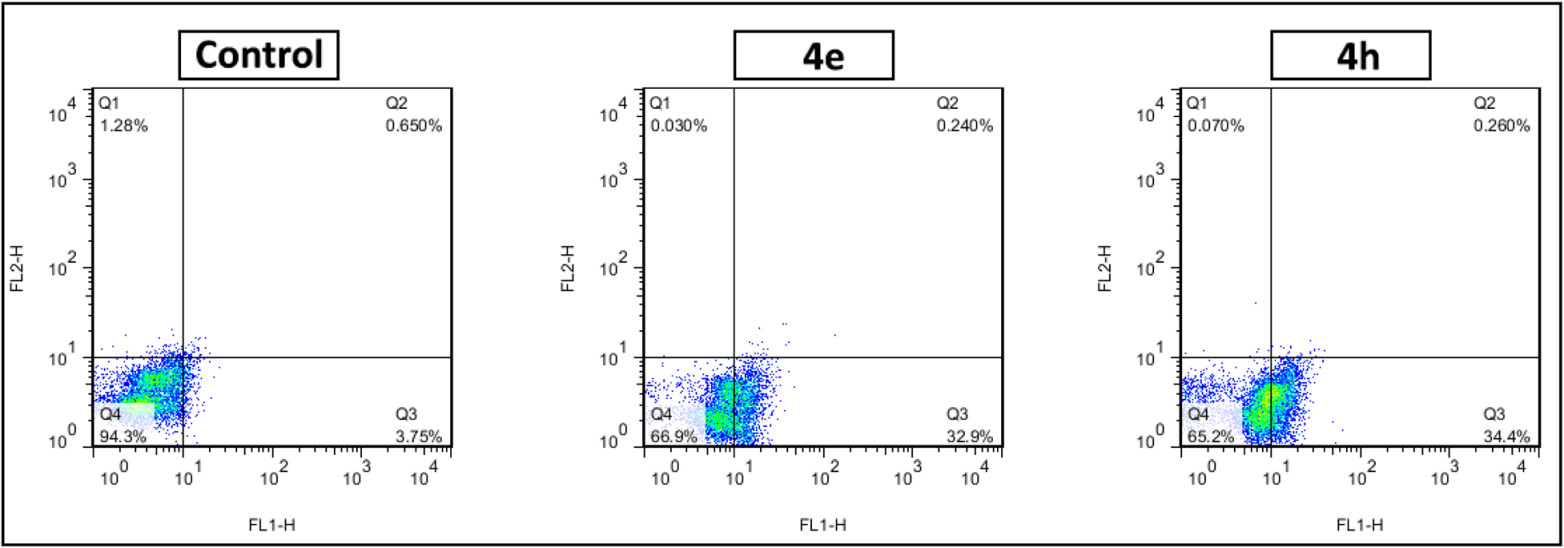
Cell apoptosis induced by 4e and 4h in MCF-7 cells.

MCF-7 cells were treated with the IC_50_ concentration of 4e of 11.74 ± 1.75 µM or IC_50_ concentrations of 4h of 9.44 ± 1.13 µM for 24 h, and the ratio of apoptotic cells was studied by flow cytometry using annexin-V and PI.^34,35^ As demonstrated in Fig. 7, the amount of late and early apoptotic MCF-7 cells (*Q*_2_ + *Q*_3_) after treatment with compound 4e was 33.14%, and treatment with compound 4h resulted in late and early apoptosis of 34.66%. The results showed that compounds 4e and 4h promoted apoptosis in MCF-7 cells more than the control group (*Q*_2_ + *Q*_3_ = 4.4%).

#### 2.3.3 Colony formation assay

**Fig. 8:**
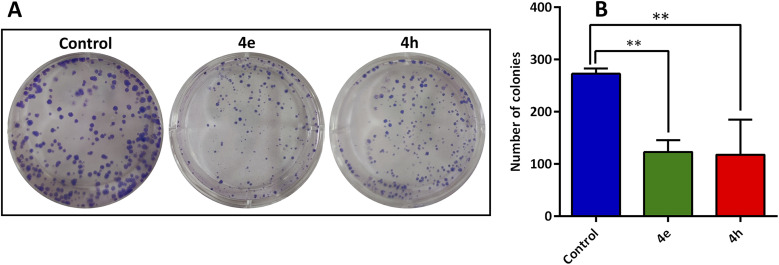
(A) Pictures of colony formation of MCF-7 cells treated with 4e and 4h, (B) number of colonies were shown as graph; **p ≤ 0.01, *vs.* control group.

One of the methods used to assess cell proliferation is the colony formation assay.^36,37^ To determine the ability of adherent cells to form and grow colonies in an *in vitro* environment, a colony assay was applied on a plate. The effect of treatment with compounds 4e and 4h on colony formation of breast cancer cells was investigated at their IC_50_ concentration. MCF-7 cells were treated with IC_50_ concentrations (4e = 11.74 ± 1.75 µM and 4h = 9.44 ± 1.13 µM) of the compounds for 48 h, and then the number of cell colonies was counted and proliferation inhibition was analyzed. As can be seen in Fig. 8, the number of cell colonies decreased in the treatment groups in contrast to the control group.

## 3. Conclusion

In this study, a new group of imidazole-pyran compounds was conceptualized, synthesized and tested *in silico* and *in vitro* as VEGFR2 kinase inhibitors. Among the synthesized derivatives, 4e and 4h showed the highest cytotoxicity with IC_50_ values of 7.69 ± 1.99 and 11.74 ± 1.75 µM on the MCF-7 cell line, respectively. Molecular docking study of compounds 4e and 4h within the VEGFR2 binding site showed important interactions through specific residues, consisting of hydrophobic interactions and hydrogen bonds. In addition, molecular dynamics simulations of compounds 4e and 4h showed that they formed strong and stable interactions with essential residues, similar to axitinib. In addition, the complexes of 4e–VEGFR2 and 4h–VEGFR2 were stable during the simulation study. The ADMET and physicochemical parameters exhibited the appropriate parameters for the synthetic compounds. Our results suggest that compounds 4e and 4h can serve as drug candidates in the creation of more potent anticancer medications.

## 4. Materials and methods

All solvents, basic materials, and reagents were bought from Sigma-Aldrich Chemicals. Melting points were determined with a Stuart SMP3 melting point apparatus (UK). Infrared (IR) spectra were recorded using a PerkinElmer Model 1420 spectrometer (KBr disks, USA). ^1^H NMR and ^13^C NMR spectra were recorded in DMSO-*d*_6_ using a Bruker FT-300 MHz instrument. A Shimadzu UFLC-AB Sciex 3200 QTRAP mass spectrometer was employed to obtain mass spectra. Elemental analysis was performed using a Thermo Finnigan Flash EA microanalyzer (USA). All used cell lines, including HT29 (human colon cancer cell line), MCF-7 (human breast cancer cell line), CHO (Chinese hamster ovary cell line), and A2780cis (cisplatin-resistant ovarian cancer cell line), were purchased from the Pasteur Institute in Tehran, Iran.

### 4.1. Chemistry and general method for the synthesis of imidazole-pyran derivatives (4a–n)

A combination of 1.0 mmol imidazole-5-carbaldehyde (1a–n), 1.0 mmol malononitrile (2) and 1.0 mmol methyl acetoacetate (3) was prepared in ethanol (3 mL) with the addition of a few drops of piperidine. This mixture was stirred at ambient temperature using a magnetic stirrer for 5 min. The reaction progress was tracked using TLC. Upon completion, the mixture was filtered, and washed using 2 mL of cold ethanol and dried without additional purification. The product was characterized by FTIR, ^1^H NMR, ^13^C NMR, elemental analysis and mass spectrometry.

#### 4.1.1 Methyl-6-amino-5-cyano-2-methyl-4-(1-methyl-2-(methylthio)-1*H*-imidazol-5-yl)-4*H*-pyran-3-carboxylate (4a)

White powder; yield 57%; mp: 199–201 °C, IR (KBr) (*ν*_max_/cm^−1^): 2199.3 (CN), 1744.8 (C=O); ^1^H NMR (300 MHz, DMSO-*d*_6_): *δ*_H_ 7.04 (s, 2H), 6.65 (s, 1H), 4.51 (s, 1H), 3.59 (s, 3H), 3.52 (s, 3H), 2.50 (s, 3H), 2.32 (s, 3H); ^13^C NMR (75 MHz, DMSO-*d*_6_): *δ*_C_ 166.27, 159.32, 157.77, 141.95, 137.38, 127.16, 120.22, 105.75, 55.83, 52.27, 30.83, 29.53, 18.75, 15.89; MS (ESI) *m*/*z*: [M + H]^+^: 321.073; anal. calcd for C_14_H_16_N_4_O_3_S: C, 52.49; H, 5.03; N, 17.49; found: C, 52.43; H, 5.11; N, 17.42%.

#### 4.1.2 Methyl-6-amino-5-cyano-4-(2-(ethylthio)-1-methyl-1*H*-imidazol-5-yl)-2-methyl-4*H*-pyran-3-carboxylate (4b)

White powder; yield 53%; mp: 191–193 °C, IR (KBr) (*ν*_max_/cm^−1^): 2196.5 (CN), 1720.8 (C=O); ^1^H NMR (300 MHz, DMSO-*d*_6_): *δ*_H_ 7.04 (s, 2H), 6.69 (s, 1H), 4.52 (s, 1H), 3.57 (m, 6H), 3.02–2.89 (m, 2H), 2.32 (s, 3H), 1.19 (m, 3H); ^13^C NMR (75 MHz, DMSO-*d*_6_): *δ*_C_ 166.27, 159.17, 157.73, 140.29, 137.64, 127.55, 120.18, 105.65, 55.79, 52.22, 31.08, 29.64, 28.43, 18.73, 15.38; MS (ESI) *m*/*z*: [M + H]^+^: 335.13; anal. calcd for C_15_H_18_N_4_O_3_S: C, 53.88; H, 5.43; N, 16.76; found: C, 53.79; H, 5.52; N, 16.80%.

#### 4.1.3 Methyl-6-amino-5-cyano-2-methyl-4-(1-methyl-2-(propylthio)-1*H*-imidazol-5-yl)-4*H*-pyran-3-carboxylate (4c)

White powder; yield 51%; mp: 182–183 °C, IR (KBr) (*ν*_max_/cm^−1^): 2196.6 (CN), 1716.4 (C=O); ^1^H NMR (300 MHz, DMSO-*d*_6_): *δ*_H_ 7.04 (s, 2H), 6.68 (s, 1H), 4.52 (d, 1H), 3.57 (d, 6H), 2.93 (t, 2H), 2.35–2.29 (m, 3H), 1.55 (m, 2H), 0.93 (t, 3H); ^13^C NMR (75 MHz, DMSO-*d*_6_): *δ*_C_ 166.27, 159.17, 157.72, 140.52, 137.58, 127.48, 120.18, 105.66, 55.80, 52.21, 35.97, 31.04, 29.64, 23.03, 18.73, 13.40; MS (ESI) *m*/*z*: [M + H]^+^: 349.119; anal. calcd for C_16_H_20_N_4_O_3_S: C, 55.16; H, 5.79; N, 16.08; found: C, 55.19; H, 5.90; N, 16.12%.

#### 4.1.4 Methyl-6-amino-4-(2-(butylthio)-1-methyl-1*H*-imidazol-5-yl)-5-cyano-2-methyl-4*H*-pyran-3-carboxylate (4d)

White powder; yield 54%; mp: 180–181 °C, IR (KBr) (*ν*_max_/cm^−1^): 2195 (CN), 1717.3 (C=O); ^1^H NMR (300 MHz, DMSO-*d*_6_): *δ*_H_ 7.03 (s, 2H), 6.68 (s, 1H), 4.52 (s, 1H), 3.57 (d, 6H), 2.95 (t, 2H), 2.32 (s, 3H), 1.58–1.27 (m, 4H), 0.86 (t, 3H); ^13^C NMR (75 MHz, DMSO-*d*_6_): *δ*_C_ 166.27, 159.15, 157.71, 140.52, 137.57, 127.52, 120.16, 105.65, 55.81, 52.20, 33.75, 31.76, 31.04, 29.65, 21.54, 18.73, 13.92; MS (ESI) *m*/*z*: [M + H]^+^: 363.089; anal. calcd. For C_17_H_22_N_4_O_3_S: C, 56.34; H, 6.12; N, 15.46; found: C, 56.39; H, 6.20; N, 15.42%.

#### 4.1.5 Methyl-6-amino-4-(2-(benzylthio)-1-methyl-1*H*-imidazol-5-yl)-5-cyano-2-methyl-4*H*-pyran-3-carboxylate (4e)

White powder; yield 30%; mp: 184–185 °C, IR (KBr) (*ν*_max_/cm^−1^): 2199.3 (CN), 1719 (C=O); ^1^H NMR (300 MHz, DMSO-*d*_6_): *δ*_H_ 7.35–7.20 (m, 3H), 7.12–7.03 (m, 4H), 6.76 (s, 1H), 4.53–4.46 (m, 1H), 4.11 (s, 2H), 3.59 (s, 3H), 3.23 (s, 3H), 2.34–2.28 (m, 3H); ^13^C NMR (75 MHz, DMSO-*d*_6_): *δ*_C_ 166.18, 159.30, 157.87, 139.65, 138.46, 137.38, 129.05, 128.97, 128.20, 127.62, 120.05, 105.47, 55.43, 52.23, 39.52, 30.74, 29.90, 18.74; MS (ESI) *m*/*z*: [M + H]^+^: 397.11; anal. calcd for C_20_H_20_N_4_O_3_S: C, 60.59; H, 5.08; N, 14.13; found: C, 60.69; H, 5.02; N, 14.19%.

#### 4.1.6 Methyl-6-amino-5-cyano-2-methyl-4-(1-methyl-2-((4-methylbenzyl)thio)-1*H*-imidazol-5-yl)-4*H*-pyran-3-carboxylate (4f)

White powder; yield 38%; mp: 183–184 °C, IR (KBr) (*ν*_max_/cm^−1^): 2197.3 (CN), 1718.3 (C=O); ^1^H NMR (300 MHz, DMSO-*d*_6_): *δ*_H_ 7.13–7.03 (m, 4H), 6.96 (d, 2H), 6.76 (s, 1H), 4.50 (s, 1H), 4.06 (s, 2H), 3.58 (s, 3H), 3.23 (s, 3H), 2.30 (d, 6H); ^13^C NMR (75 MHz, DMSO-*d*_6_): *δ*_C_ 166.17, 159.27, 157.85, 139.80, 137.29, 136.87, 135.35, 129.51, 128.95, 128.20, 120.01, 105.45, 55.47, 52.20, 39.32, 30.73, 29.95, 21.21, 18.72; MS (ESI) *m*/*z*: [M + H]^+^: 411.095; anal. calcd for C_21_H_22_N_4_O_3_S: C, 61.45; H, 5.40; N, 13.65; found: C, 61.50; H, 5.46; N, 13.72%.

#### 4.1.7 Methyl-6-amino-5-cyano-4-(2-((3-fluorobenzyl)thio)-1-methyl-1*H*-imidazol-5-yl)-2-methyl-4*H*-pyran-3-carboxylate (4g)

White powder; yield 48%; mp: 178–180 °C, IR (KBr) (*ν*_max_/cm^−1^): 2195 (CN), 1719.2 (C=O); ^1^H NMR (300 MHz, DMSO-*d*_6_): *δ*_H_ 7.37–7.23 (m, 1H), 7.13–7.02 (m, 4H), 6.89–6.80 (m, 1H), 6.75 (s, 1H), 4.50 (s, 1H), 4.15 (s, 2H), 3.58 (s, 3H), 3.37 (s, 3H), 2.32 (s, 3H); ^13^C NMR (75 MHz, DMSO-*d*_6_): *δ*_C_ 166.17, 164.07, 159.28, 157.93, 141.44, 141.34, 139.36, 137.71, 130.84, 130.73, 128.06, 125.12, 125.09, 120.07, 116.04, 115.76, 114.59, 114.32, 105.50, 55.54, 52.20, 38.46, 30.89, 29.82, 18.75; MS (ESI) *m*/*z*: [M + H]^+^: 415.04; anal. calcd for C_20_H_19_FN_4_O_3_S: C, 57.96; H, 4.62; N, 13.52; found: C, 57.99; H, 4.72; N, 13.61%.

#### 4.1.8 Methyl-6-amino-5-cyano-2-methyl-4-(1-methyl-2-((2-methylbenzyl)thio)-1*H*-imidazol-5-yl)-4*H*-pyran-3-carboxylate (4h)

White powder; yield 43%; mp: 178–180 °C, IR (KBr) (*ν*_max_/cm^−1^): 2194.7 (CN), 1720.7 (C=O); ^1^H NMR (300 MHz, DMSO-*d*_6_): *δ*_H_ 7.21–7.13 (m, 2H), 7.13–7.01 (m, 3H), 6.89 (d, 1H), 6.75 (s, 1H), 4.48 (d, 1H), 4.09 (s, 2H), 3.43–3.31 (m, 3H), 3.16 (s, 3H), 2.34–2.28 (m, 3H), 2.23 (s, 3H); ^13^C NMR (75 MHz, DMSO-*d*_6_): *δ*_C_ 166.12, 159.38, 157.99, 139.48, 137.39, 136.64, 136.08, 130.69, 129.94, 128.23, 127.91, 126.56, 120.01, 105.46, 55.36, 52.24, 37.76, 30.57, 29.96, 18.90, 18.75; MS (ESI) *m*/*z*: [M + H]^+^: 411.073; anal. calcd for C_21_H_22_N_4_O_3_S: C, 61.45; H, 5.40; N, 11.96; found: C, 61.50; H, 5.45; N, 11.88%.

#### 4.1.9 Methyl-6-amino-5-cyano-4-(2-((2-methoxy-2-oxoethyl)thio)-1-methyl-1*H*-imidazol-5-yl)-2-methyl-4*H*-pyran-3-carboxylate (4i)

White powder; yield 59%; mp: 185–187 °C, IR (KBr) (*ν*_max_/cm^−1^): 2199.3 (CN), 1744.8 (C=O); ^1^H NMR (300 MHz, DMSO-*d*_6_): *δ*_H_ 7.05 (s, 2H), 6.68 (s, 1H), 4.52 (s, 1H), 3.86 (s, 2H), 3.70–3.55 (m, 9H), 2.32 (s, 3H); ^13^C NMR (75 MHz, DMSO-*d*_6_): *δ*_C_ 169.77, 166.21, 159.30, 157.92, 139.32, 137.98, 127.77, 120.16, 105.64, 55.69, 52.76, 52.25, 35.75, 31.11, 29.60, 18.75; MS (ESI) *m*/*z*: [M + H]^+^: 379.03; anal. calcd for C_16_H_18_N_4_O_5_S: C, 50.79; H, 4.79; N, 14.81; found: C, 50.69; H, 4.90; N, 14.92%.

#### 4.1.10 Methyl-6-amino-4-(1-benzyl-2-(methylthio)-1*H*-imidazol-5-yl)-5-cyano-2-methyl-4*H*-pyran-3-carboxylate (4j)

White powder; yield 60%; mp: 163–166 °C, IR (KBr) (*ν*_max_/cm^−1^): 2190.7 (CN), 1723.4 (C=O); ^1^H NMR (300 MHz, DMSO-*d*_6_): *δ*_H_ 7.37 (m, 3H), 7.17–7.00 (m, 4H), 6.79 (s, 1H), 5.47–5.31 (m, 1H), 5.22 (d, 1H), 4.51 (s, 1H), 3.48 (s, 3H), 2.49 (s, 3H), 2.21 (s, 3H); ^13^C NMR (75 MHz, DMSO-*d*_6_): *δ*_C_ 165.99, 159.82, 158.44, 143.32, 137.02, 136.89, 129.01, 127.75, 126.49, 120.28, 105.64, 55.84, 52.01, 47.32, 29.57, 18.66, 16.05; MS (ESI) *m*/*z*: [M + H]^+^: 397.11; anal. calcd for C_20_H_20_N_4_O_3_S: C, 60.59; H, 5.08; N, 14.13; found: C, 60.69; H, 5.13; N, 14.19%.

#### 4.1.11 Methyl-6-amino-4-(1-benzyl-2-(propylthio)-1*H*-imidazol-5-yl)-5-cyano-2-methyl-4*H*-pyran-3-carboxylate (4k)

White powder; yield 59%; mp: 184–185 °C, IR (KBr) (*ν*_max_/cm^−1^): 2199.3 (CN), 1723.4 (C=O); ^1^H NMR (300 MHz, DMSO-*d*_6_): *δ*_H_7.33 (m, 3H), 7.07–6.94 (m, 4H), 6.76 (s, 1H), 5.35 (d, 1H), 5.23 (d, 1H), 4.46 (s, 1H), 3.43 (s, 3H), 2.90 (t, 2H), 2.17 (s, 3H), 1.55 (m, 2H), 0.88 (t, 3H); ^13^C NMR (75 MHz, DMSO-*d*_6_): *δ*_C_ 166.00, 159.79, 158.38, 142.14, 137.23, 136.87, 128.98, 128.01, 127.70, 126.40, 120.23, 105.63, 55.79, 51.99, 47.39, 35.81, 29.65, 22.96, 18.66, 13.43; MS (ESI) *m*/*z*: [M + H]^+^: 425.142; anal. calcd for C_22_H_24_N_4_O_3_S: C, 62.25; H, 5.70; N, 13.20; found: C, 62.29; H, 5.78; N, 13.32%.

#### 4.1.12 Methyl-6-amino-4-(1-benzyl-2-(butylthio)-1*H*-imidazol-5-yl)-5-cyano-2-methyl-4*H*-pyran-3-carboxylate (4l)

White powder; yield 49%; mp: 166–168 °C, IR (KBr) (*ν*_max_/cm^−1^): 2199 (CN), 1722.7 (C=O); ^1^H NMR (300 MHz, DMSO-*d*_6_): *δ*_H_ 7.35 (m, 2H), 7.33–7.23 (m, 1H), 7.07–6.94 (m, 4H), 6.77 (s, 1H), 5.35 (d, 1H), 5.23 (d, 1H), 4.47 (s, 1H), 3.43 (s, 3H), 2.92 (t, 2H), 2.17 (s, 3H), 1.58–1.42 (m, 2H), 1.29 (m, 2H), 0.83 (t, 3H); ^13^C NMR (75 MHz, DMSO-*d*_6_): *δ*_C_ 166.00, 159.78, 158.39, 142.15, 137.24, 136.87, 128.97, 128.03, 127.70, 126.40, 120.22, 105.61, 55.79, 51.98, 47.39, 33.63, 31.67, 29.65, 21.58, 18.66, 13.90; MS (ESI) *m*/*z*: [M + H]^+^: 439.185; anal. calcd for C_23_H_26_N_4_O_3_S: C, 62.99; H, 5.98; N, 12.78; found: C, 62.89; H, 5.92; N, 12.82%.

#### 4.1.13 Methyl-6-amino-5-cyano-4-(1-(4-fluorobenzyl)-2-(methylthio)-1*H*-imidazol-5-yl)-2-methyl-4*H*-pyran-3-carboxylate (4m)

White powder; yield 56%; mp: 190–191 °C, IR (KBr) (*ν*_max_/cm^−1^): 2198 (CN), 1720.4 (C=O); ^1^H NMR (300 MHz, DMSO-*d*_6_): *δ*_H_ 7.19 (m, 2H), 7.04 (d, 4H), 6.75 (d, 1H), 5.29 (d, 1H), 5.16 (d, 1H), 4.48 (s, 1H), 3.48 (d, 3H), 2.46 (d, 3H), 2.20 (s, 3H); ^13^C NMR (75 MHz, DMSO-*d*_6_): *δ*_C_ 166.03, 159.71, 158.35, 143.23, 136.82, 133.20, 128.64, 128.53, 127.81, 120.19, 115.98, 115.69, 105.64, 55.83, 52.08, 46.66, 29.55, 18.71, 15.99; MS (ESI) *m*/*z*: [M + H]^+^: 415.061; anal. calcd for C_20_H_19_FN_4_O_3_S: C, 57.96; H, 4.62; N, 13.52; found: C, 57.90; H, 4.66; N, 13.42%.

#### 4.1.14 Methyl-6-amino-5-cyano-4-(1-(4-fluorobenzyl)-2-(propylthio)-1*H*-imidazol-5-yl)-2-methyl-4*H*-pyran-3-carboxylate (4n)

White powder; yield 59%; mp: 176–178 °C, IR (KBr) (*ν*_max_/cm^−1^): 2198 (CN), 1721.3 (C=O); ^1^H NMR (300 MHz, DMSO-*d*_6_): *δ*_H_ 7.26–7.13 (m, 2H), 7.01 (m, 4H), 6.76 (d, 1H), 5.32 (d, 1H), 5.20 (d, 1H), 4.47 (s, 1H), 3.46 (d, 3H), 2.92 (t, 2H), 2.19 (s, 3H), 1.55 (m, 2H), 0.88 (m, 3H); ^13^C NMR (75 MHz, DMSO-*d*_6_): *δ*_C_ 166.04, 159.67, 158.28, 142.04, 136.80, 133.43, 128.53, 128.42, 128.10, 120.14, 115.94, 115.65, 105.63, 55.79, 52.05, 46.72, 40.82, 40.54, 40.26, 39.98, 39.71, 39.43, 39.15, 35.77, 29.62, 22.96, 18.71, 13.42; MS (ESI) *m*/*z*: [M + H]^+^: 443.109; anal. calcd for C_22_H_23_FN_4_O_3_S: C, 59.71; H, 5.24; N, 12.66; found: C, 59.69; H, 5.31; N, 12.79%.

### 4.2. *In silico* studies

#### 4.2.1 *In silico* prediction of ADMET properties, physicochemical properties, and target prediction

The estimation of ADMET properties, physicochemical properties, and Lipinski's rule of five were performed using the webtools Deep-PK (https://biosig.lab.uq.edu.au/deeppk/), SwissADME (https://www.swissadme.ch/), and SCFBio https://scfbio-iitd.res.in/software/drugdesign/lipinski.jsp, respectively. Target prediction was performed with the SwissTargetPrediction webtool (https://www.swisstargetprediction.ch/).

#### 4.2.2 Molecular docking

Molecular docking was performed using the Molecular Operating Environment (MOE, Chemical Computing Group Inc.: Montreal, [https://www.chemcomp.com]) software. The complex structure of the protein Vascular Endothelial Growth Factor Receptor 2 (VEGFR2) (PDB ID 4AG8) was extracted from the protein database at a resolution of less than 2.0 Å. A new docking experiment was performed with the crystallographic inhibitor axitinib in the active site of the enzyme to investigate the docking protocol. Docking was then applied to 4e and 4h as the most effective analogues. Ligand–protein interactions were described using PLIP (https://plip-tool.biotec.tu-dresden.de/plip-web/plip/index), the resulting PSE files were downloaded, and we used PyMOL (https://www.pymol.org/) to visualize the 3D structures.

#### 4.2.3 Molecular dynamics simulation

The NAMD3 software was used for the molecular dynamics simulations in conjunction with the force field CHARMM 27 (par_all27_prot_lipid_na.inp). During the simulation, the system was enclosed in a cubic box with a size of 60 Å on each side, and also filled with TIP3P water molecules. The system was rendered inactive through the introduction of sodium and chloride ions. The simulation run time was 100 ns (ref. 37) and the dynamics studies were performed at atmospheric pressure (1 bar) and temperature of 300 K. The Visual Molecular Dynamics (VMD) software was utilized to examine the stability of the molecular dynamics trajectories.

### 4.3. Biological evaluation

#### 4.3.1 Cell culture and viability assays

In 96-well plates, the cells (MCF-7, A2780cis, HT29 and CHO) were seeded at a density of 5000 cells per well and incubated for 24 h in a cell culture incubator. Then, for 48 h, the cells were treated with diverse concentrations of the compounds (4a–n) and doxorubicin. After adding 10 µL MTT solution (0.5 mg mL^−1^) to each well, the cells were incubated for 4 h in a cell culture incubator. After 4 h, the culture medium was changed with 100 µL DMSO. At last, the absorbance was determined using a BioTek ELx800 microplate reader (550 nm and 630 nm).

#### 4.3.2 Apoptosis assay

In 6-well plates, MCF-7 cells were seeded at a density of 2 × 10^5^ cells per hole and incubated for 24 h. Then, the cells were treated with IC_50_ concentrations of 4e of 11.74 µM and 4h of 9.44 µM for 24 h. In next step, the cells were detached using trypsin and washed with PBS. After twice washing, the cells were resuspended in a volume of 500 µL of binding buffer. In this step, to each well 1 µL annexin V-FITC was added, and then 1 µL propidium iodide and mixed moderately and incubated for 15 min. Detection was carried out using a BD FACSCalibur instrument flow cytometer.

#### 4.3.3 Colony formation assay

In a 6-well plate, MCF-7 cells were seeded at 1000 cells per well and cultured for 24 h, followed by treatment with IC_50_ concentrations of 4e of 11.74 µM and 4h of 9.44 µM for 48 h at 37 °C. The cells were subsequently cultured for 10 days at 37 °C. Then, the cells were washed 3 times with PBS, and subsequently fixed in methanol. Giemsa stain (Sigma-Aldrich) (1/10 distilled water) was used to stain the cells (15 min), and the number of colonies was counted visually.

## Conflicts of interest

The authors declare that they have no conflicts of interest.

## Ethical statement

This work was approved in the ethics committee of Mashhad University of Medical Science with accession link https://IR.MUMS.PHARMACY.REC.1402.074.

## Supplementary Material

RA-016-D5RA05264E-s001

## Data Availability

All data are within the manuscript and its supplementary information (SI). Supplementary information is available. See DOI: https://doi.org/10.1039/d5ra05264e.
