# Supplementary material for: Multicomponent synthesis of novel imidazole-pyran derivatives: in silico and in vitro studies
Source: RSC Adv. 2026 Jan 2;16(1):574–85. doi: 10.1039/d5ra05264e (PMC12757938; doi:10.1039/d5ra05264e)
Supplement: RA-016-D5RA05264E-s001 [file RA-016-D5RA05264E-s001.pdf]

Supplementary Information (SI) for RSC Advances.
This journal is © The Royal Society of Chemistry 2025

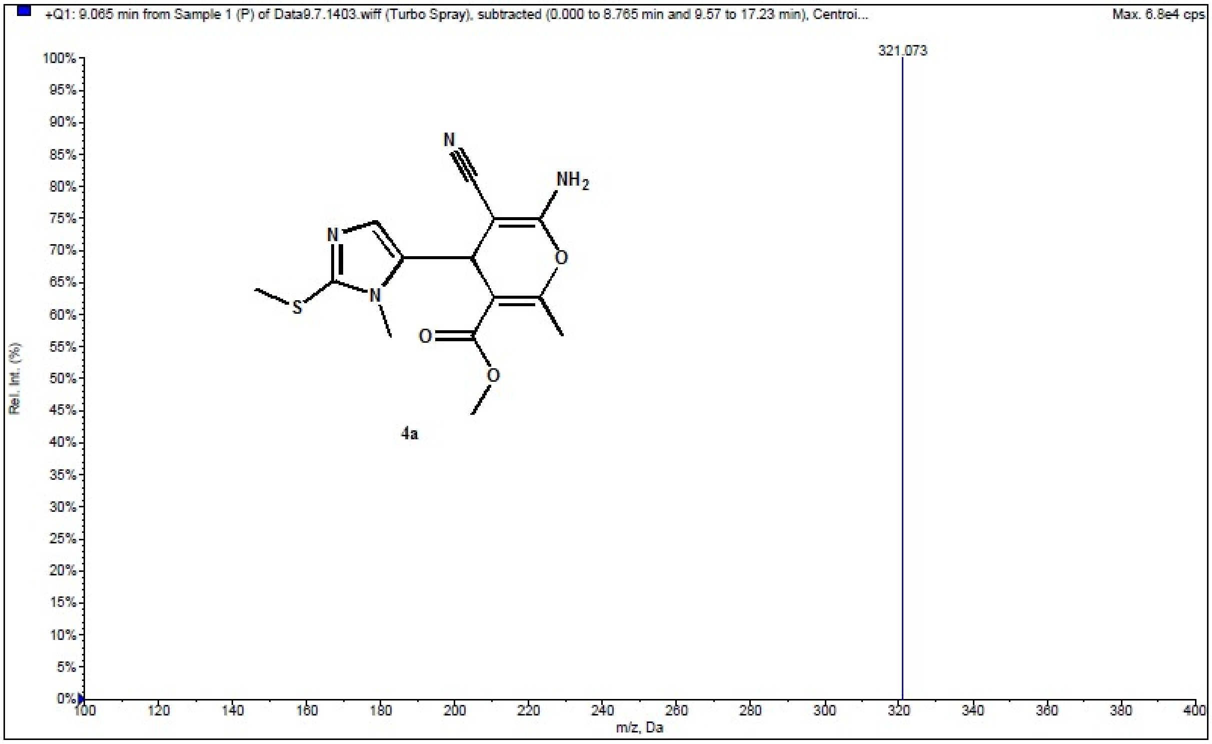

Figure S1: Mass spectrum of derivative **4a**

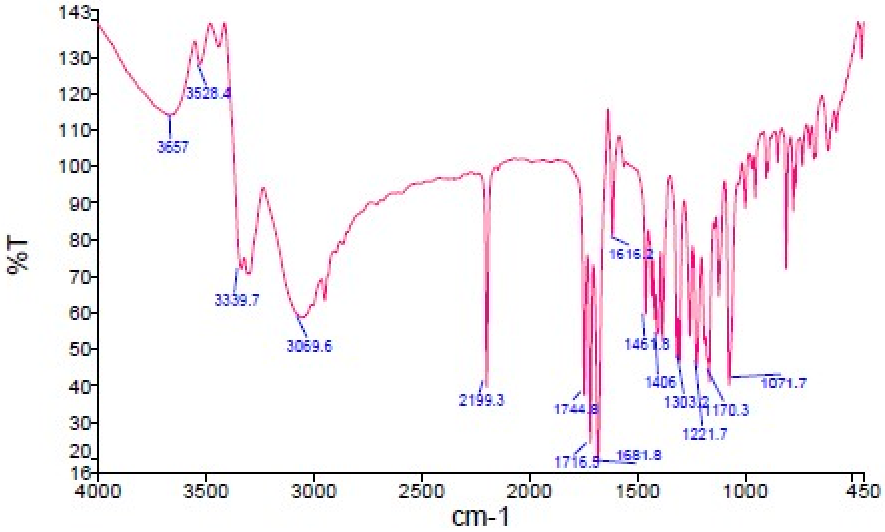

Figure S2: FTIR spectrum of derivative **4a**

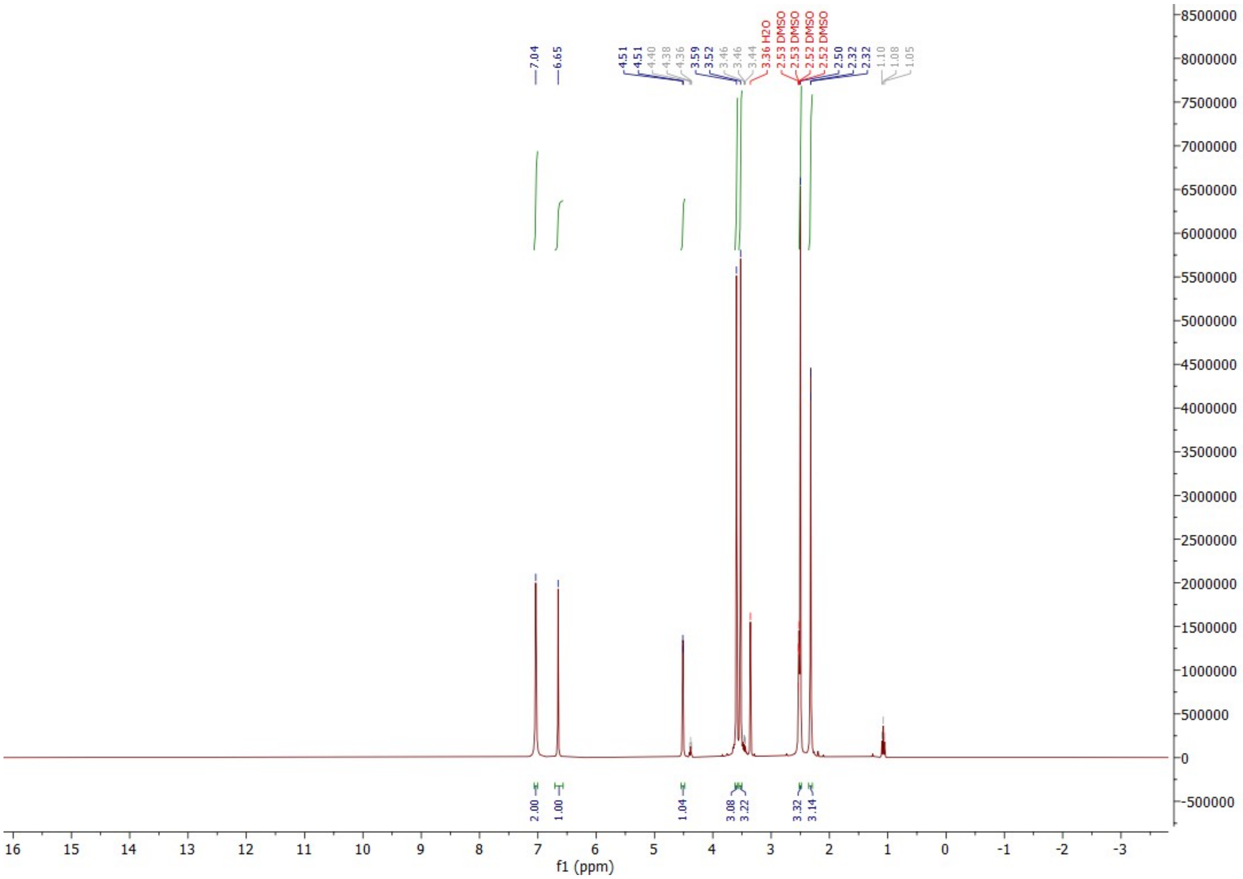

Figure S3: ^{1}HNMR spectrum of derivative **4a**

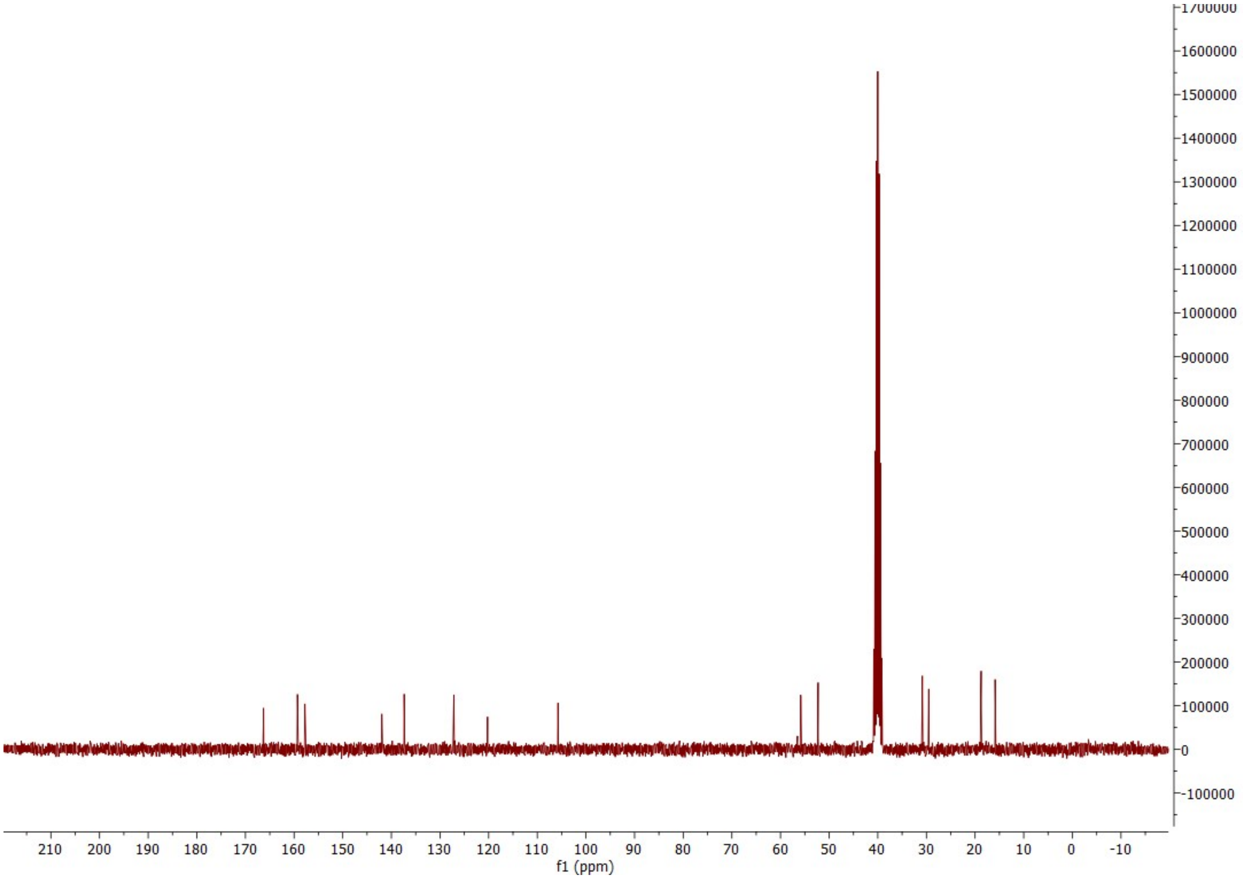

Figure S4: ^{13}CNMR spectrum of derivative **4a**

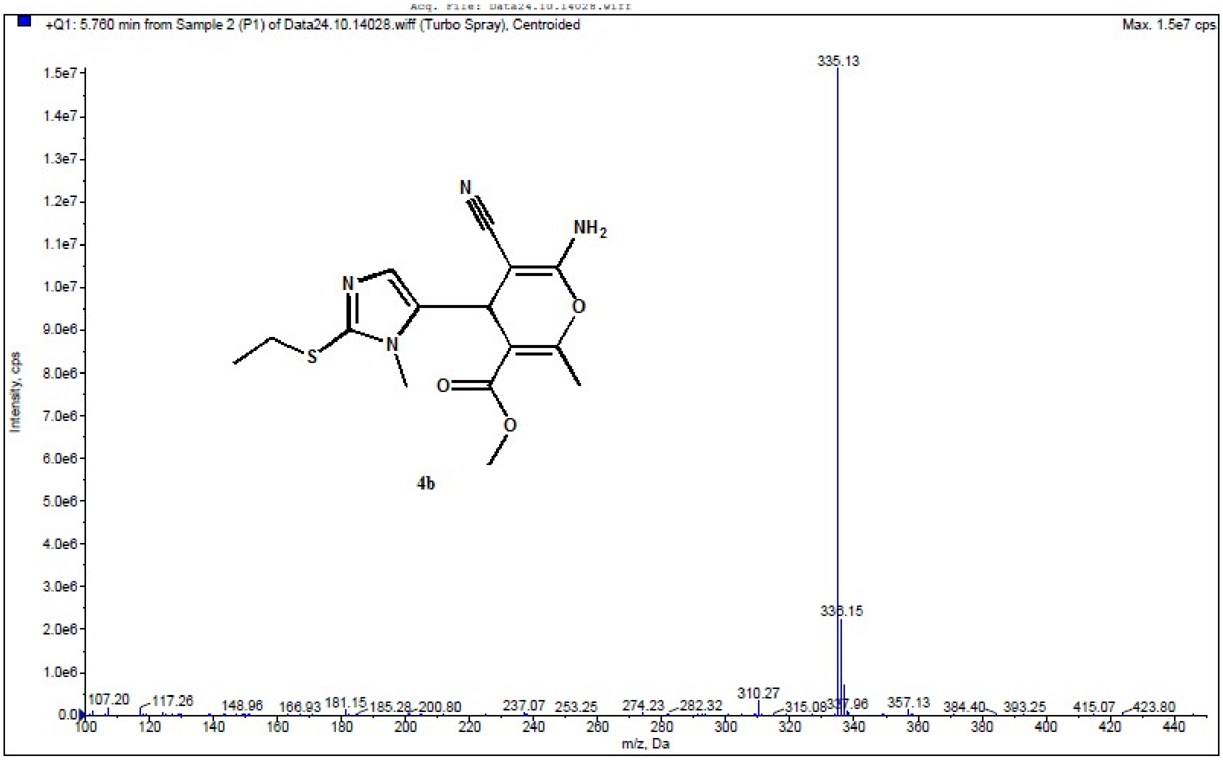

Figure S5: Mass spectrum of derivative **4b**

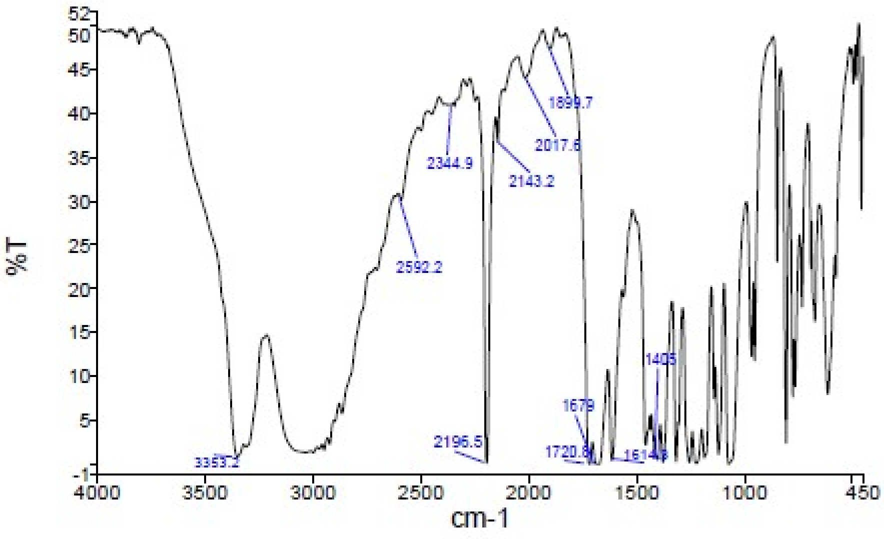

Figure S6: FTIR spectrum of derivative **4b**

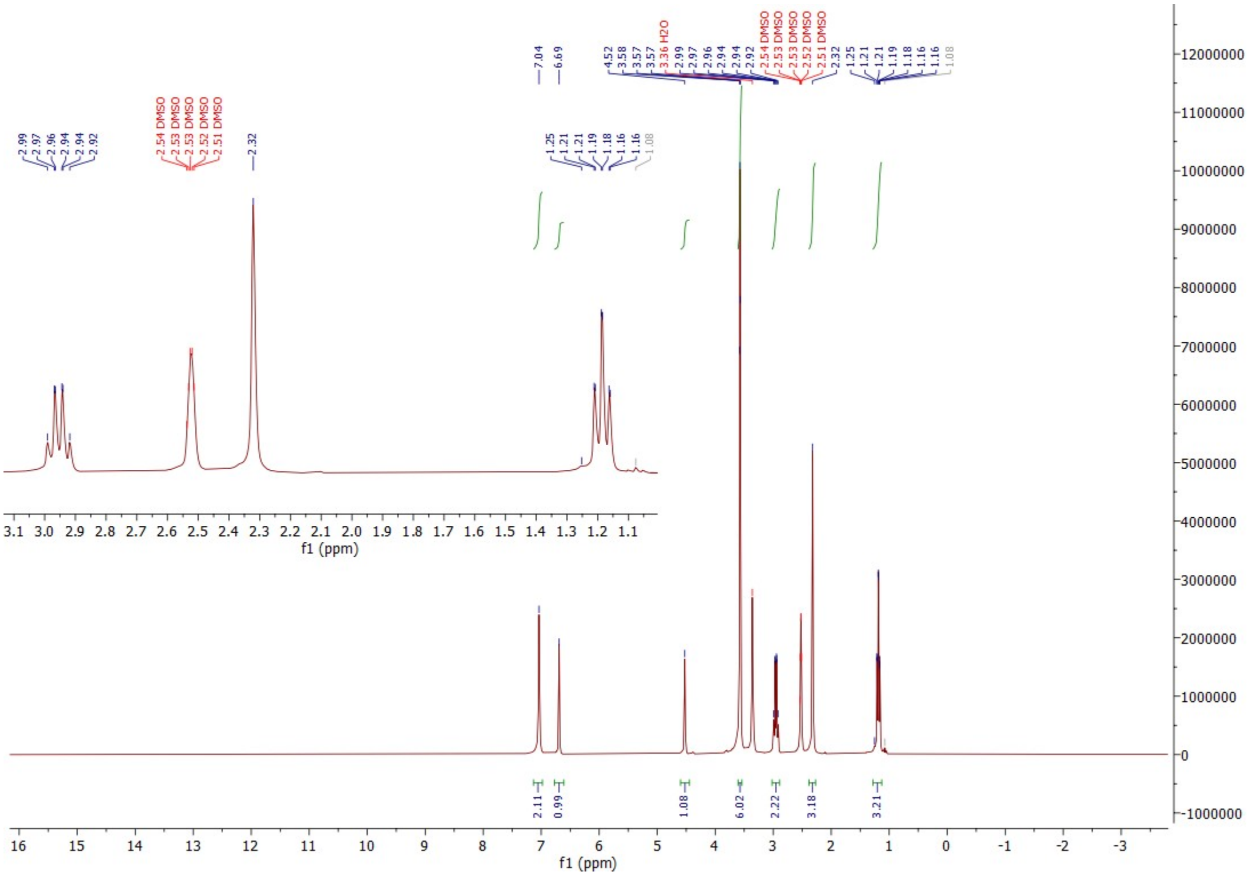

Figure S7: ^{1}HNMR spectrum of derivative **4b**

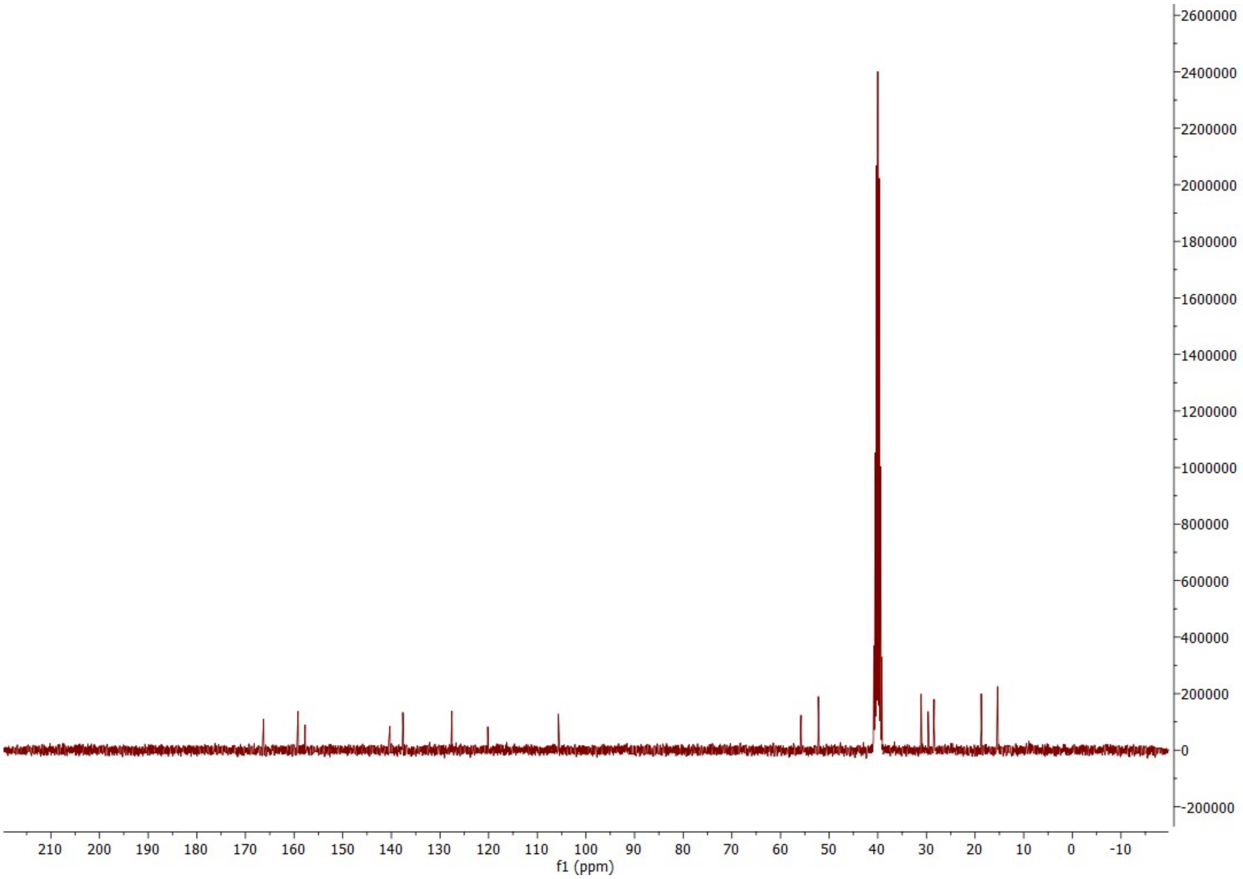

Figure S8: ^{13}CNMR spectrum of derivative **4b**

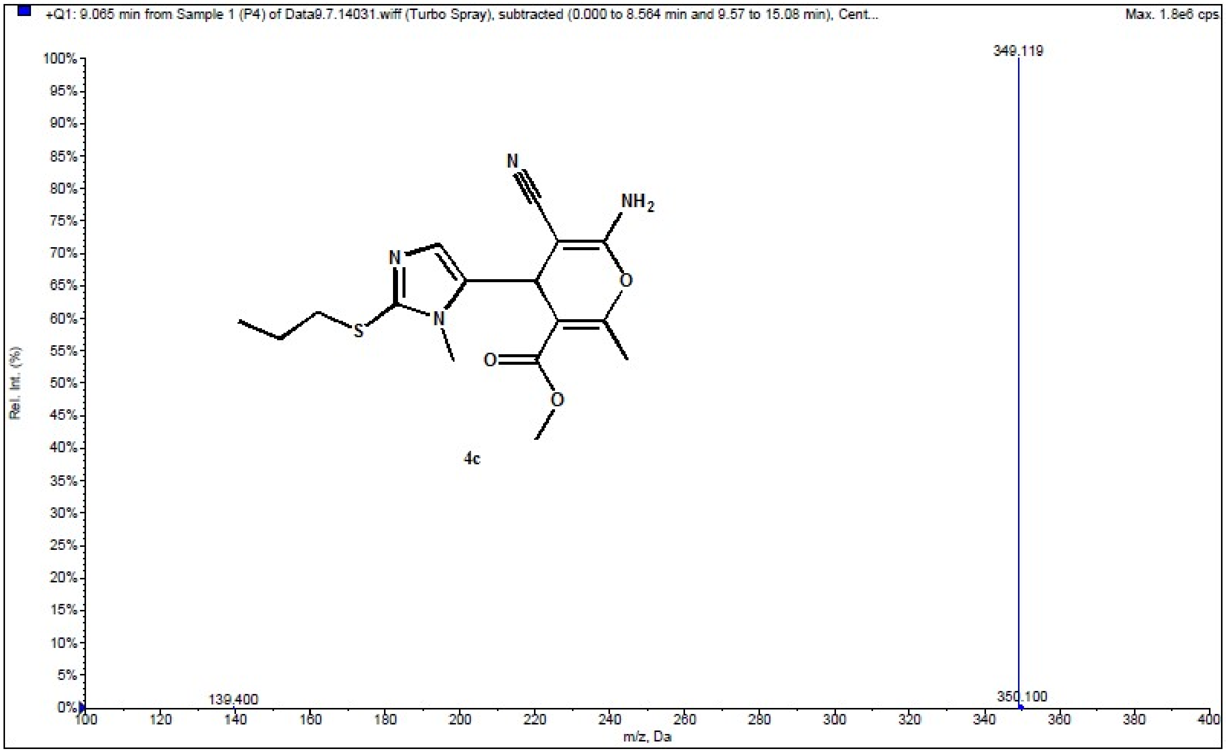

Figure S9: Mass spectrum of derivative **4c**

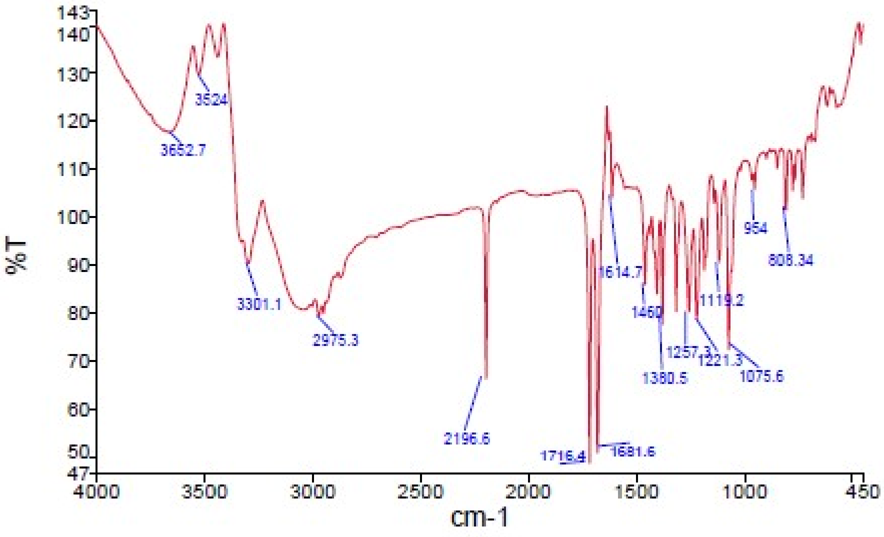

Figure S10: FTIR spectrum of derivative **4c**

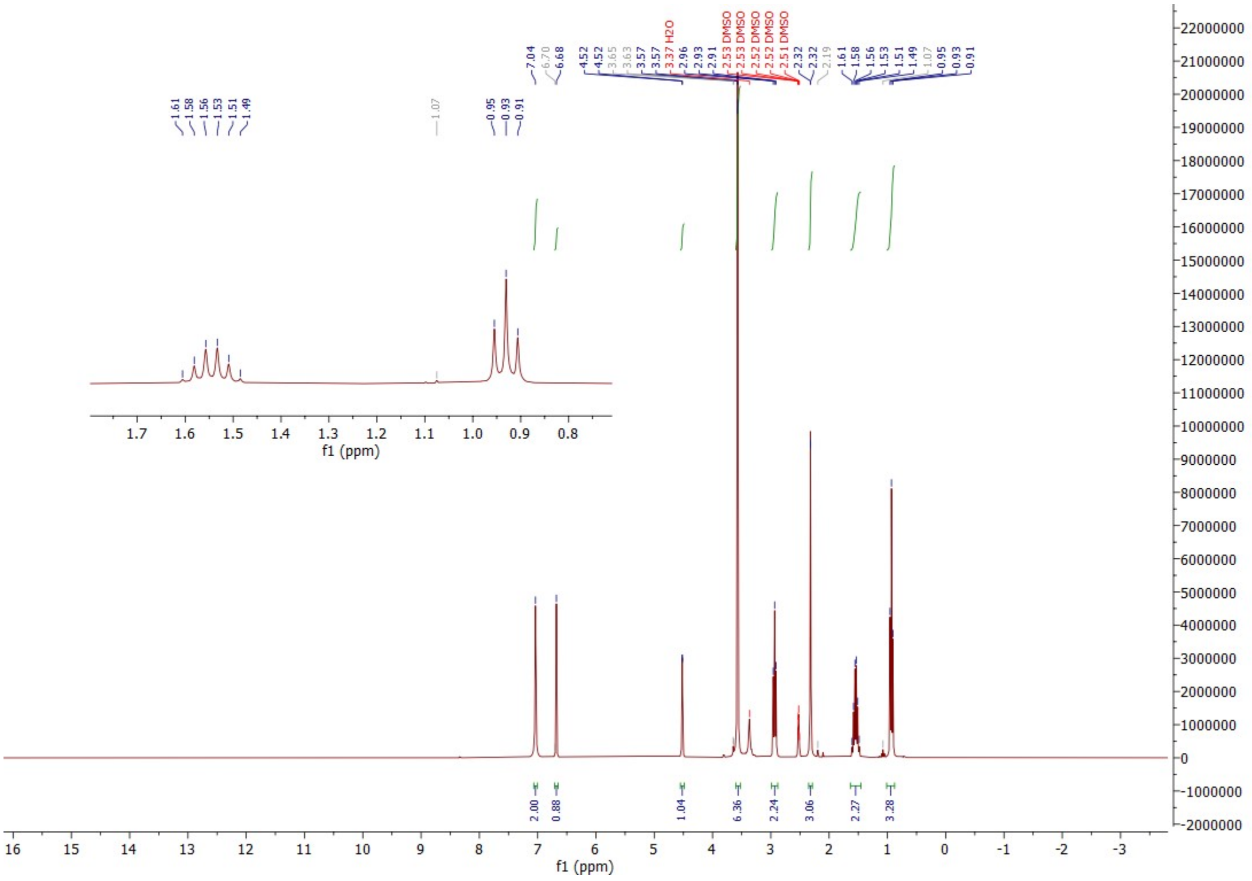

Figure S11: ^{1}HNMR spectrum of derivative **4c**

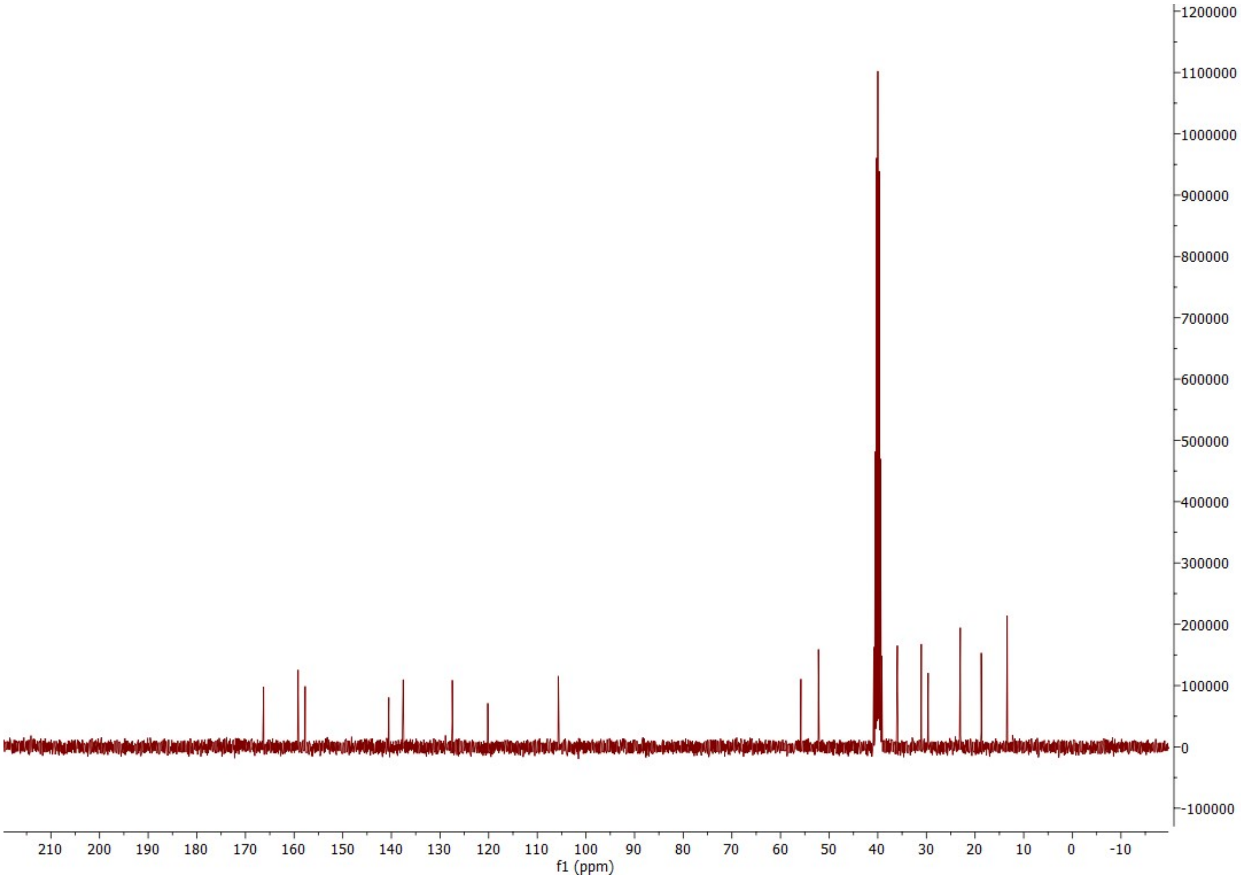

Figure S12: ^{13}CNMR spectrum of derivative **4c**

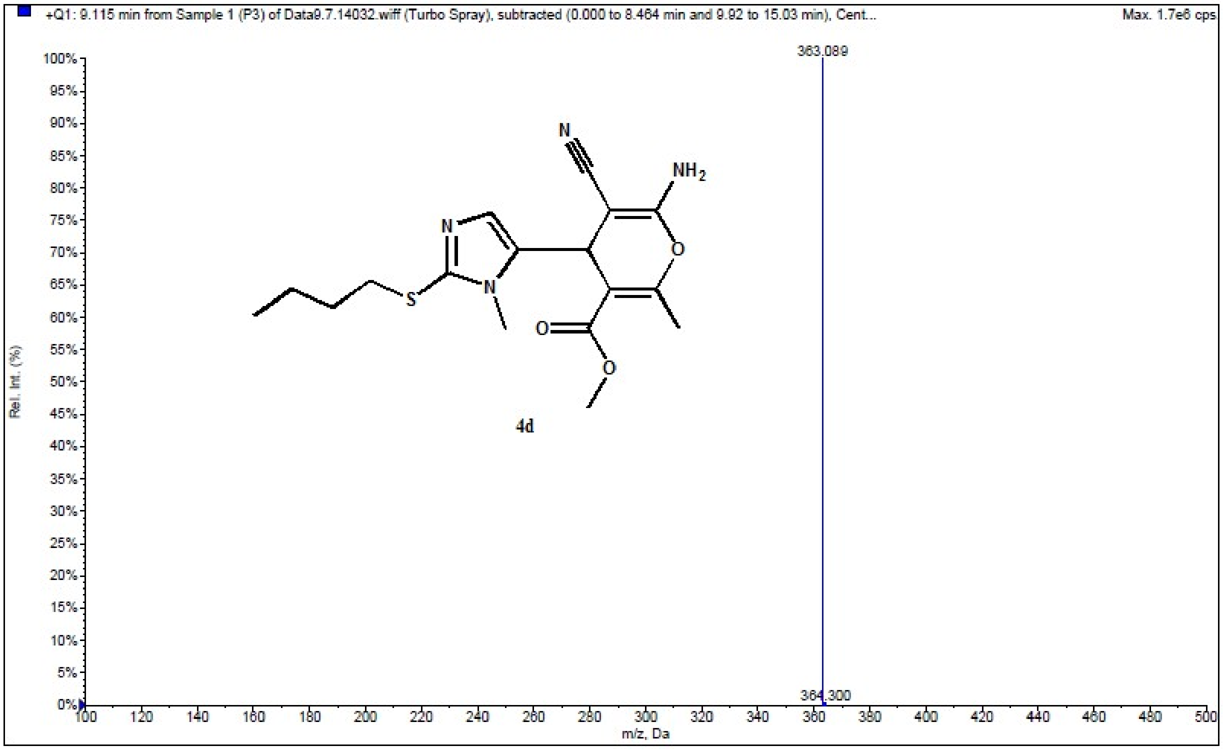

Figure S13: Mass spectrum of derivative **4d**

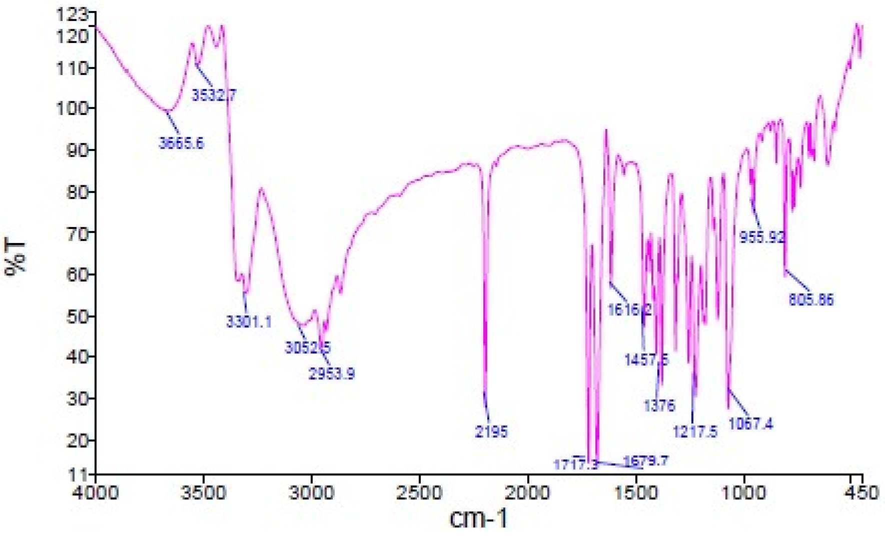

Figure S14: FTIR spectrum of derivative **4d**

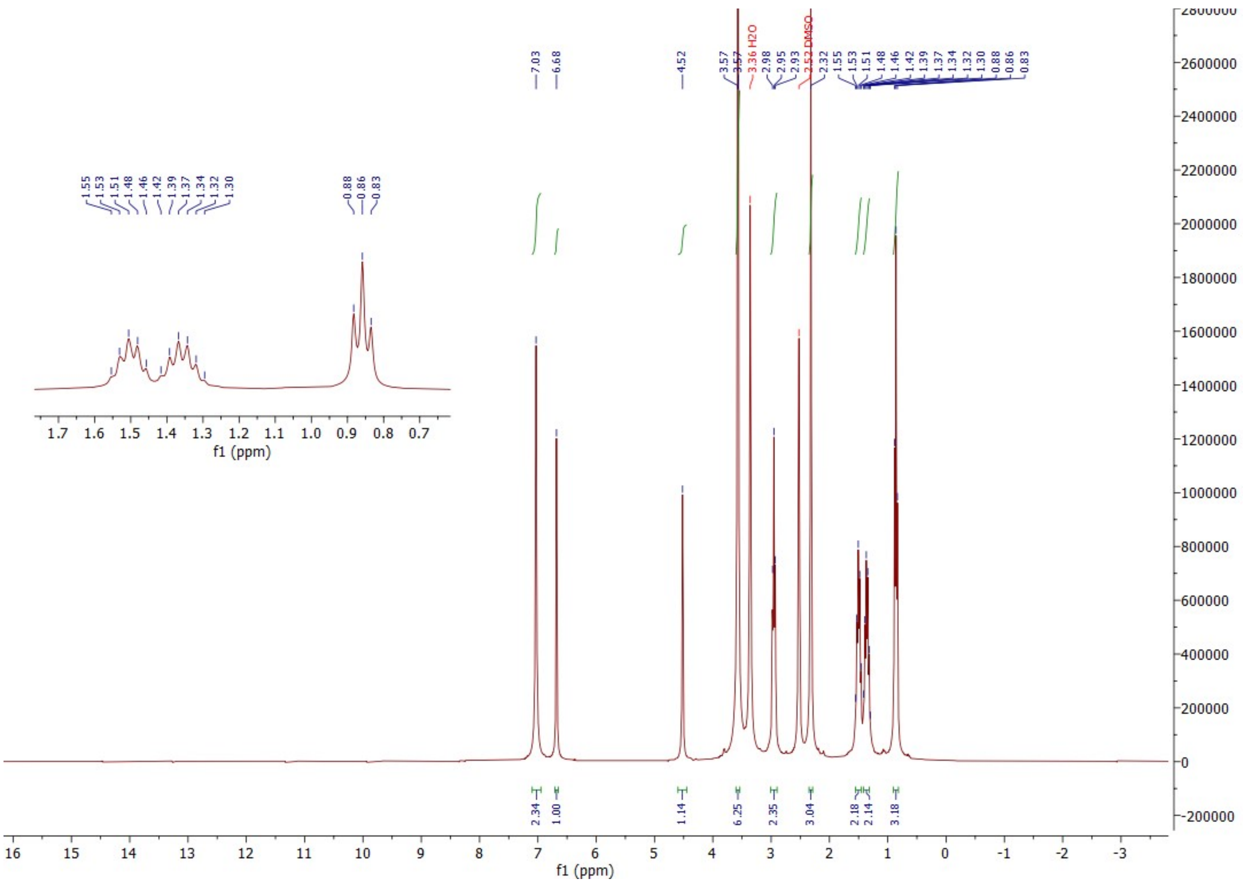

Figure S15: ^{1}HNMR spectrum of derivative **4d**

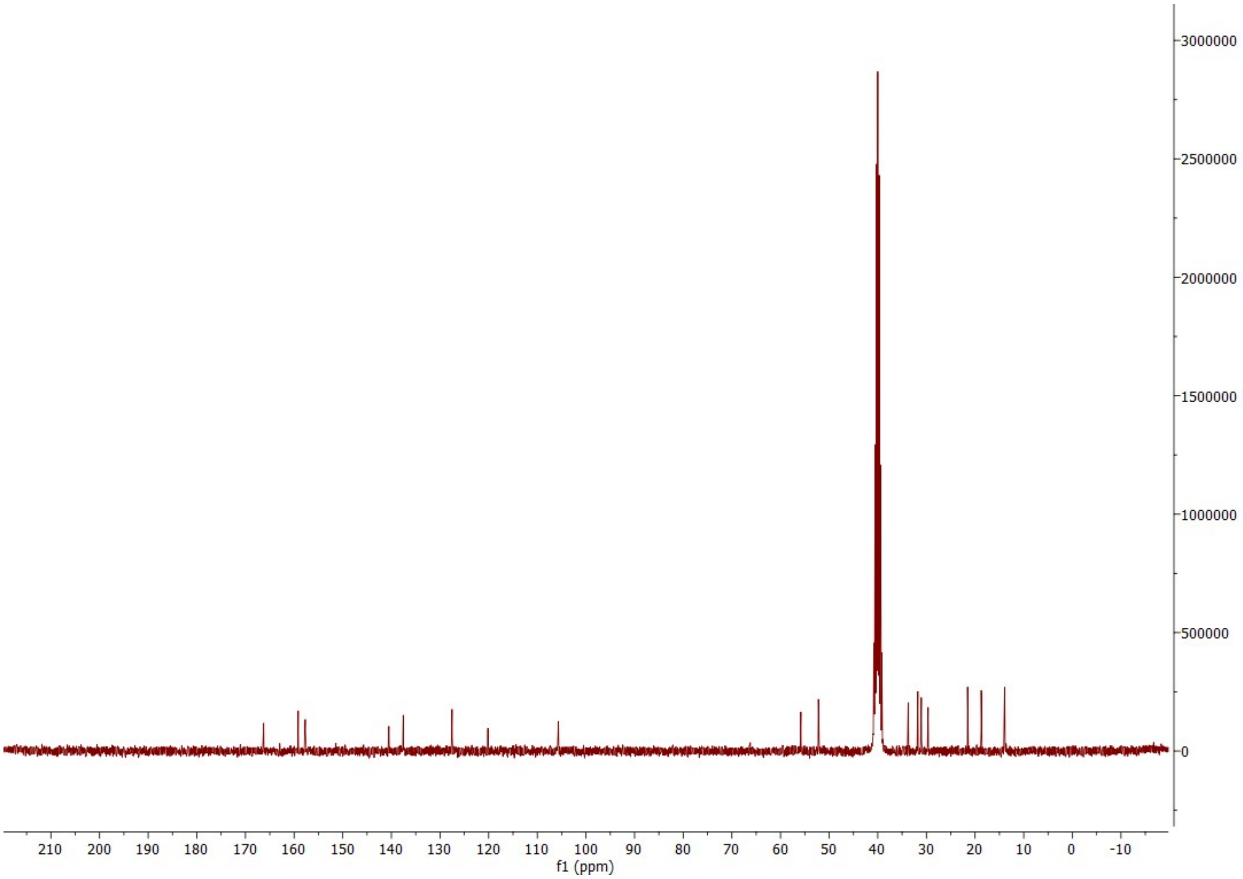

Figure S16: ^{13}CNMR spectrum of derivative **4d**

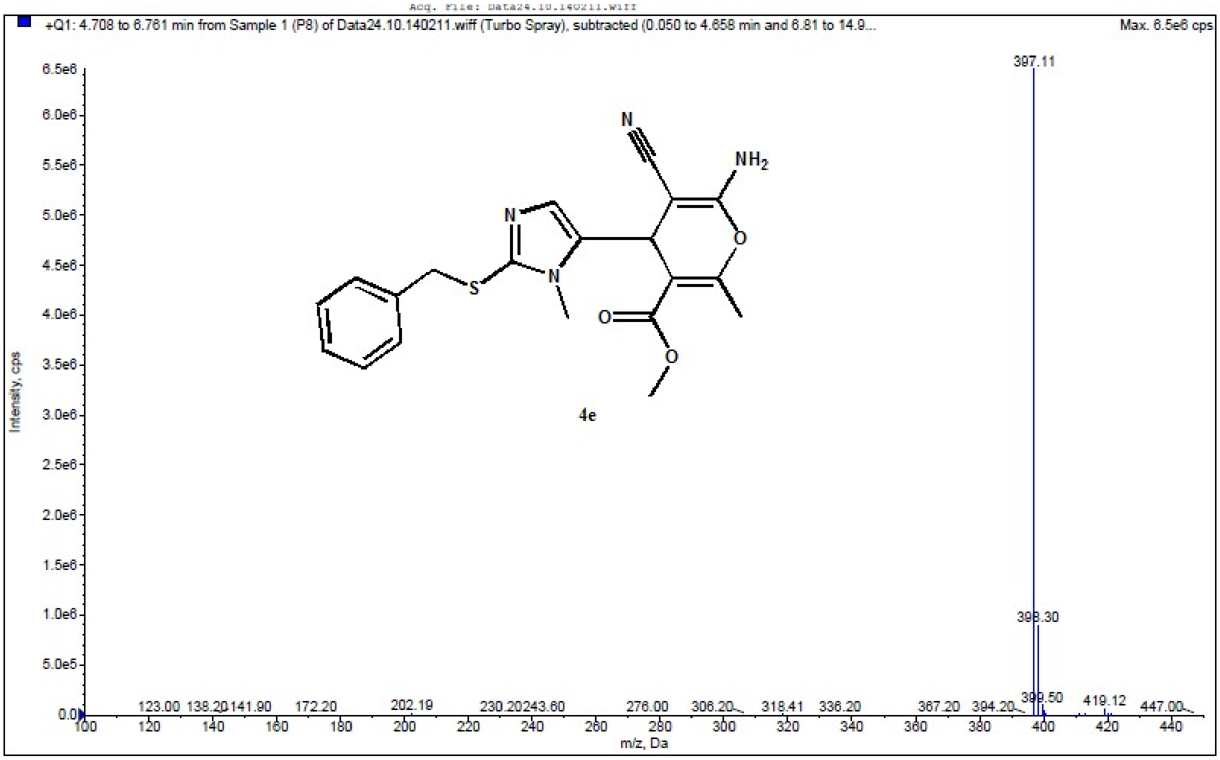

Figure S17: Mass spectrum of derivative **4e**

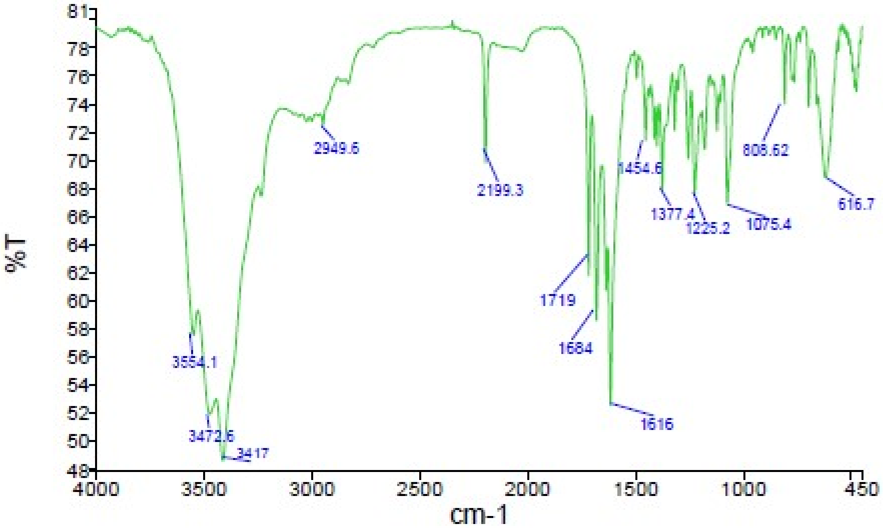

Figure S18: FTIR spectrum of derivative **4e**

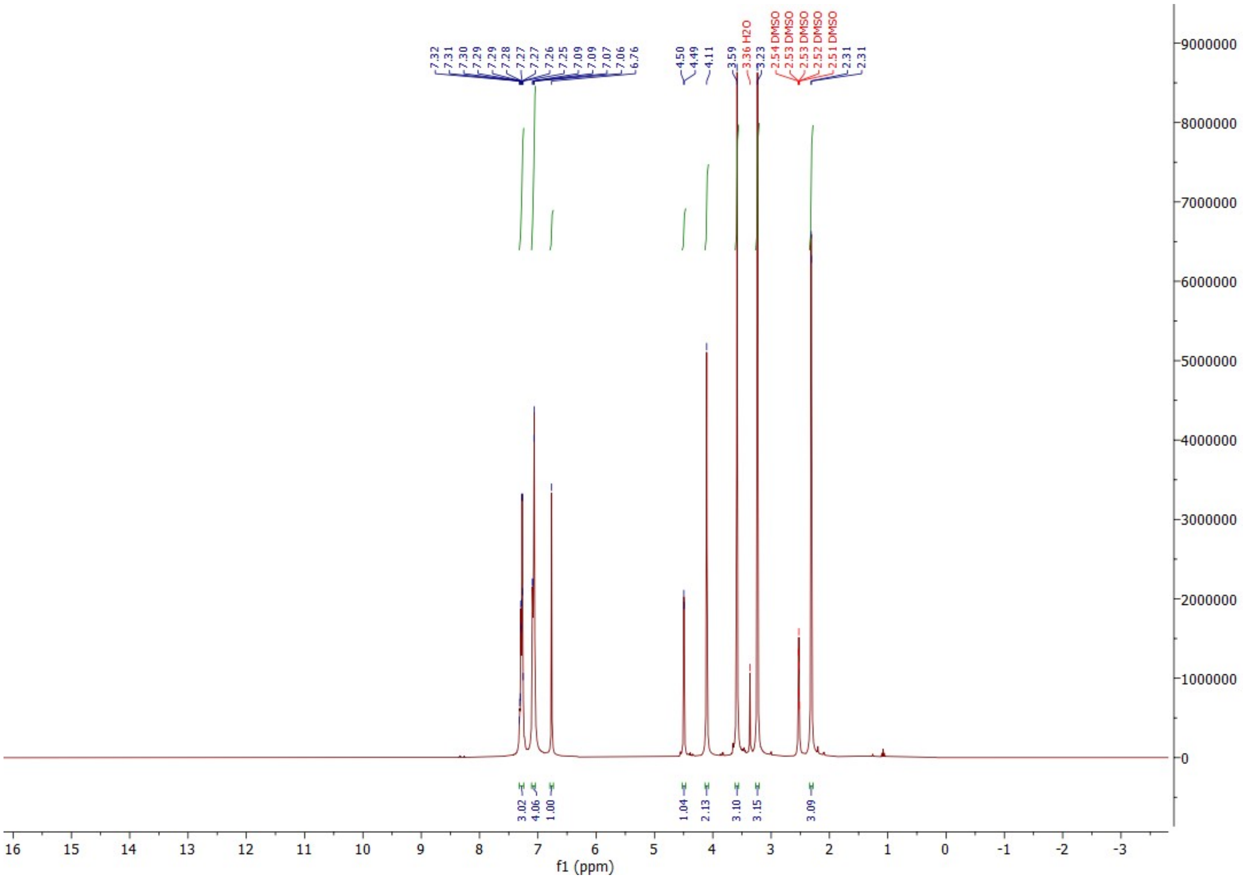

Figure S19: ^{1}HNMR spectrum of derivative **4e**

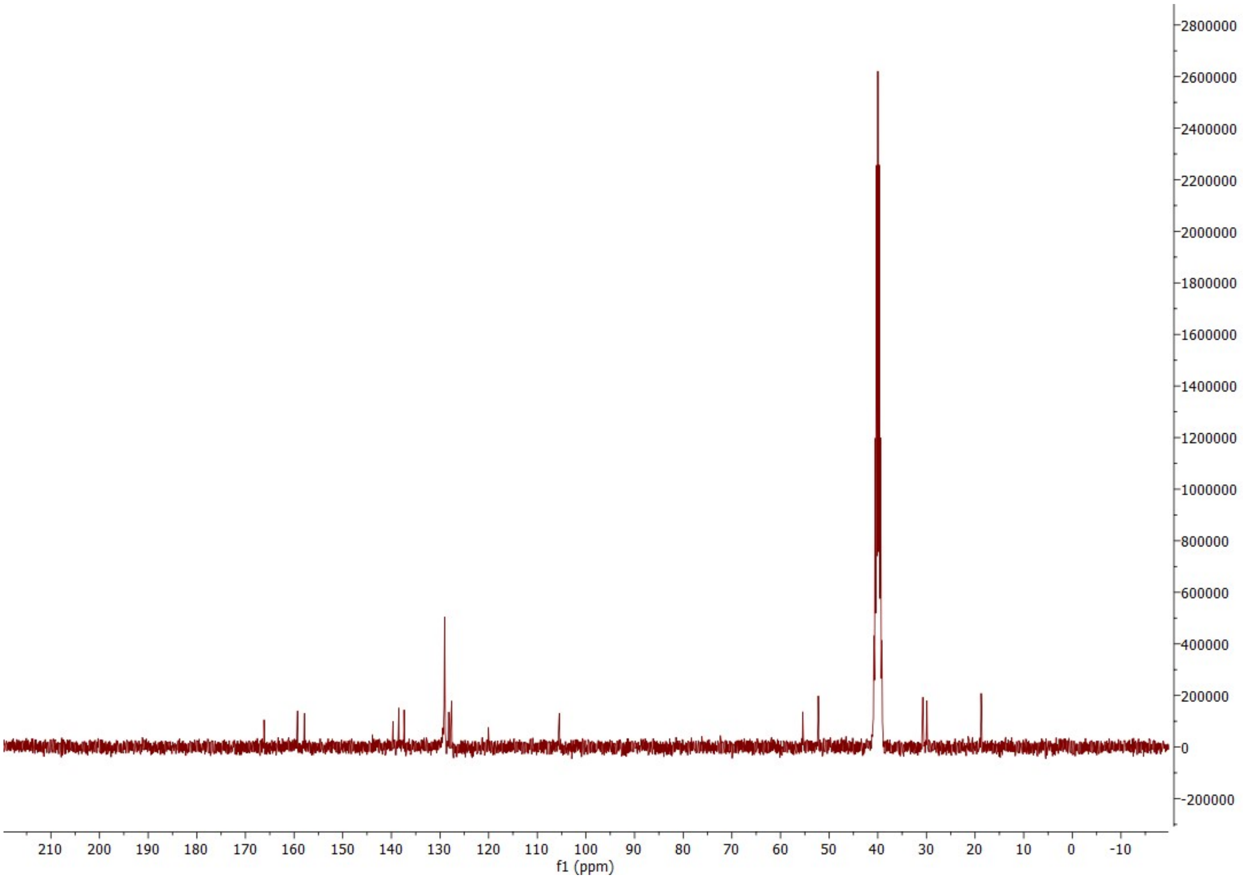

Figure S20: ^{13}CNMR spectrum of derivative **4e**

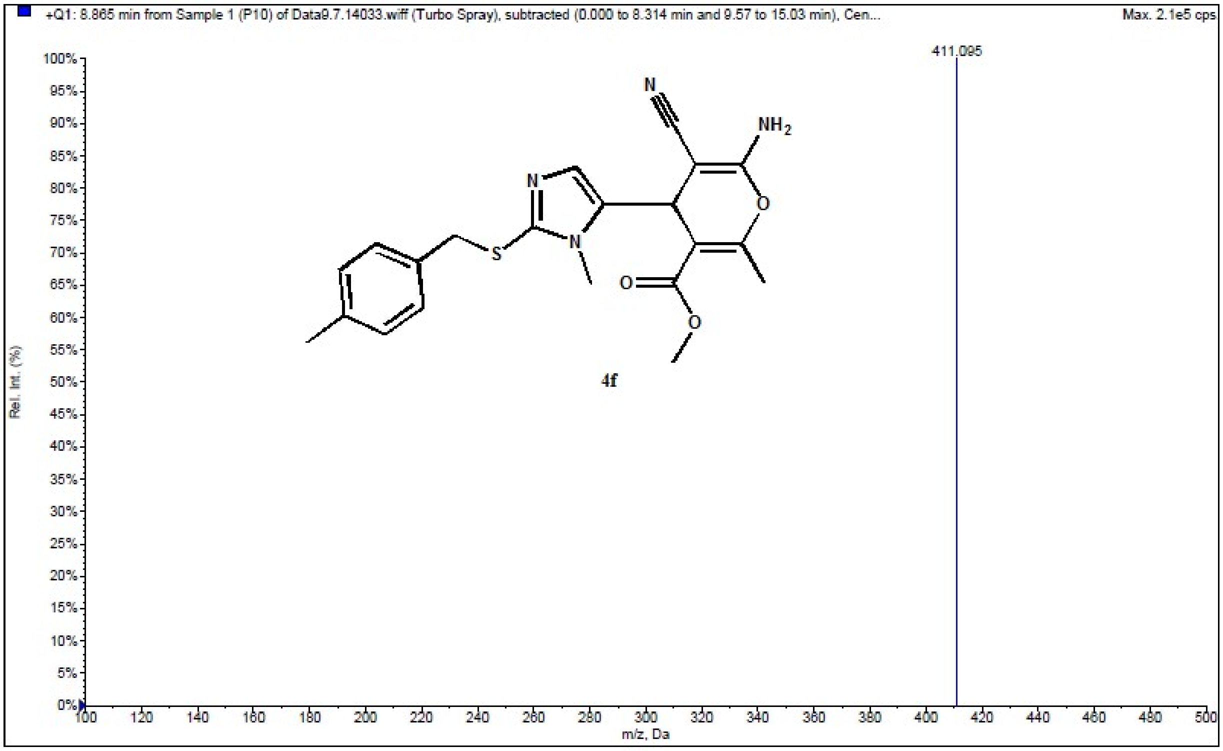

Figure S21: Mass spectrum of derivative **4f**

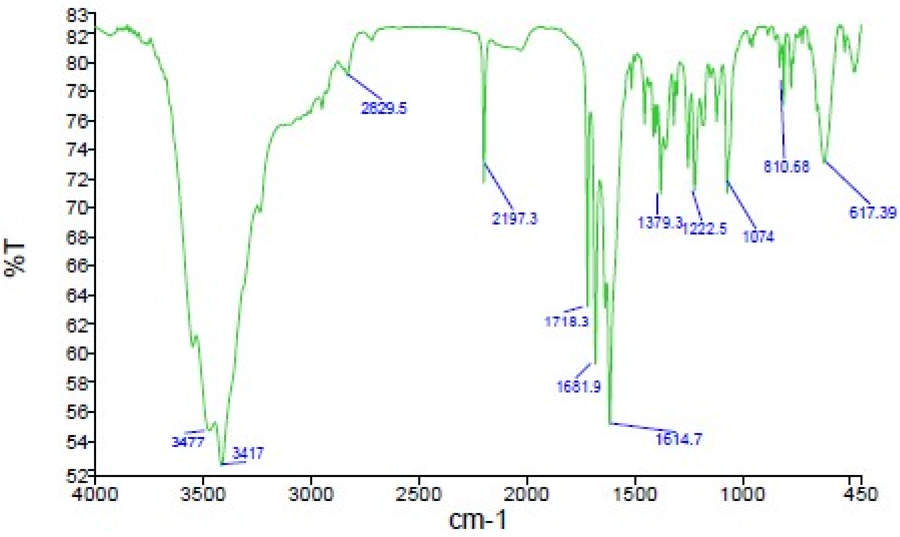

Figure S22: FTIR spectrum of derivative **4f**

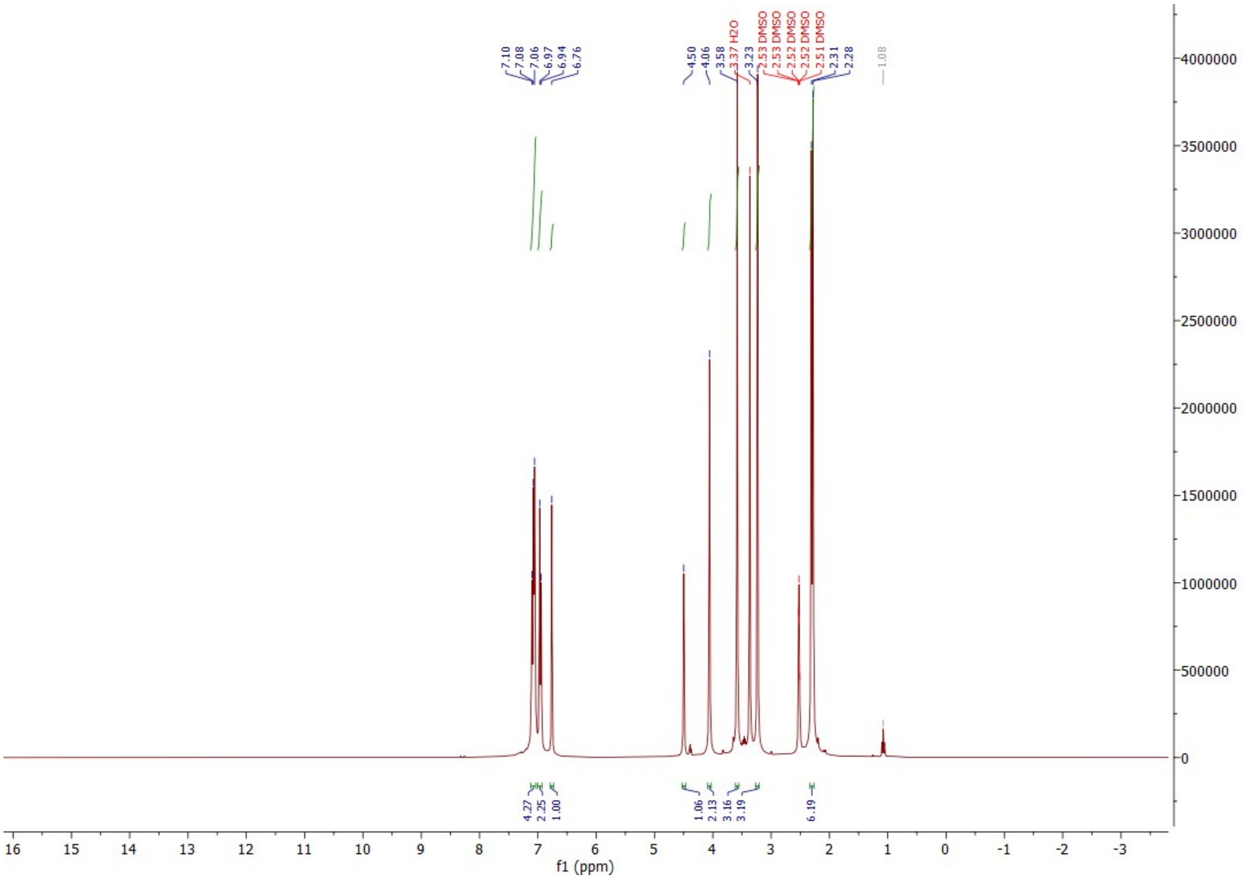

Figure S23: ^{1}HNMR spectrum of derivative **4f**

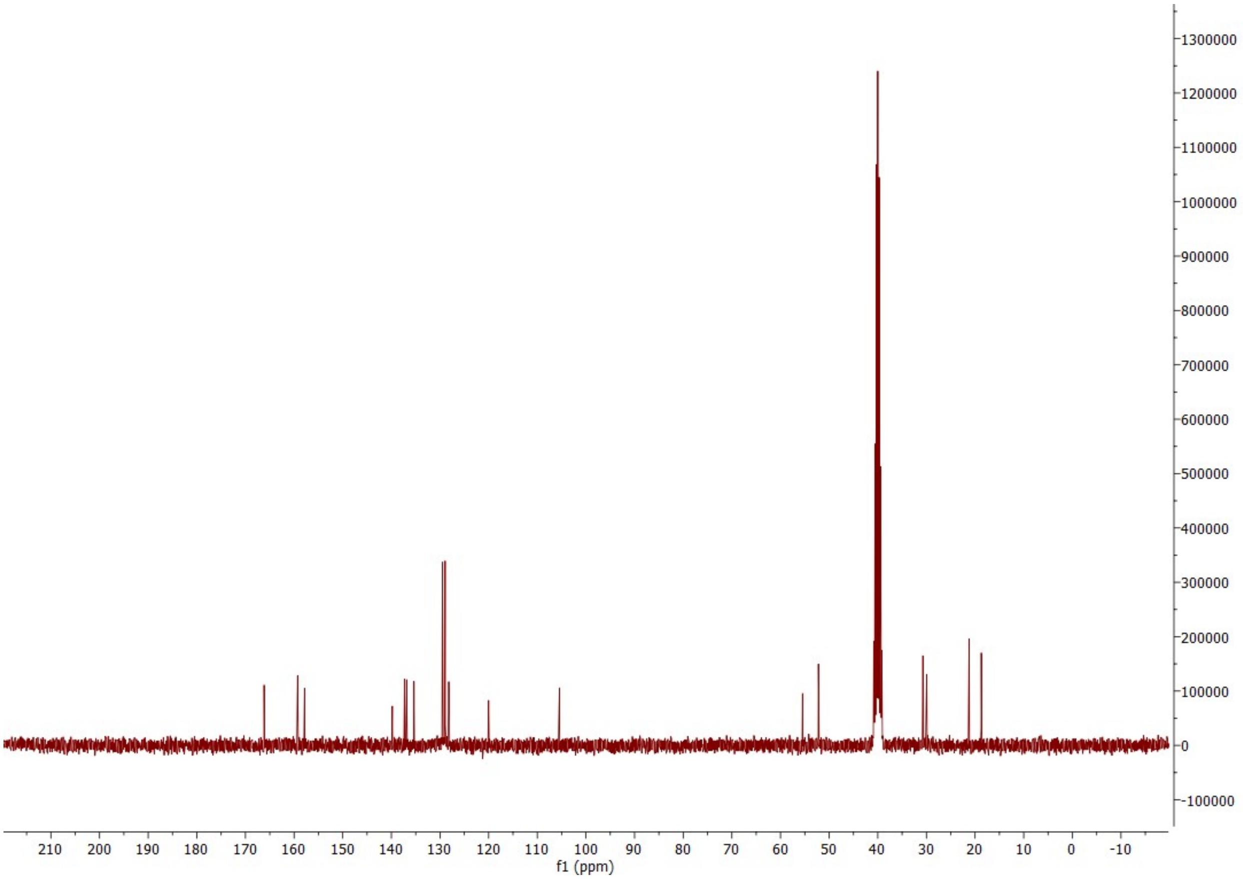

Figure S24: ^{13}CNMR spectrum of derivative **4f**

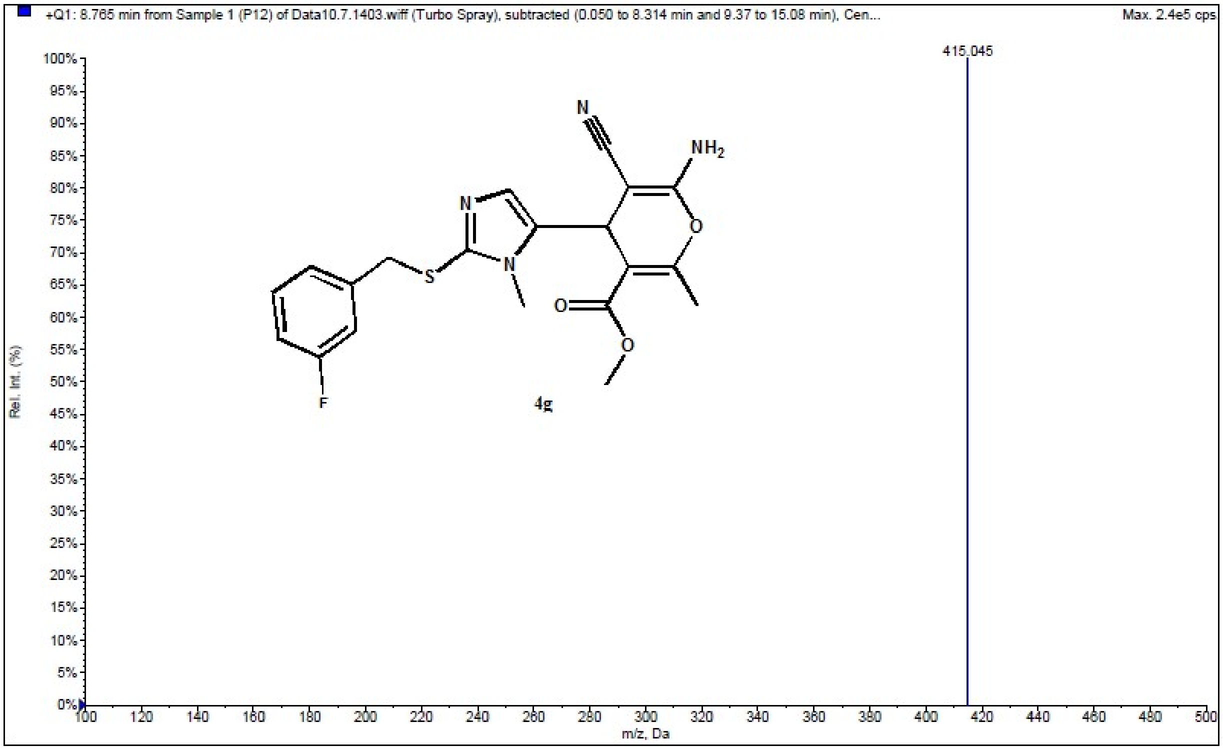

Figure S25: Mass spectrum of derivative **4g**

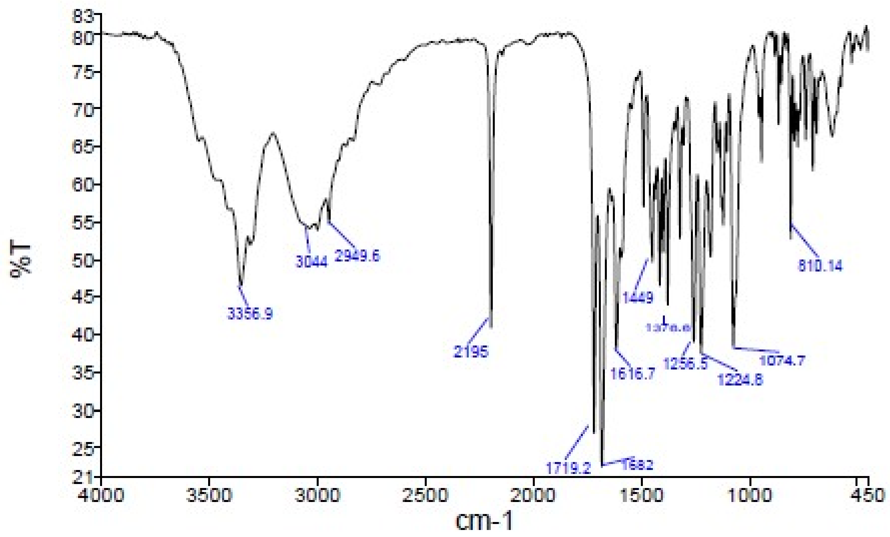

Figure S26: FTIR spectrum of derivative **4g**

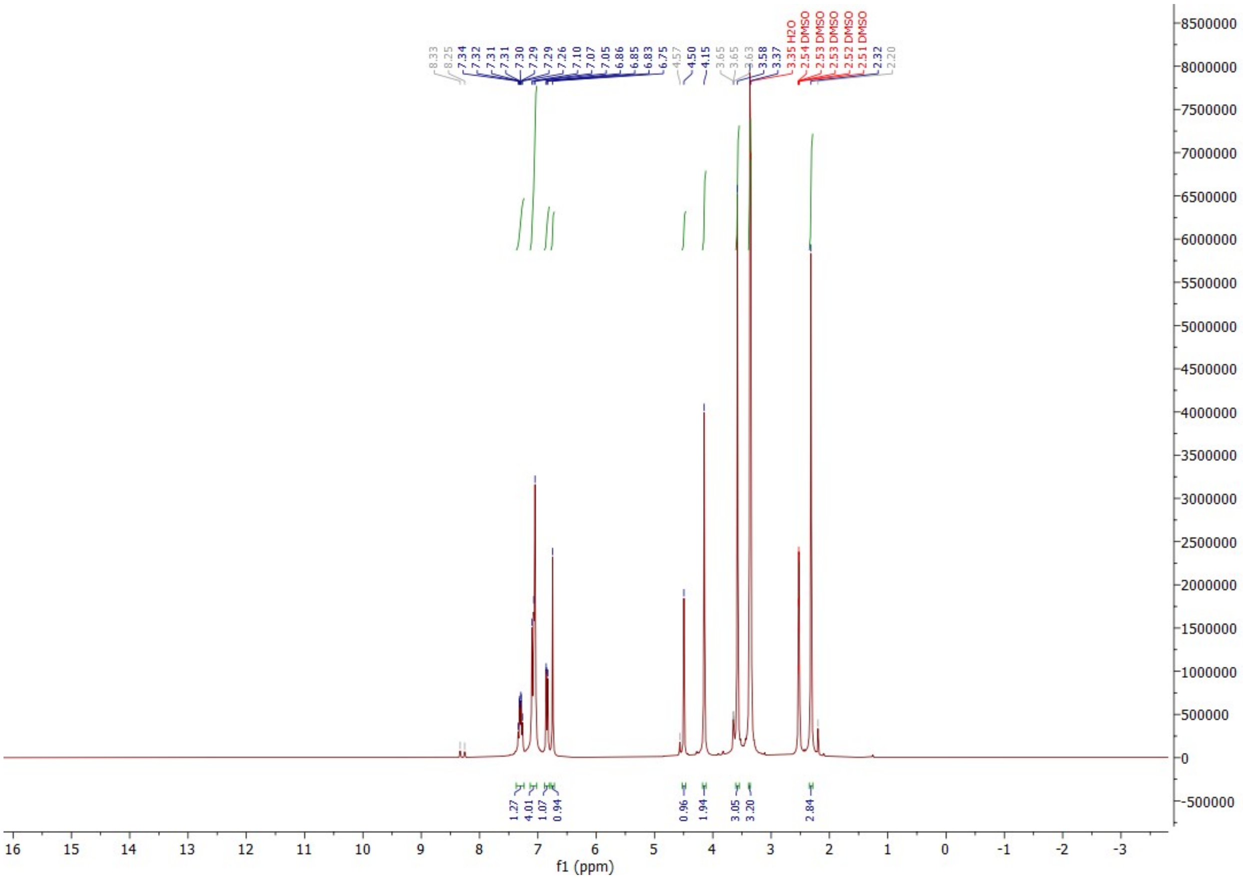

Figure S27: ^{1}HNMR spectrum of derivative **4g**

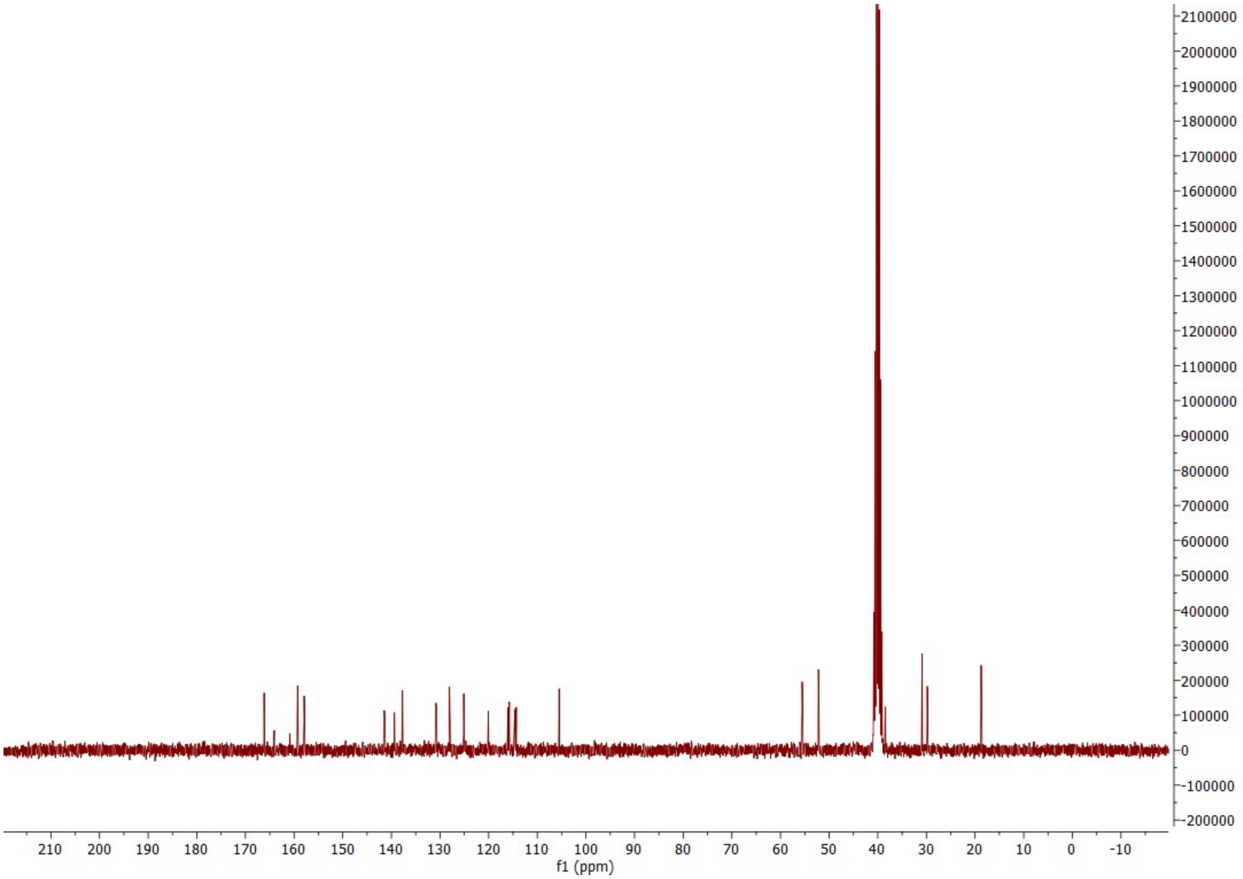

Figure S28: ^{13}CNMR spectrum of derivative **4g**

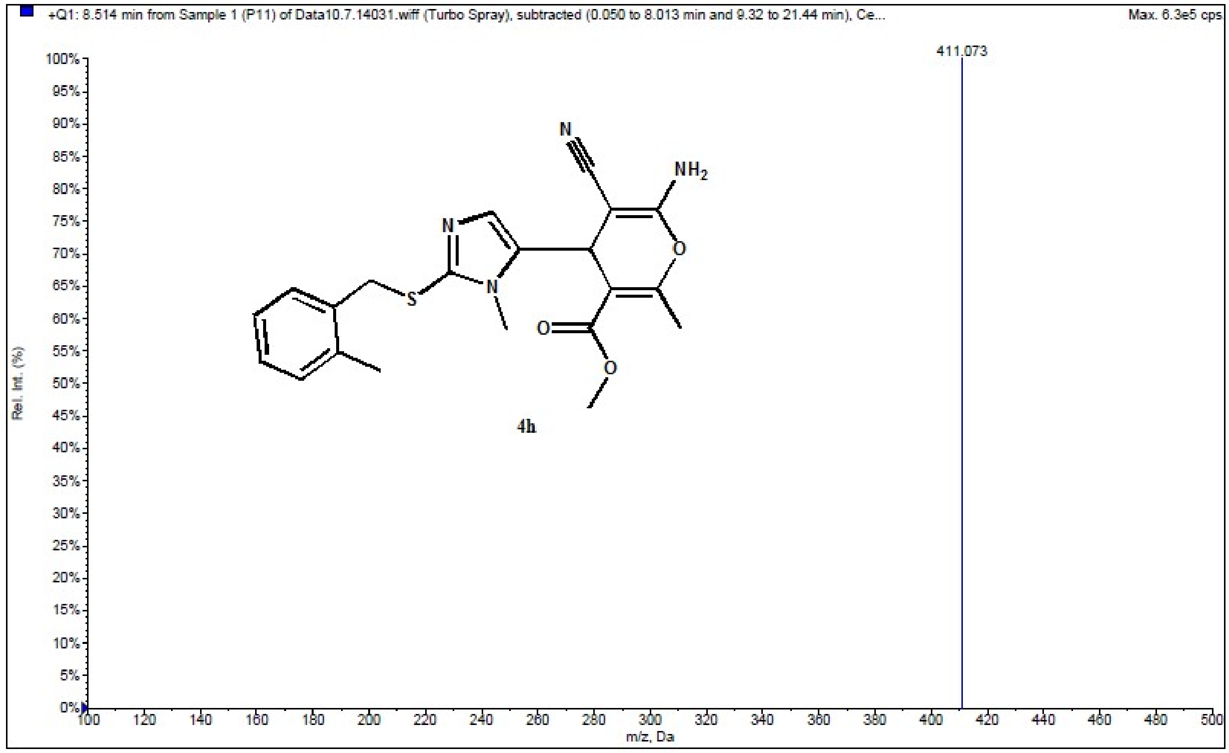

Figure S29: Mass spectrum of derivative **4h**

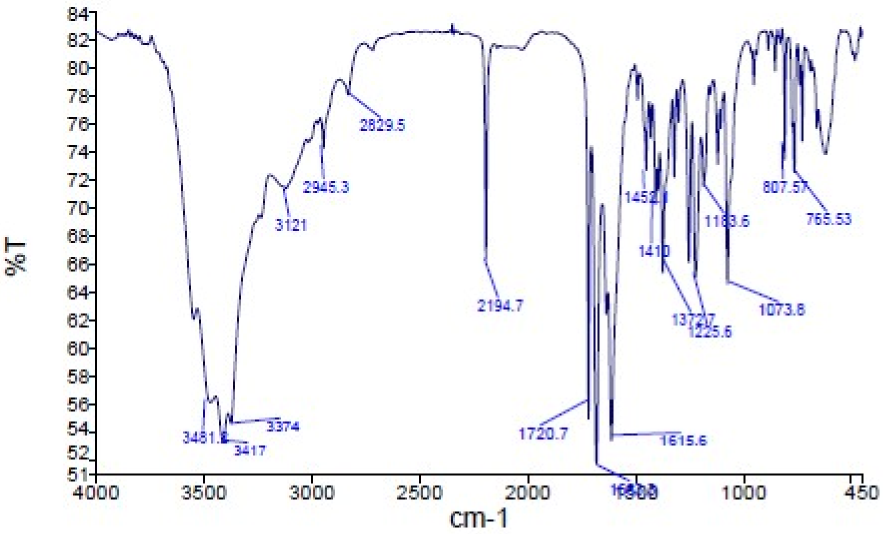

Figure S30: FTIR spectrum of derivative **4h**

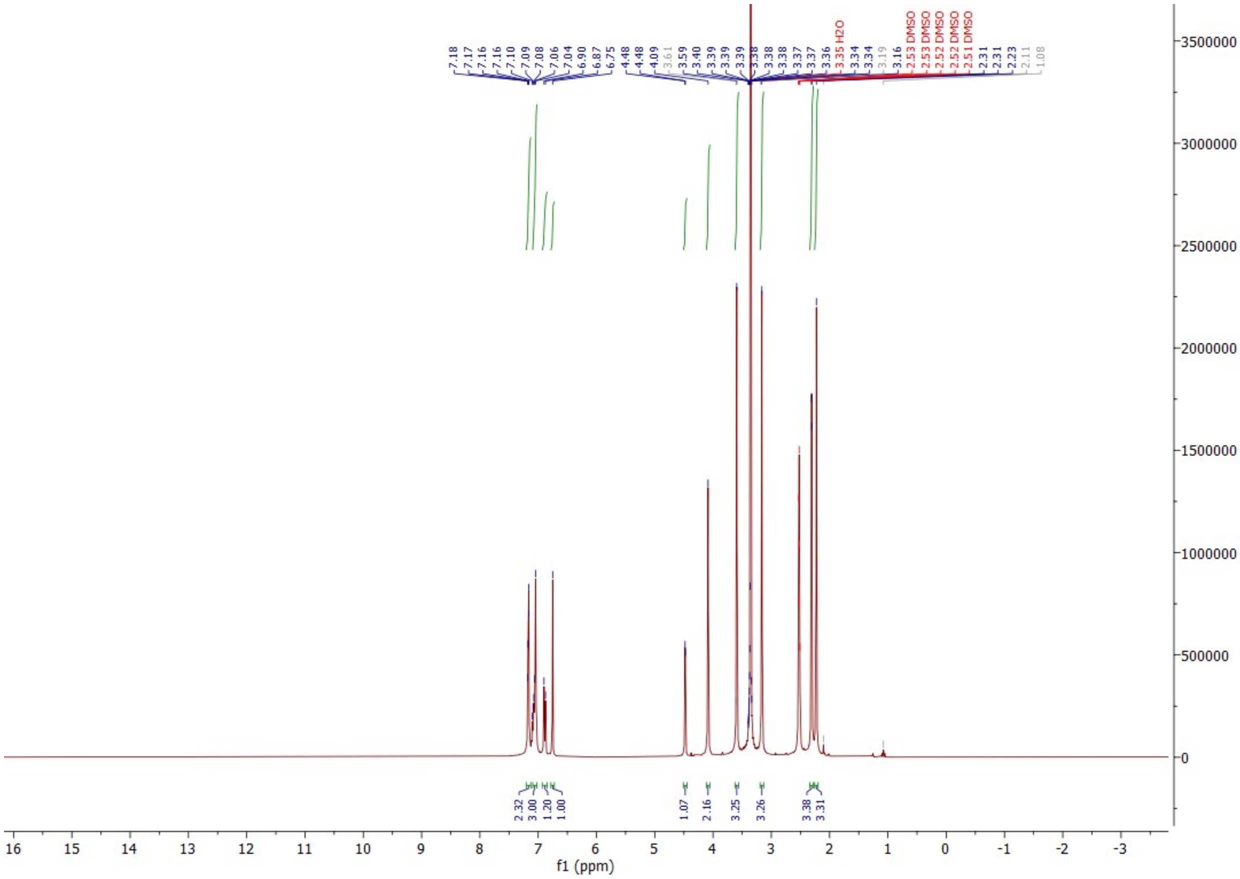

Figure S31: ^{1}HNMR spectrum of derivative **4h**

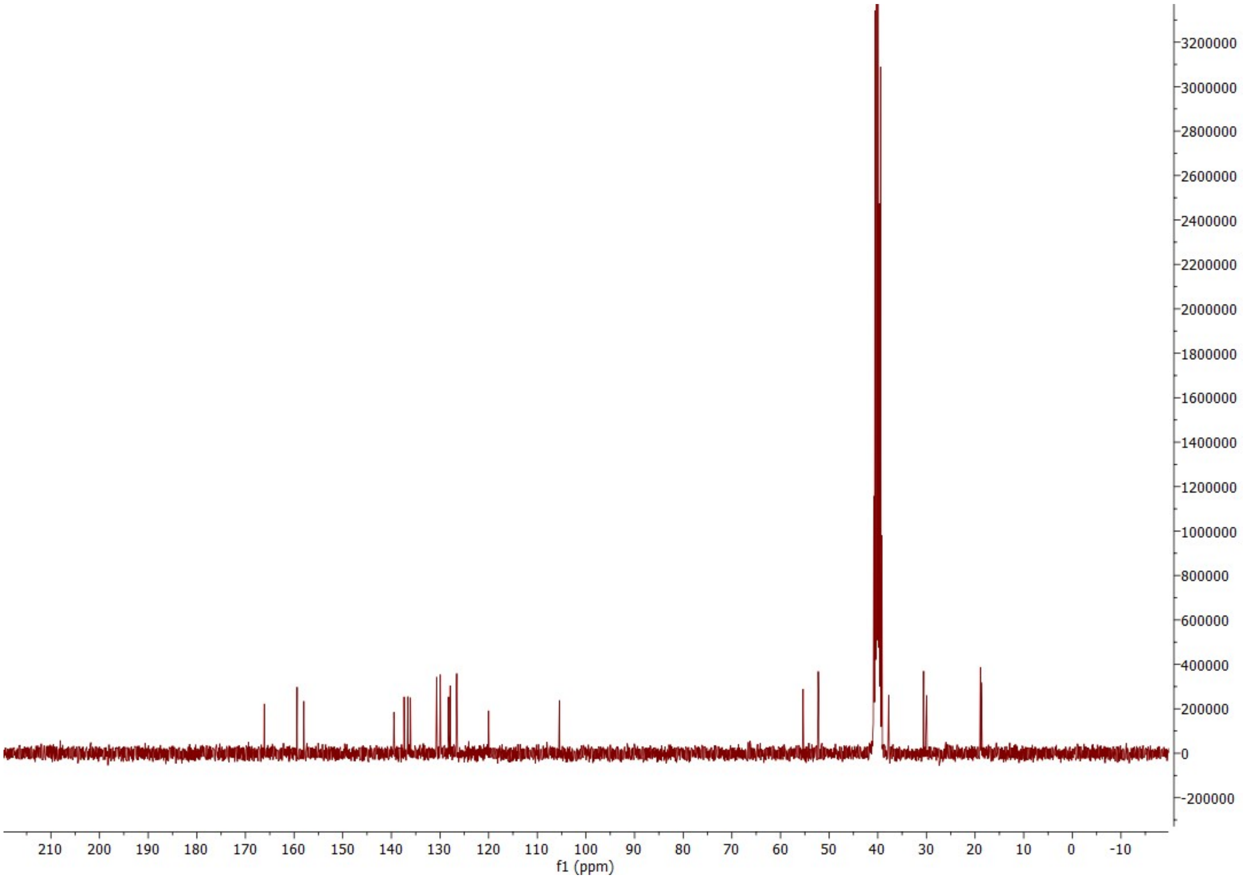

Figure S32: ^{13}CNMR spectrum of derivative **4h**

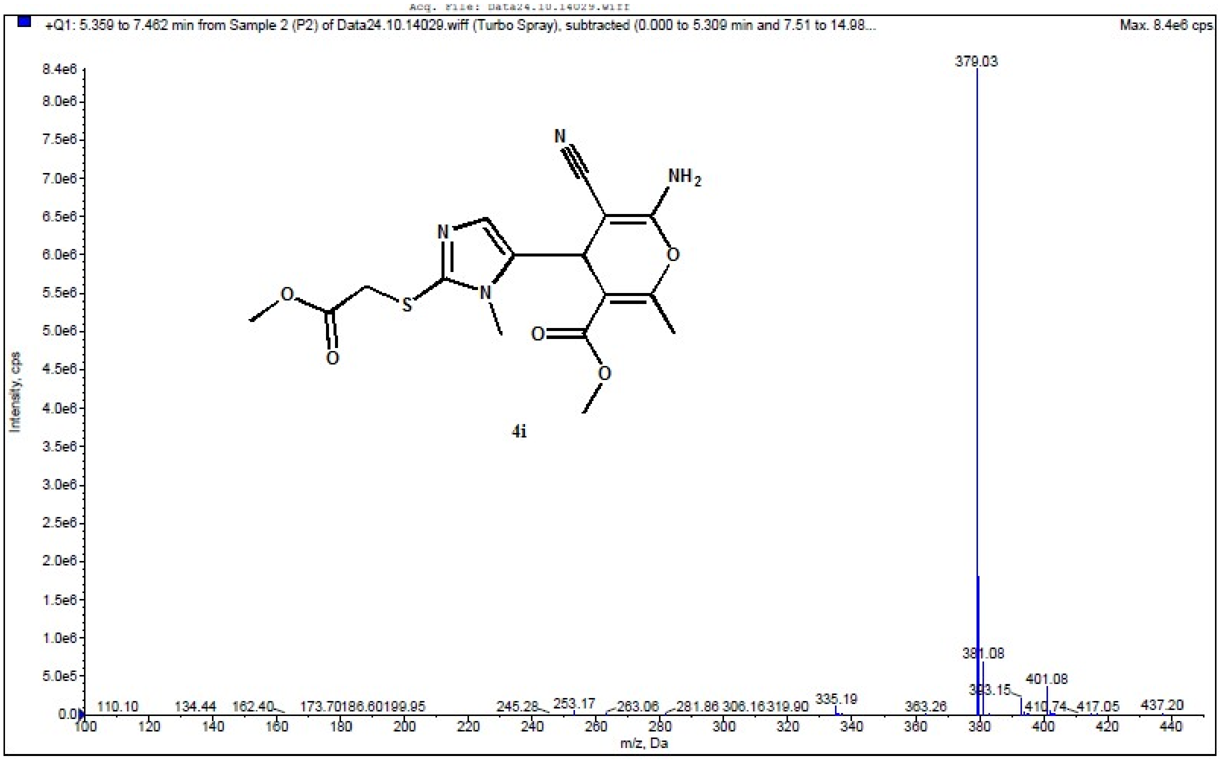

Figure S33: Mass spectrum of derivative **4i**

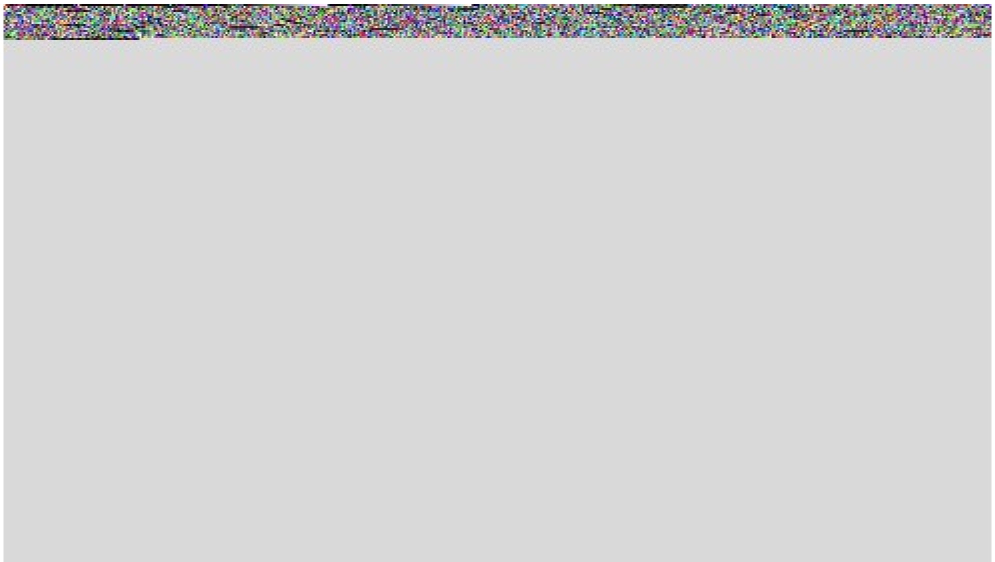

Figure S34: FTIR spectrum of derivative **4i**

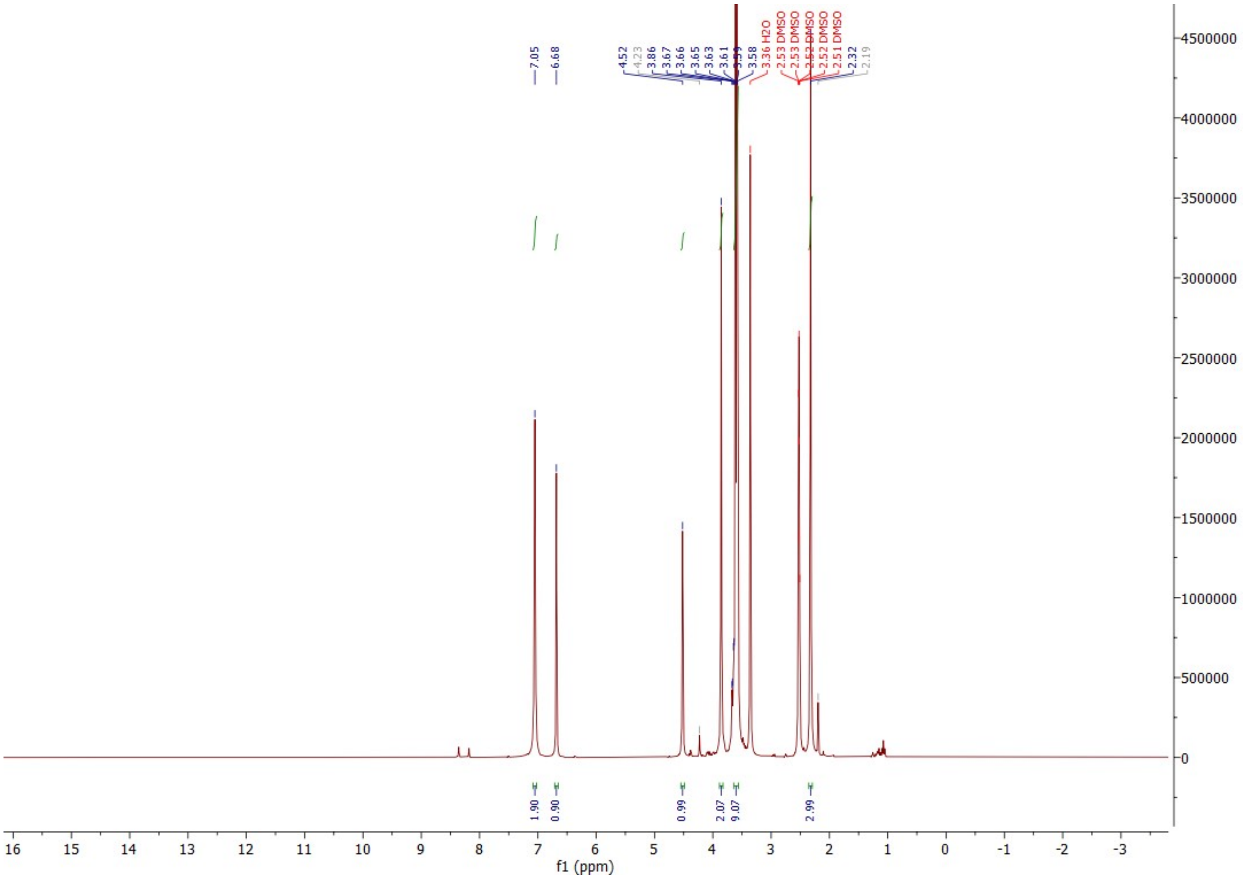

Figure S35: ^{1}HNMR spectrum of derivative **4i**

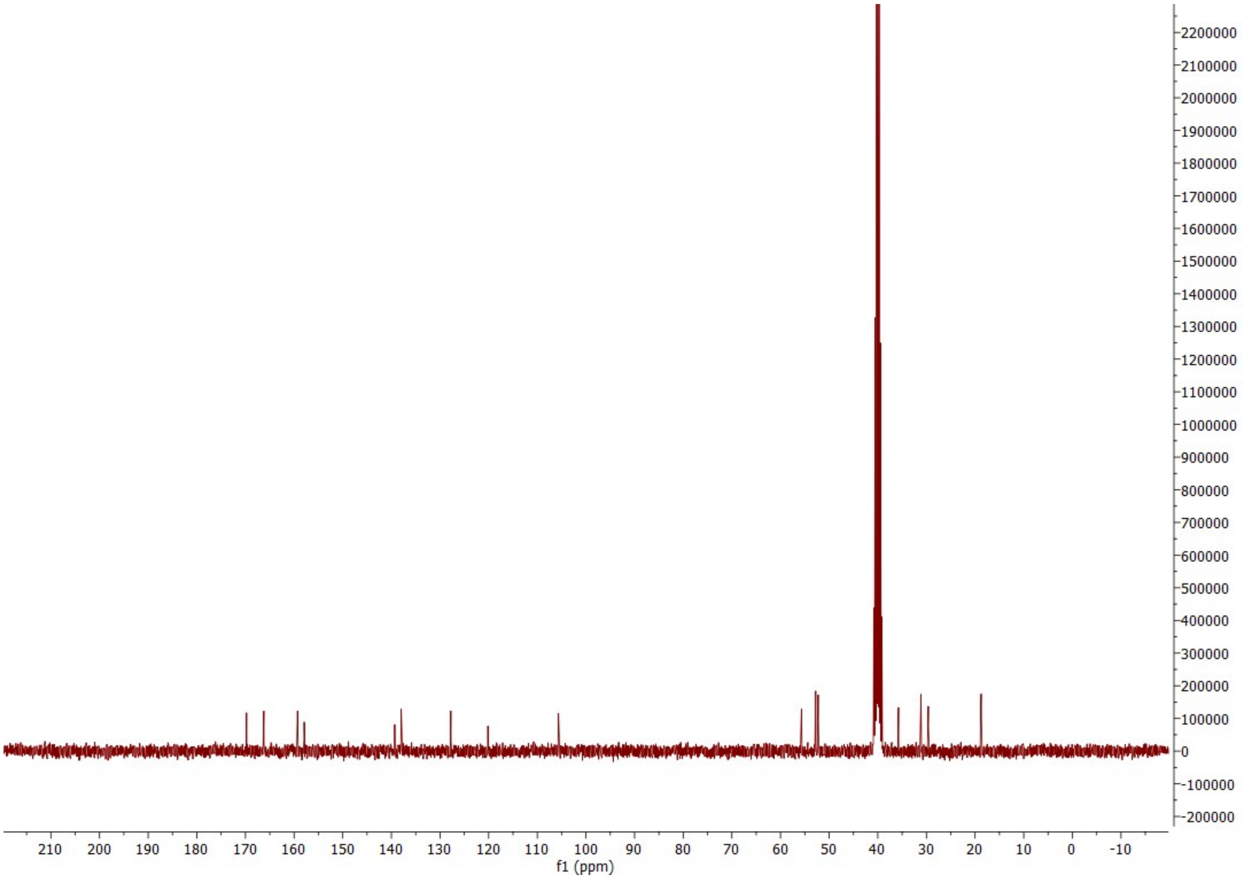

Figure S36: ^{13}CNMR spectrum of derivative **4i**

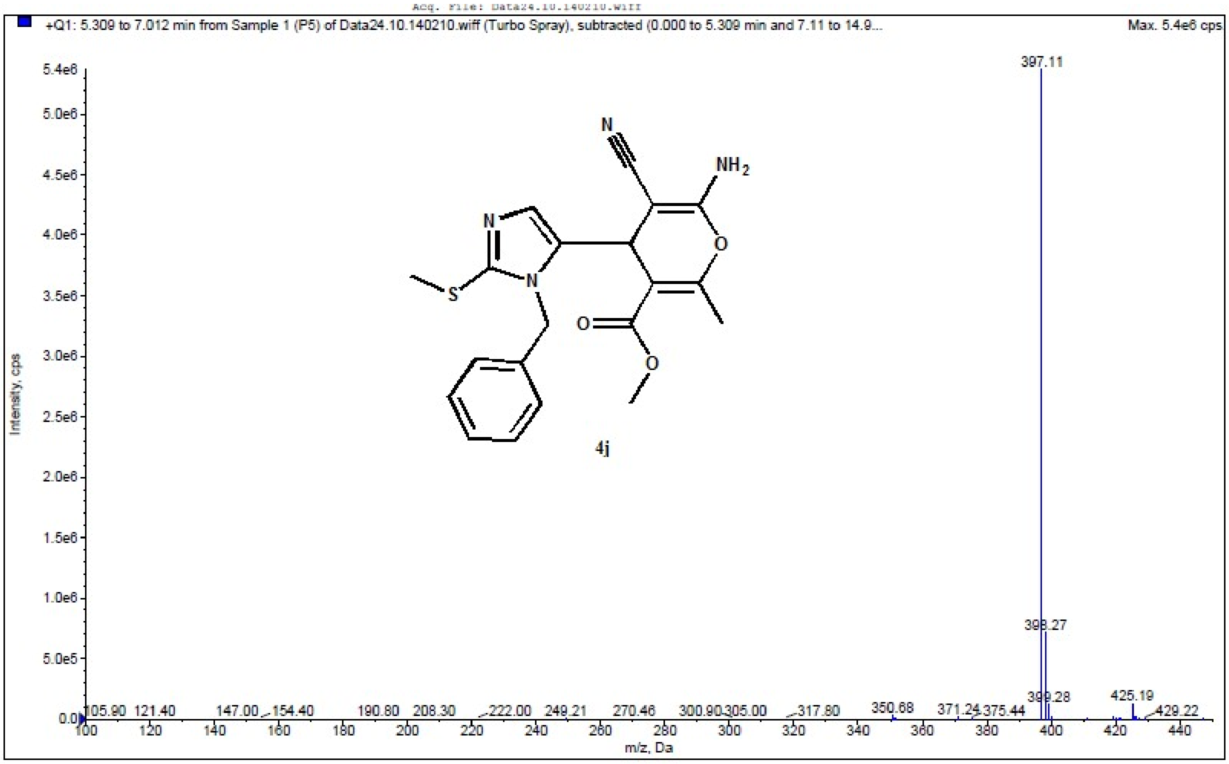

Figure S37: Mass spectrum of derivative **4j**

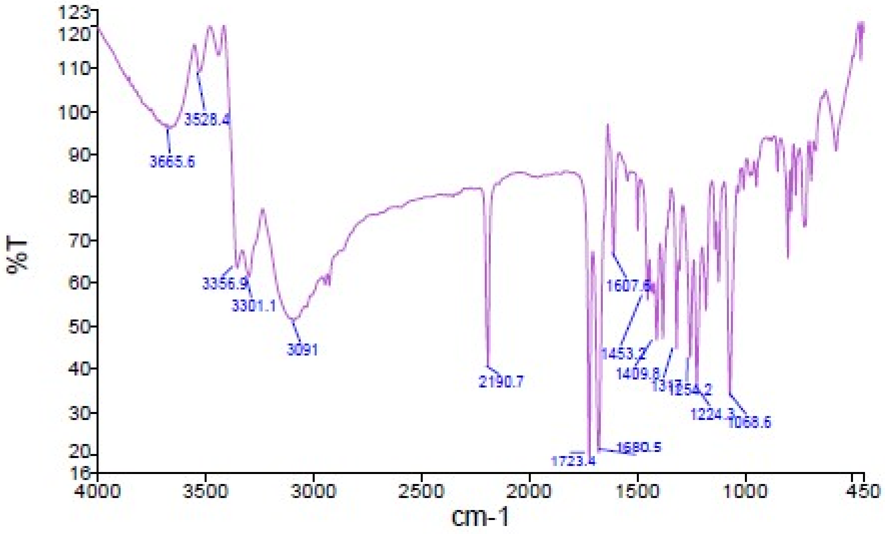

Figure S38: FTIR spectrum of derivative **4j**

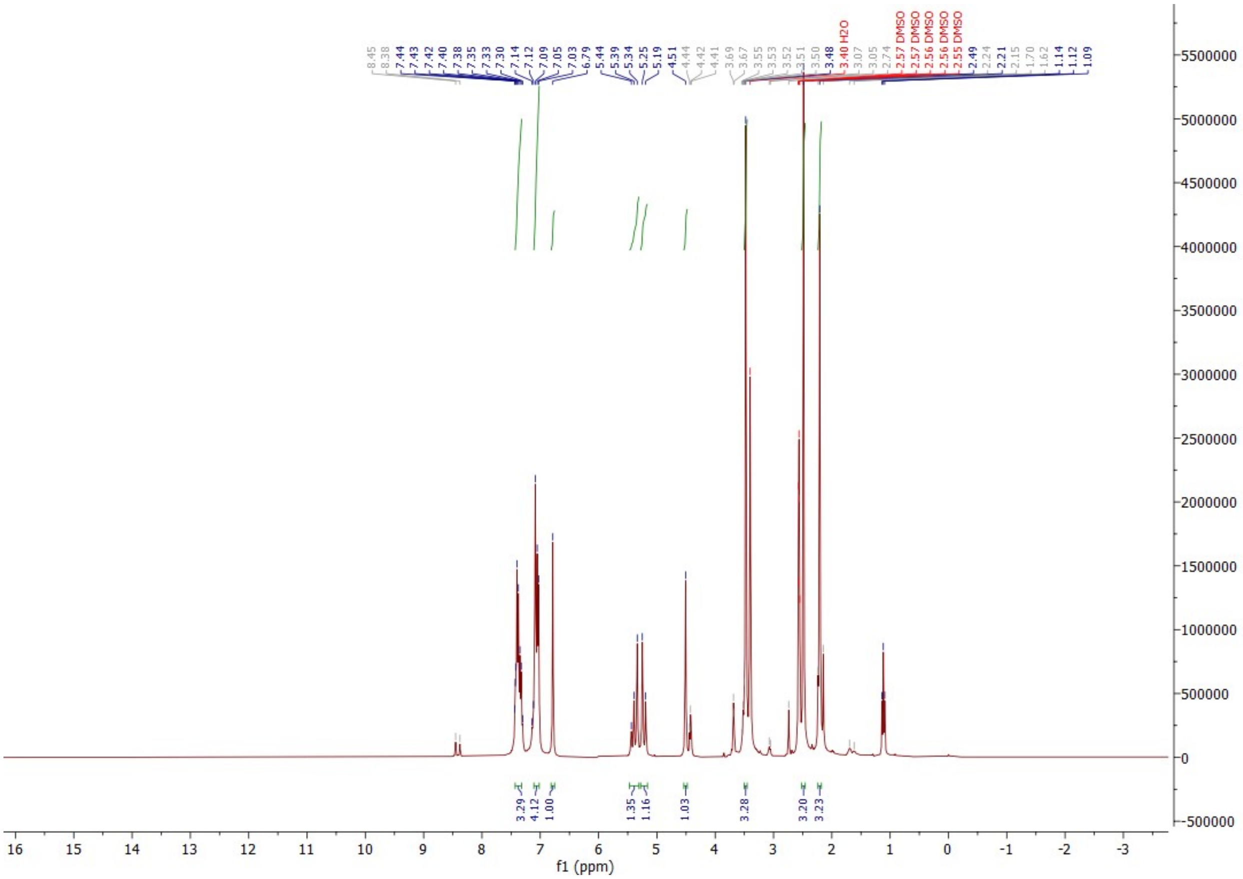

Figure S39: ^{1}HNMR spectrum of derivative **4j**

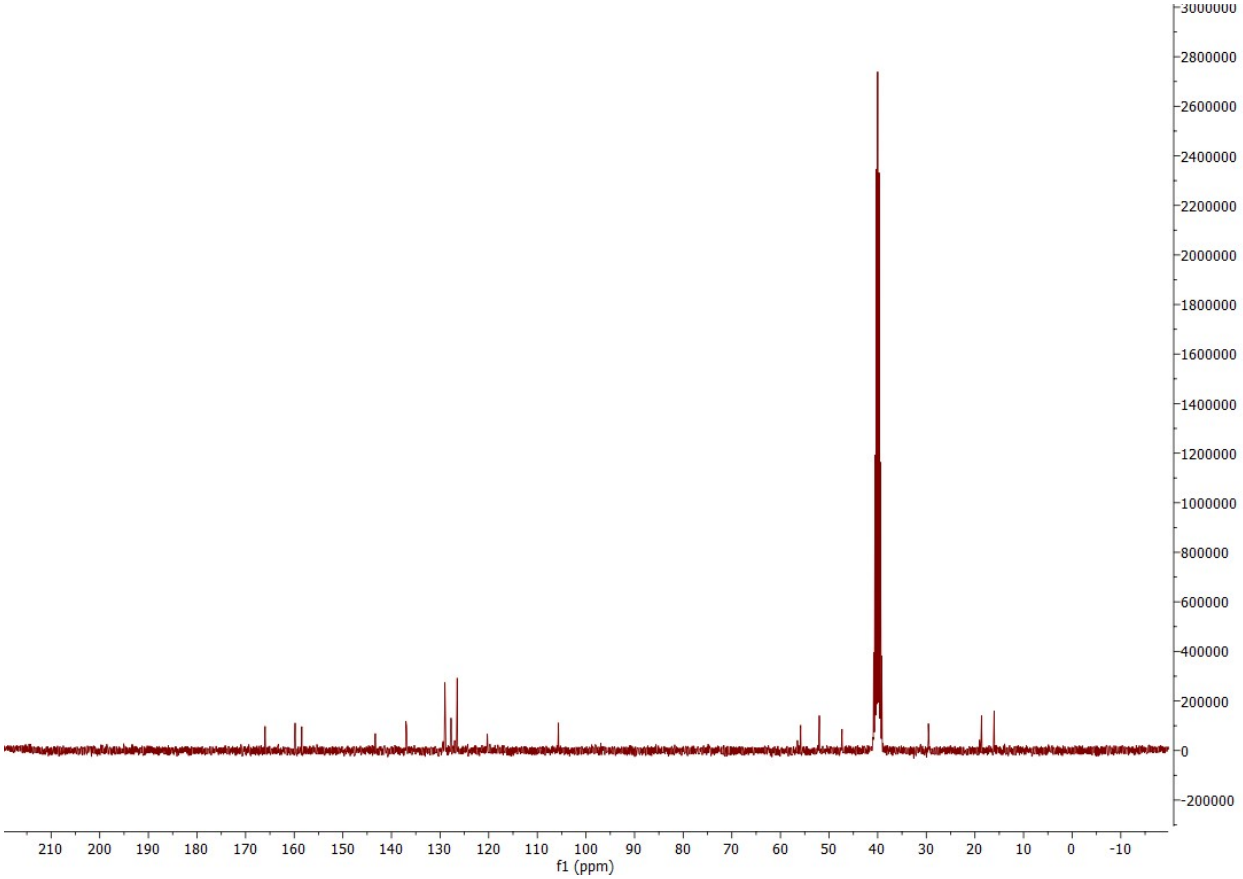

Figure S40: ^{13}CNMR spectrum of derivative **4j**

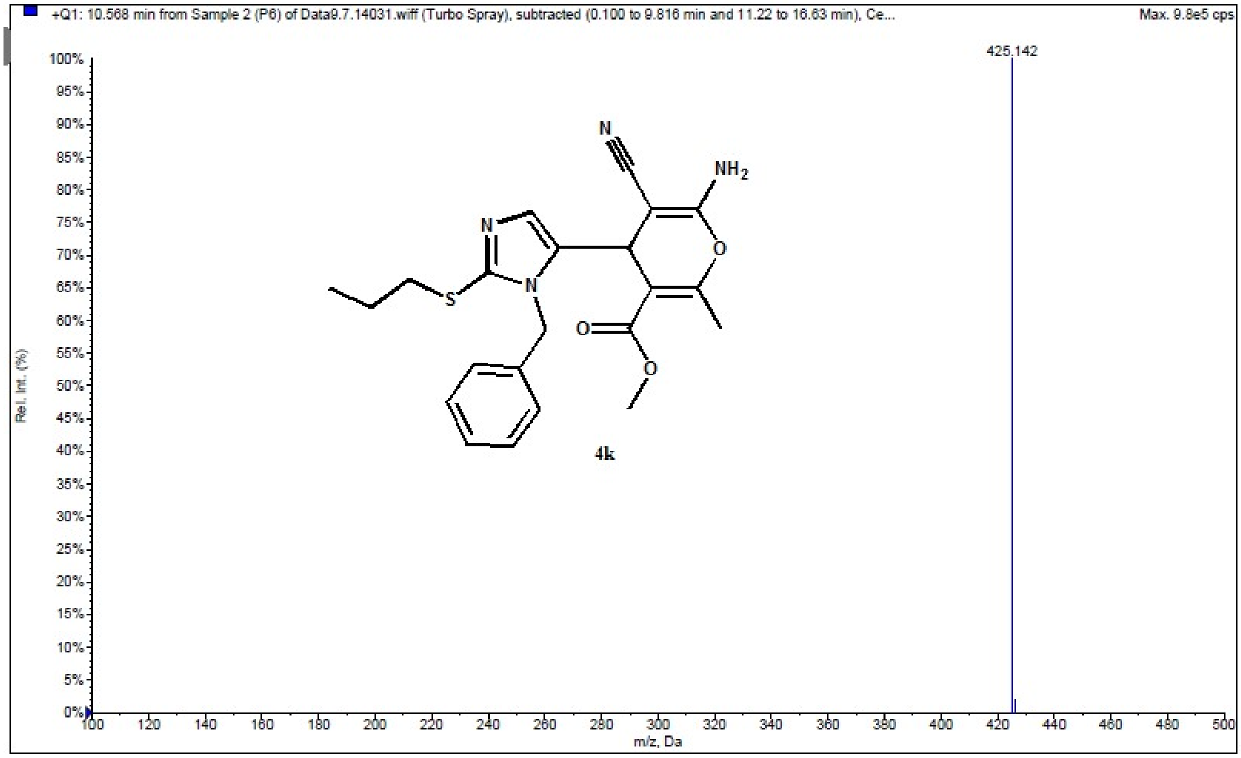

Figure S41: Mass spectrum of derivative **4k**

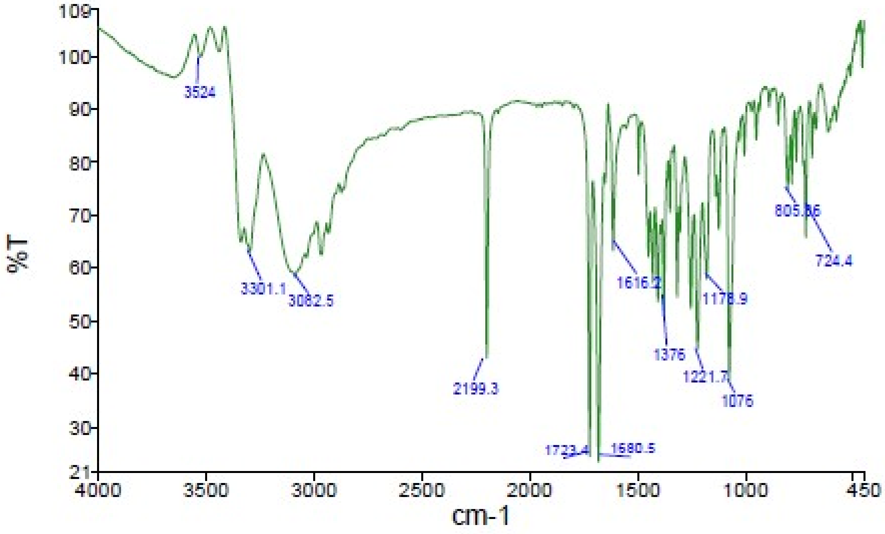

Figure S42: FTIR spectrum of derivative **4k**

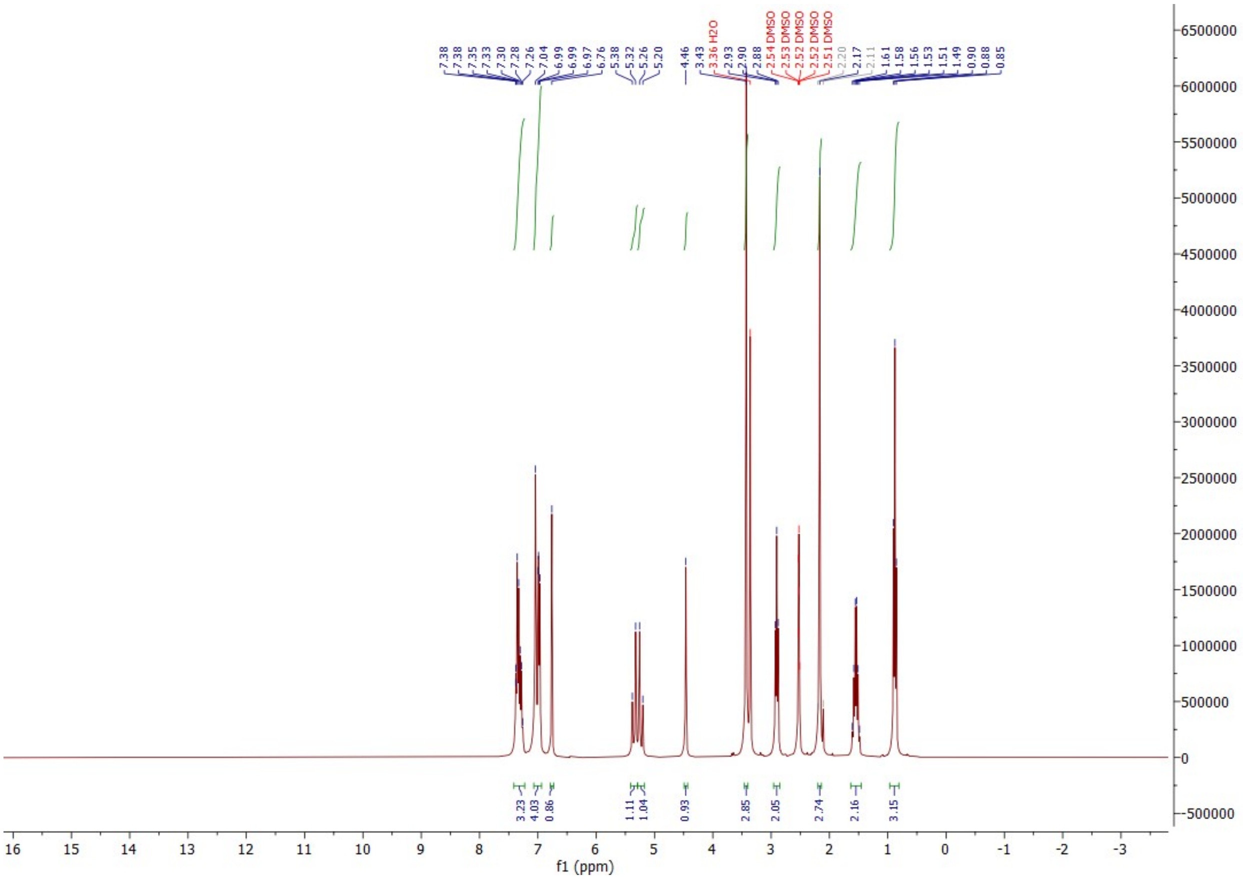

Figure S43: ^{1}HNMR spectrum of derivative **4k**

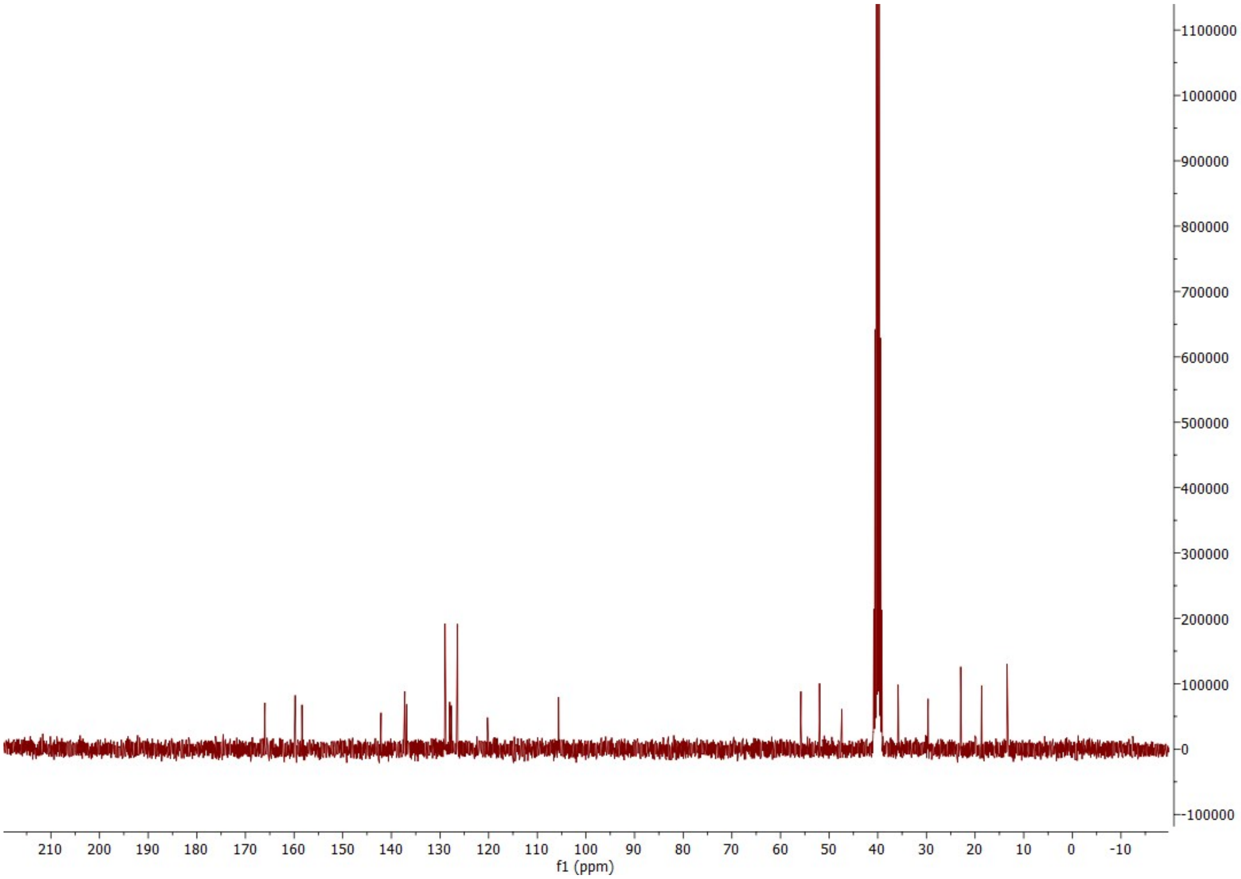

Figure S44: ^{13}CNMR spectrum of derivative **4k**

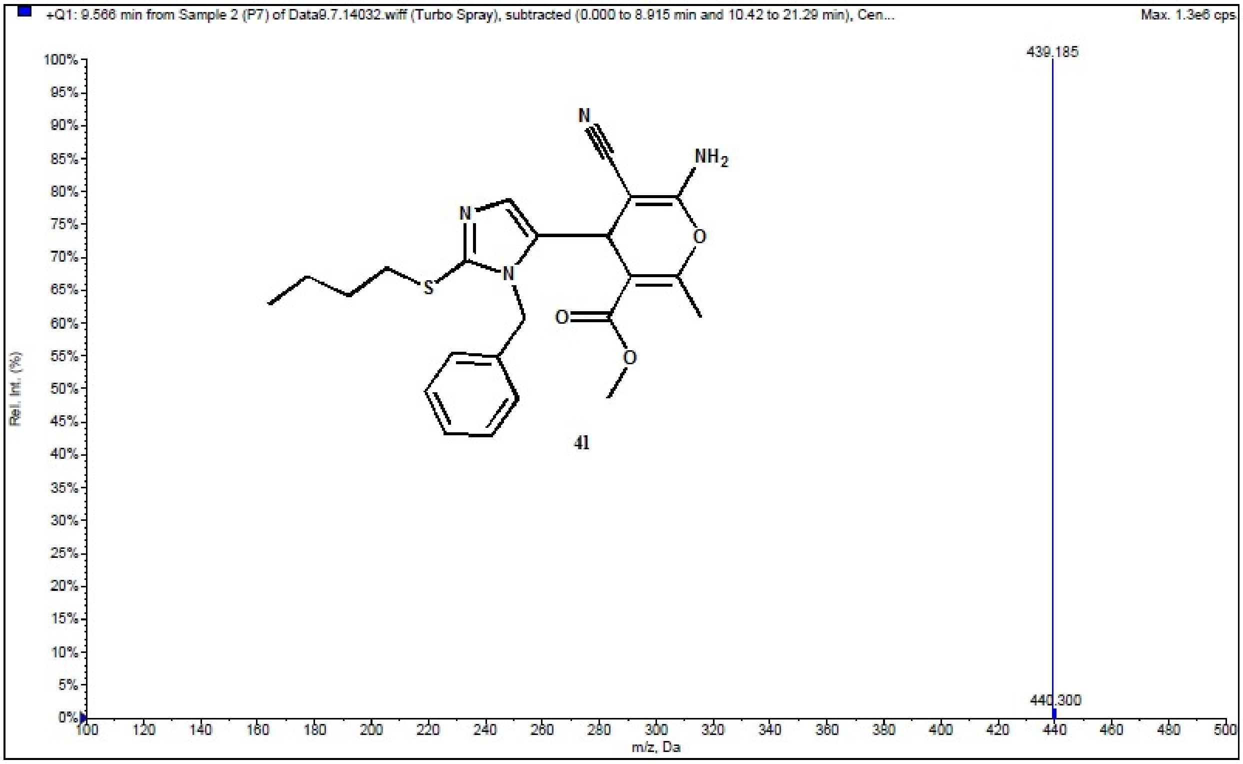

Figure S45: Mass spectrum of derivative **4l**

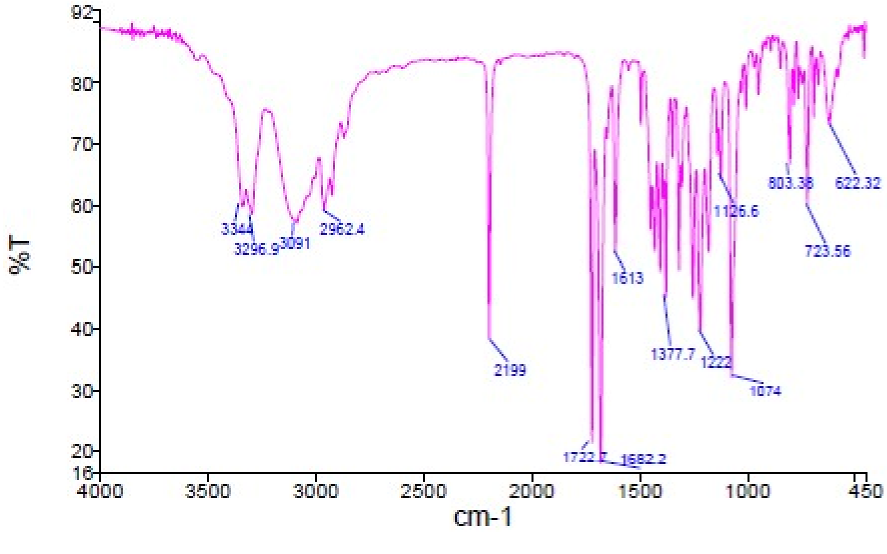

Figure S46: FTIR spectrum of derivative **4l**

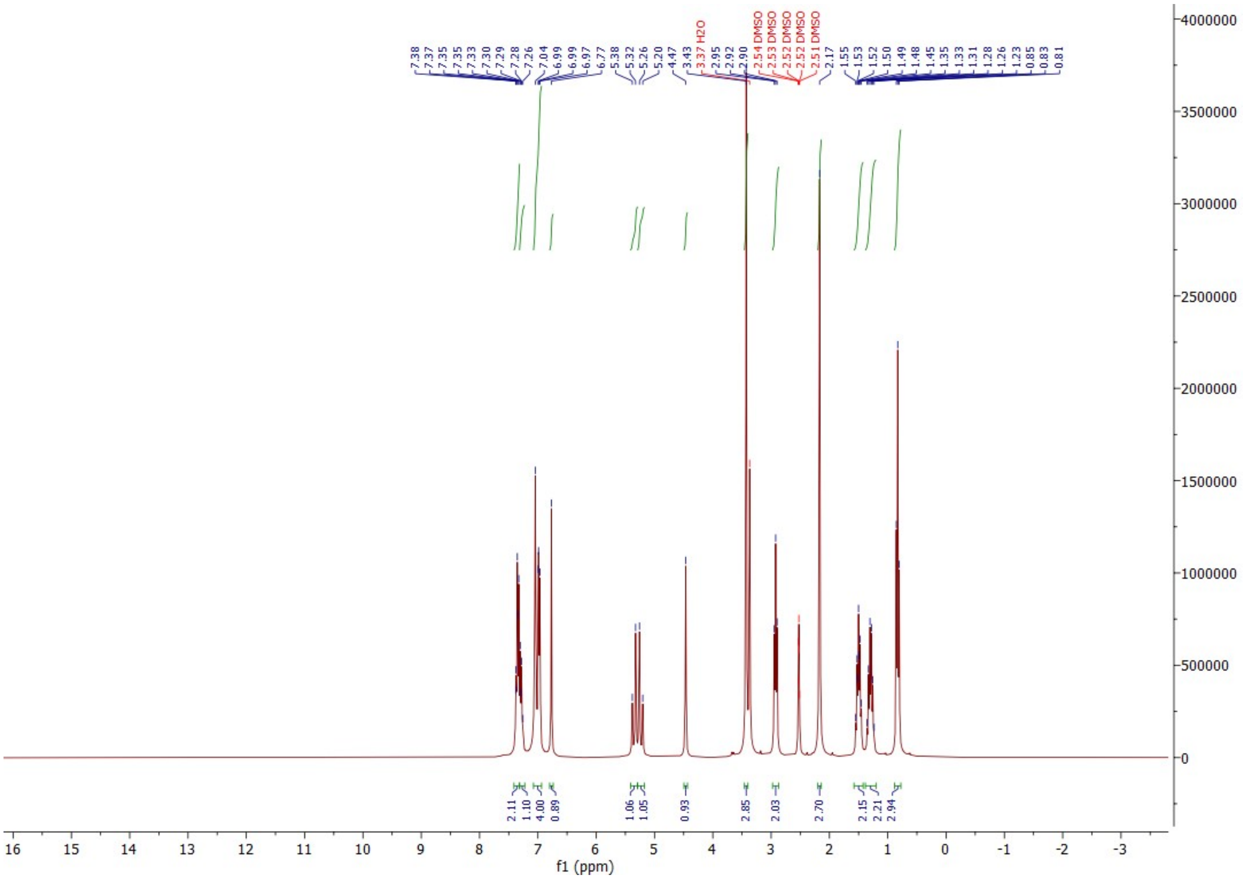

Figure S47: ^{1}HNMR spectrum of derivative **4l**

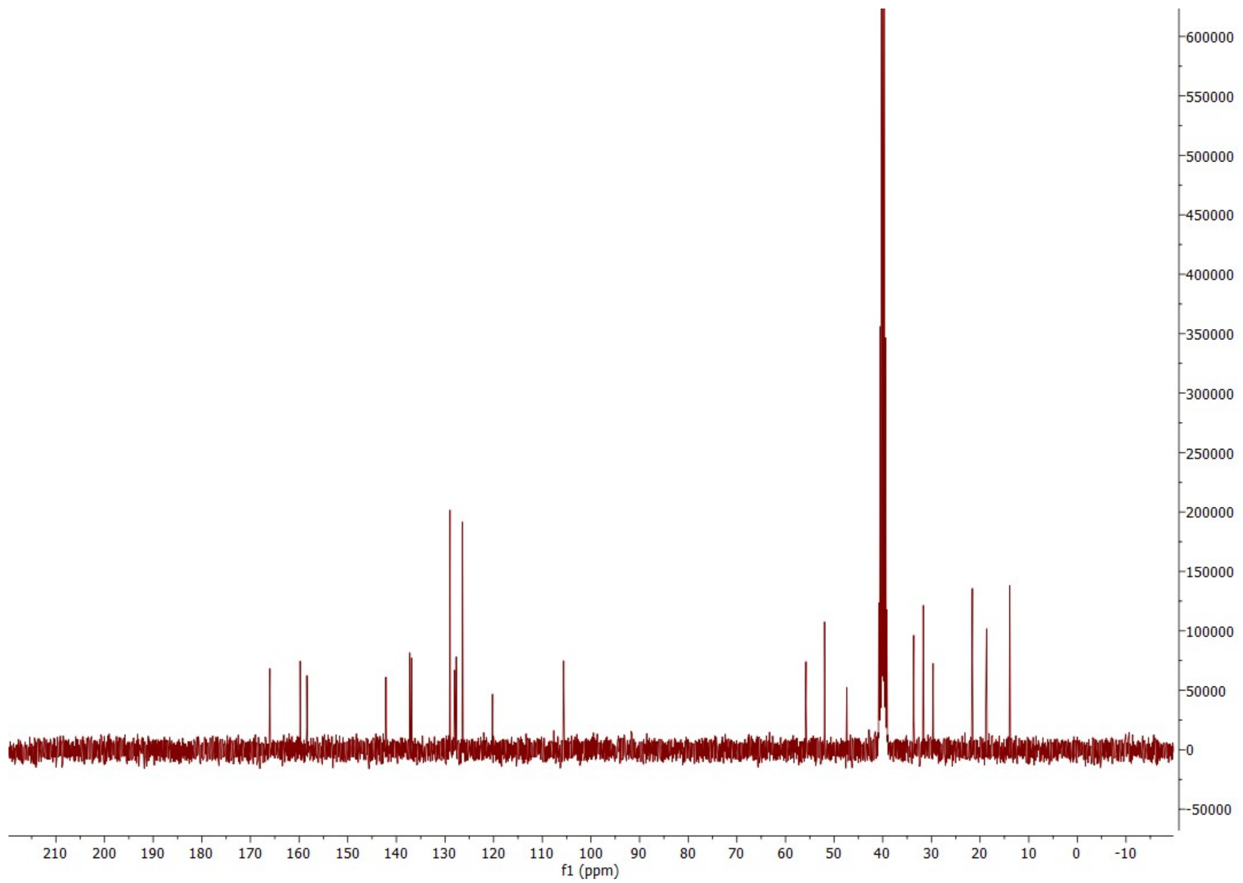

Figure S48: ^{13}CNMR spectrum of derivative **4l**

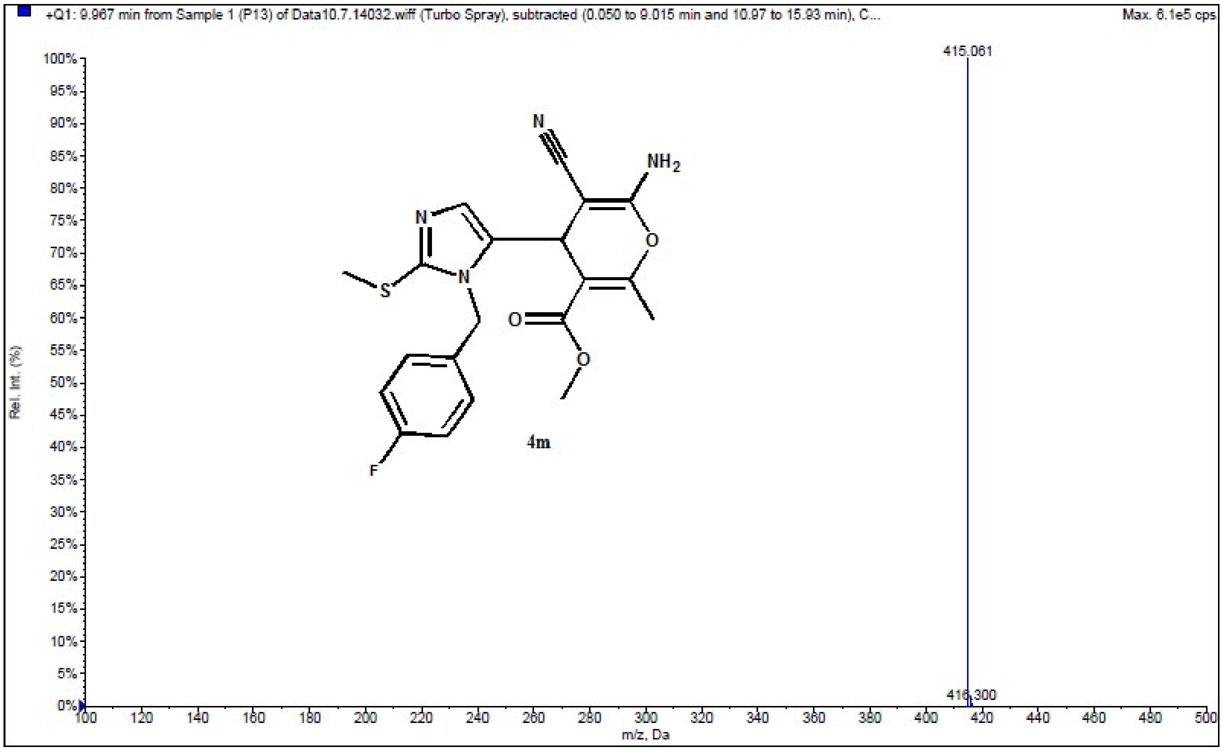

Figure S49: Mass spectrum of derivative **4m**

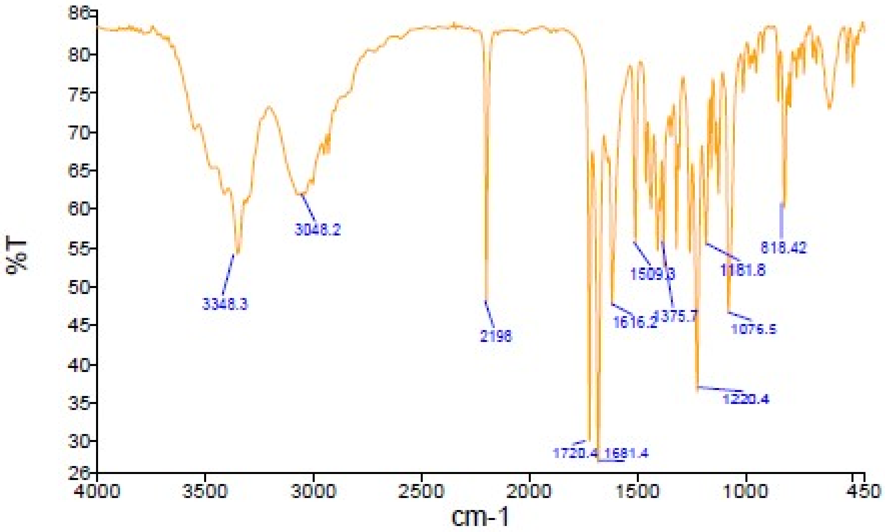

Figure S50: FTIR spectrum of derivative **4m**

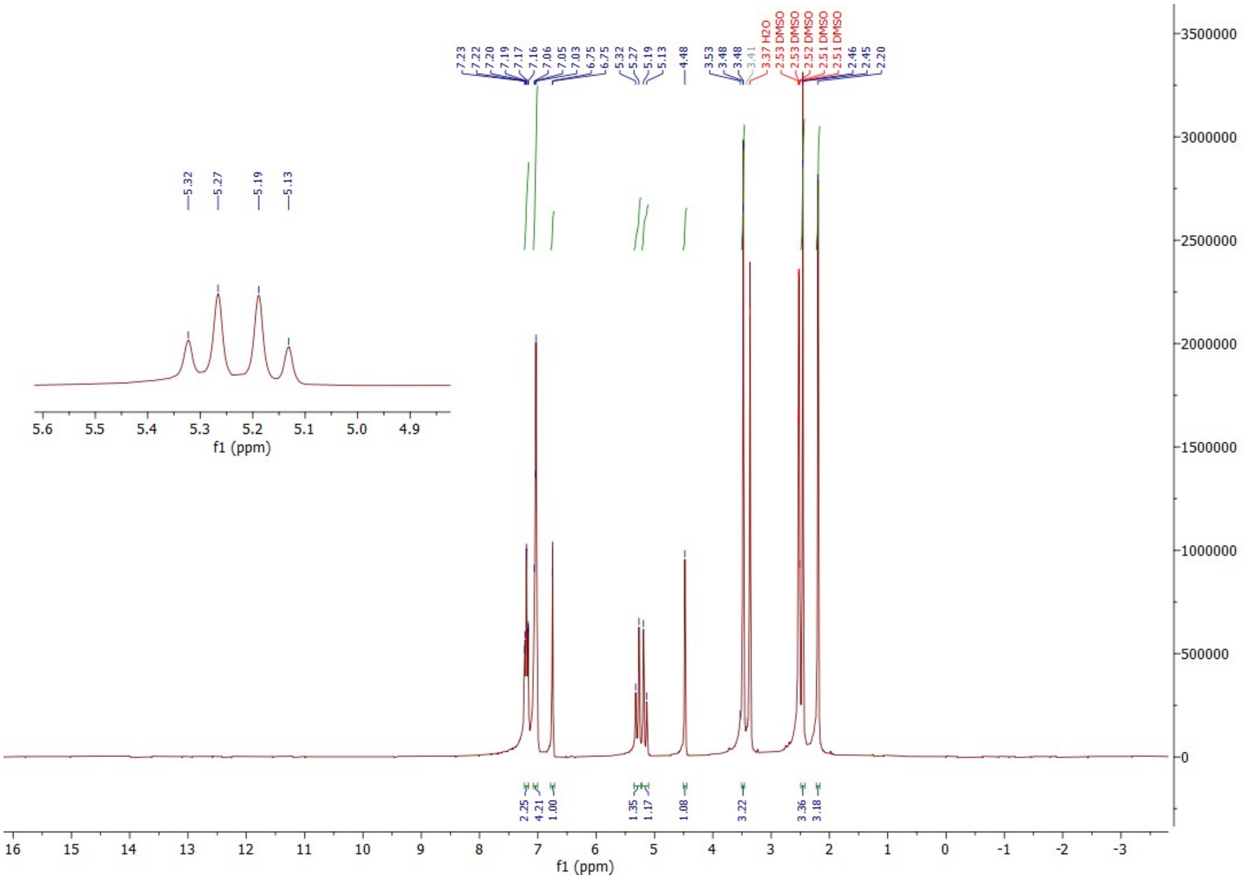

Figure S51: ^{1}HNMR spectrum of derivative **4m**

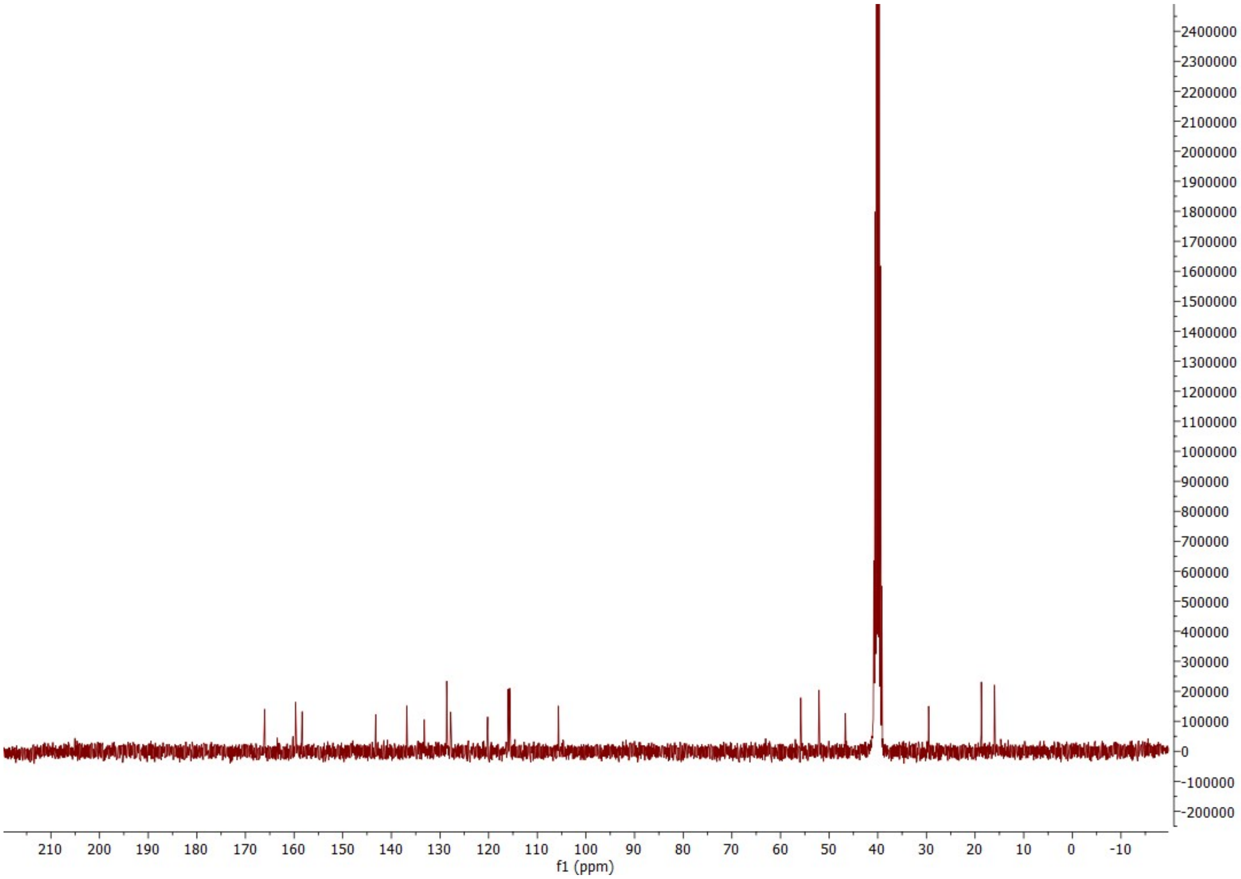

Figure S52: ^{13}CNMR spectrum of derivative **4m**

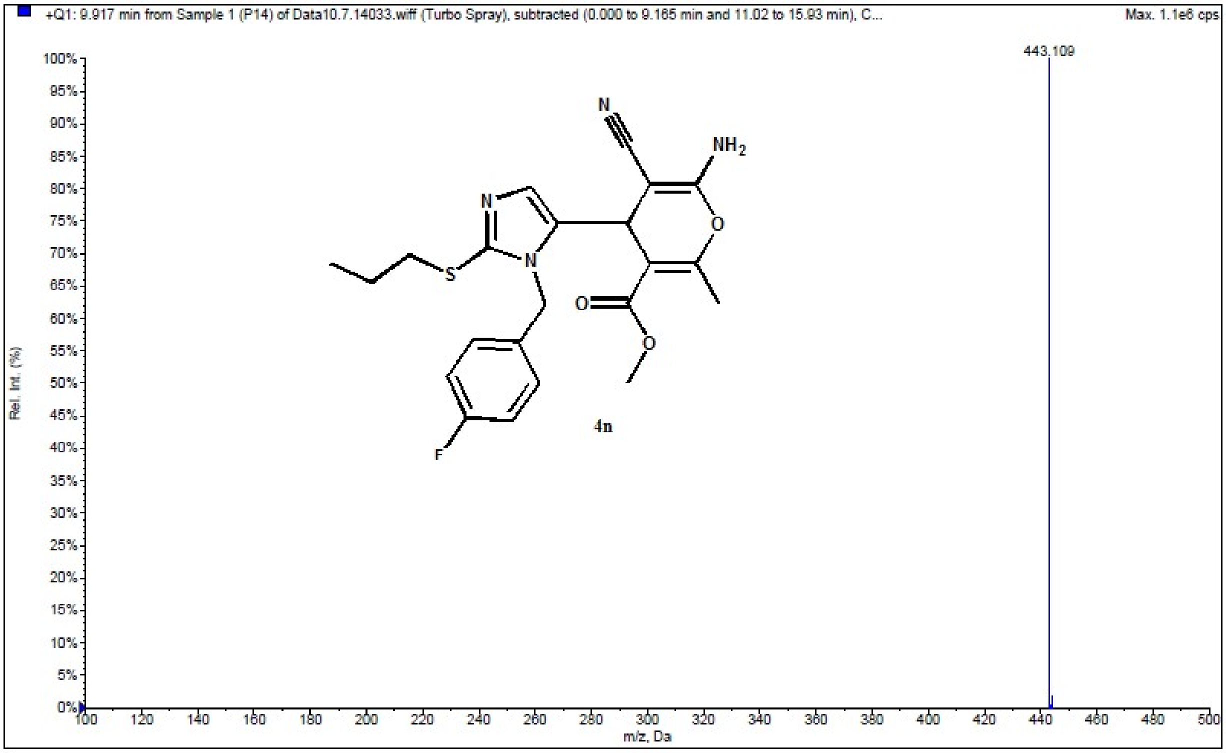

Figure S53: Mass spectrum of derivative **4n**

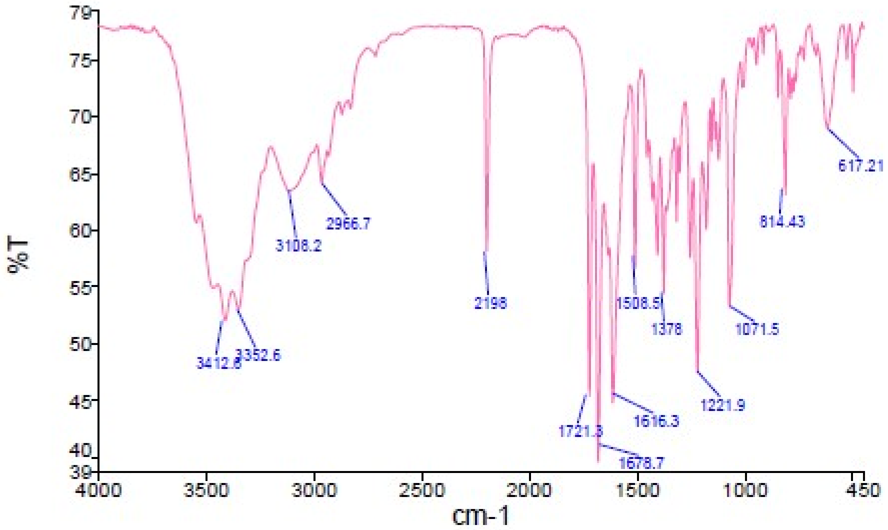

Figure S54: FTIR spectrum of derivative **4n**

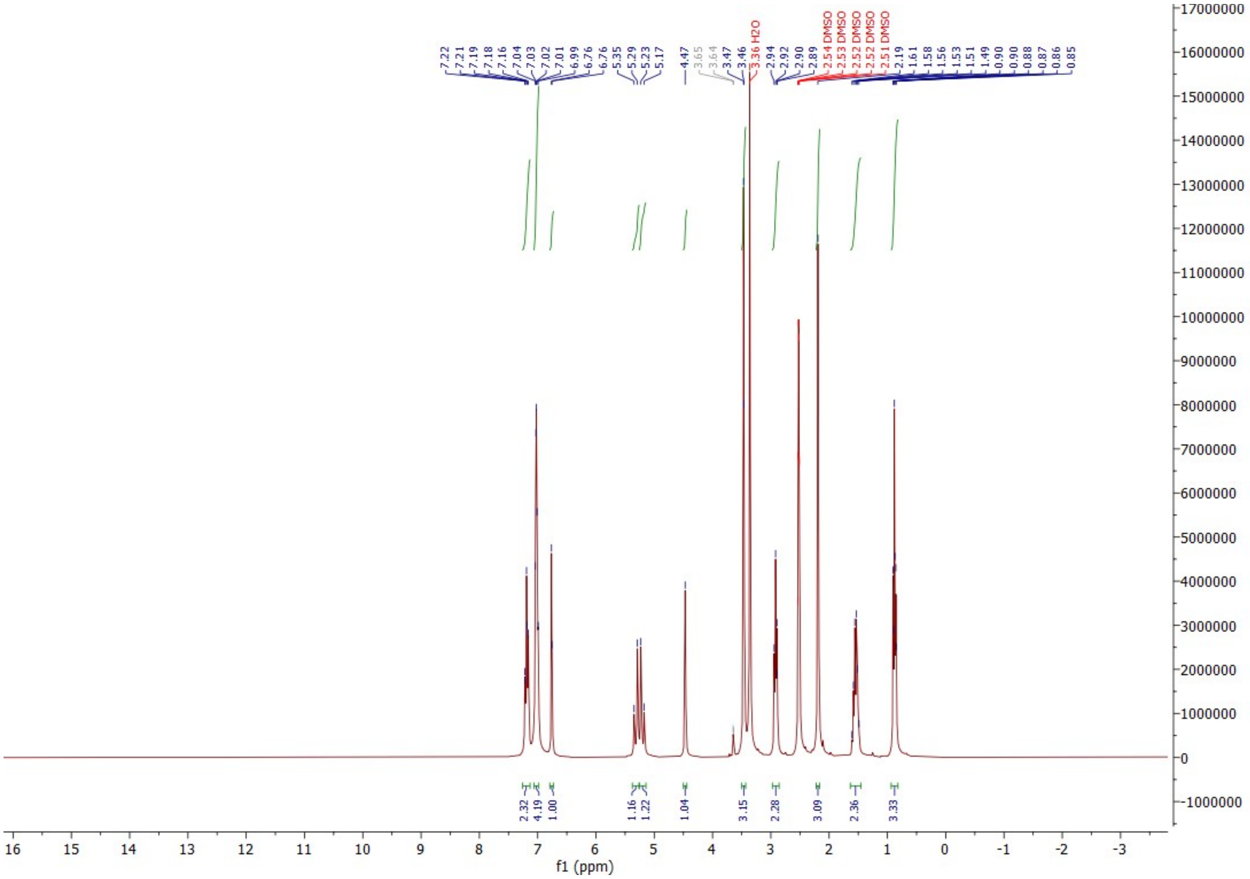

Figure S55: ^{1}HNMR spectrum of derivative **4n**

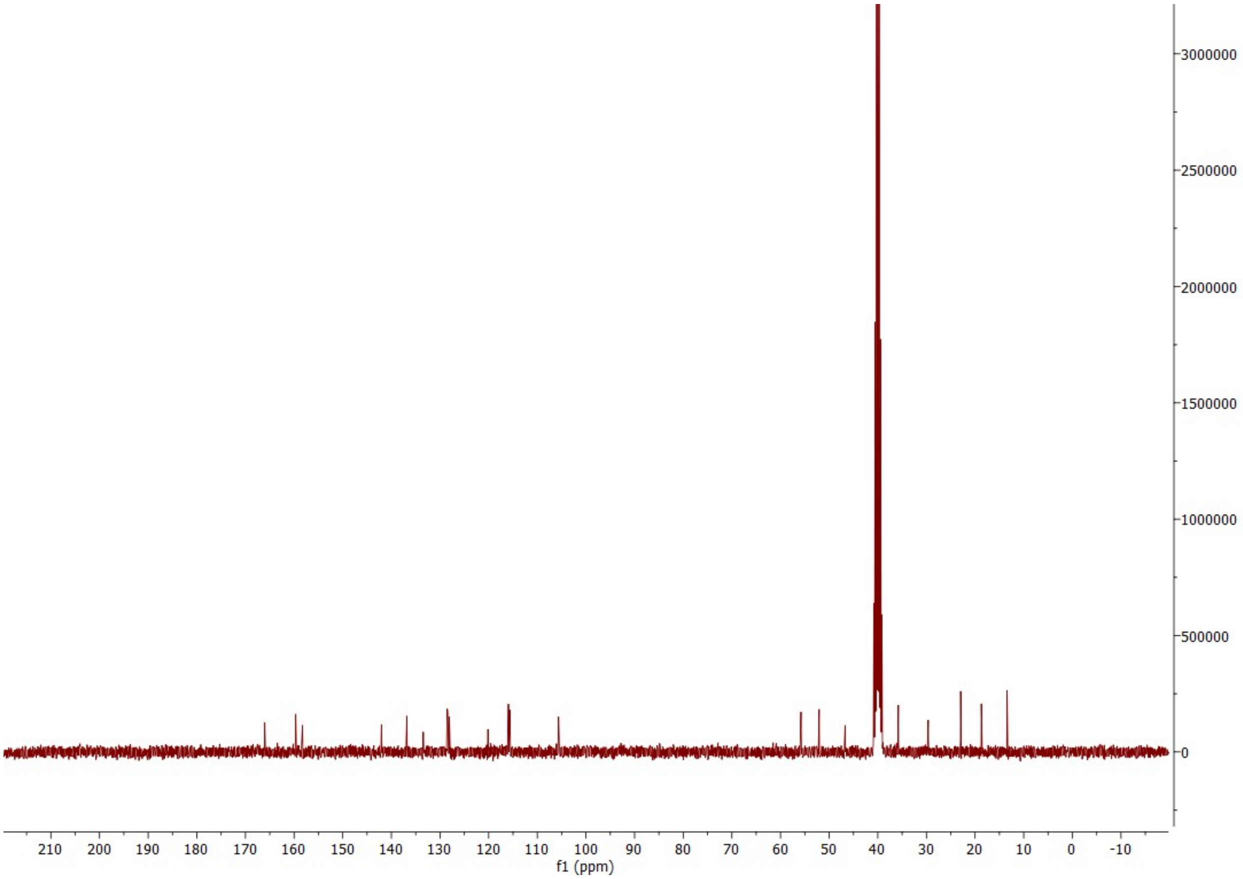

Figure S56: ^{13}CNMR spectrum of derivative **4n**

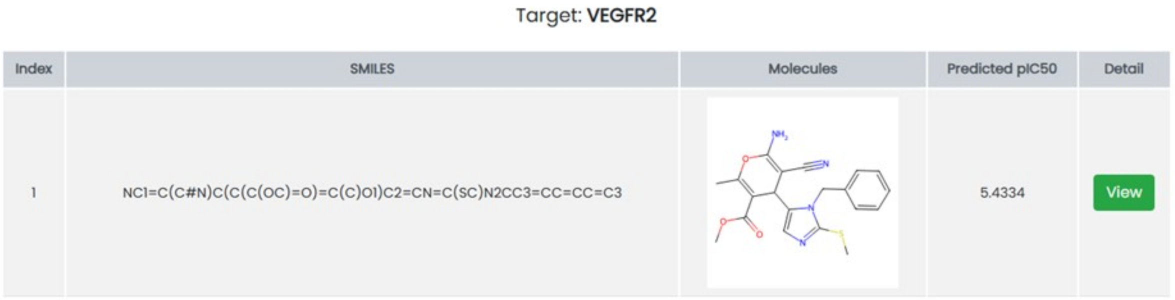
Target: **VEGFR2**

Index	SMILES	Molecules	Predicted pIC50	Detail
1	NC1=C(C#N)C(C(C(OC)=O)=C(C)O1)C2=CN=C(SC)N2CC3=CC=CC=C3		5.4334	View

Figure S57. Bioactivity prediction of **4e** data from (https://fca_icdb.mpu.edu.mo/codd/works/bioactivity_prediction)

Query Structure (SMILES)

O=C(OC)C1=C(C)OC(N)=C(C#N)C1C2=CN=C(SC)N2CC3=CC=CC=C3

Target Structures (One SMILES per line)

O=C(NC)C1=CC=CC=C1SC2=CC=C(C(/C=C/C3=CC=CC=N3)=NN4)C4=C2 Axitinib

Enter one SMILES notation per line for comparison

Similarity Method

Cosine

Fingerprint Type

MACCS Keys

Compare

Similarity Results

Target Structure	Similarity Score	Structure Visualization
O=C(NC)C1=CC=CC=C1SC2=CC=C(C(/C=C/C3=CC=CC=N3)=NN4)C4=C2 Axitinib	0.646	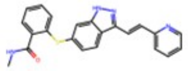

Figure S58. Similarity results between **4e** and **Axitinib** (Xray ligand 4AG8) data from https://chemtoolshub.com/en/.

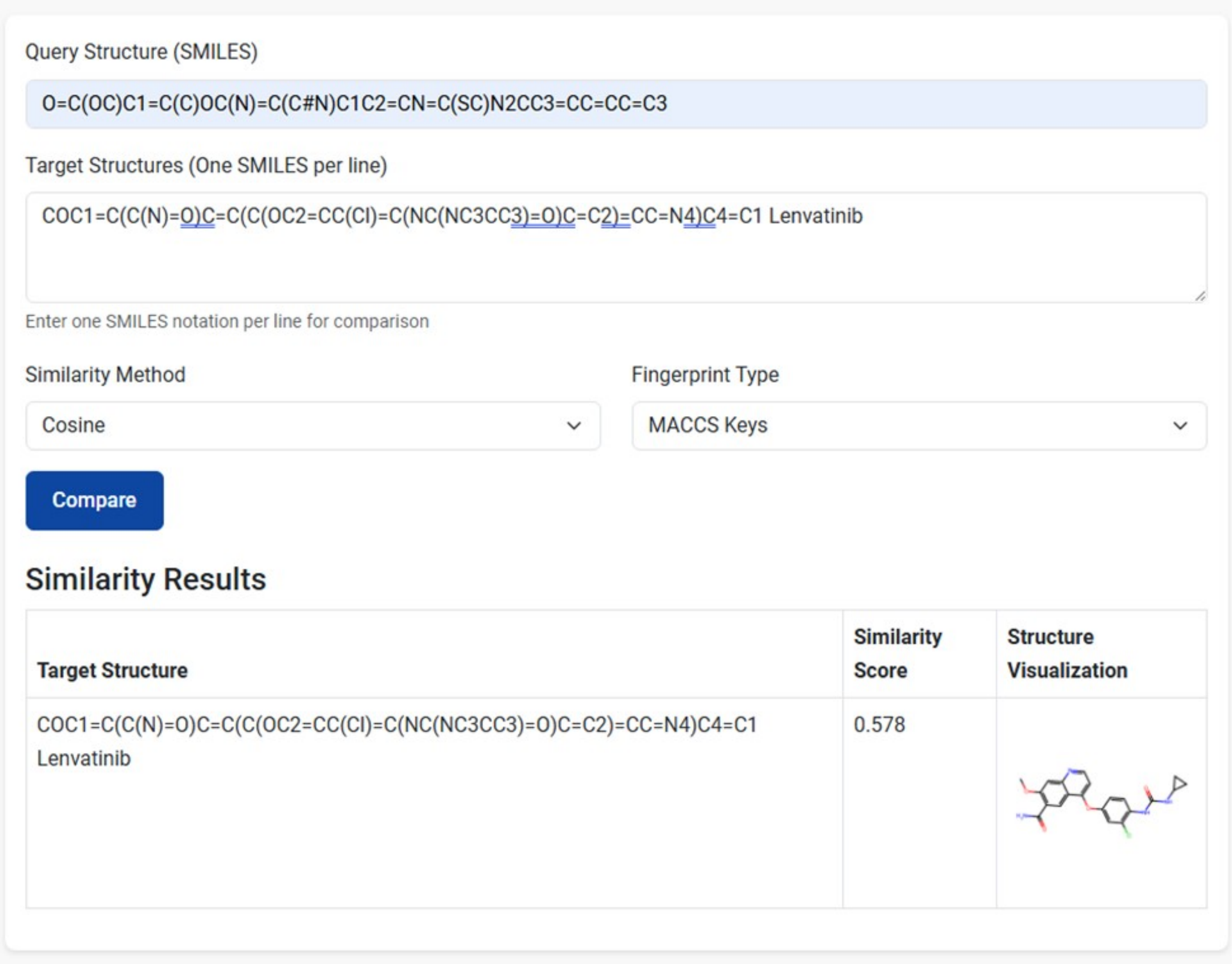

Figure S59. Similarity results between 4e and Lenvatinib (Xray ligand 3WZE) data from https://chemtoolshub.com/en/.

Query Structure (SMILES)

O=C(OC)C1=C(C)OC(N)=C(C#N)C1C2=CN=C(SC)N2CC3=CC=CC=C3

Target Structures (One SMILES per line)

ClC1=CC(OC2=CC=NC3=CC(OC)=C(OC)C=C32)=CC=C1NC(/N=C4NOC(C)=C\4)=O Tivozanib

Enter one SMILES notation per line for comparison

Similarity Method

Cosine

Fingerprint Type

MACCS Keys

Compare

Similarity Results

Target Structure	Similarity Score	Structure Visualization
ClC1=CC(OC2=CC=NC3=CC(OC)=C(OC)C=C32)=CC=C1NC(/N=C4NOC(C)=C\4)=O Tivozanib	0.608	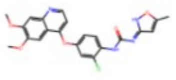

Figure S60. Similarity results between 4e and Tivozabin (Xray ligand 4ASE) data from https://chemtoolshub.com/en/.

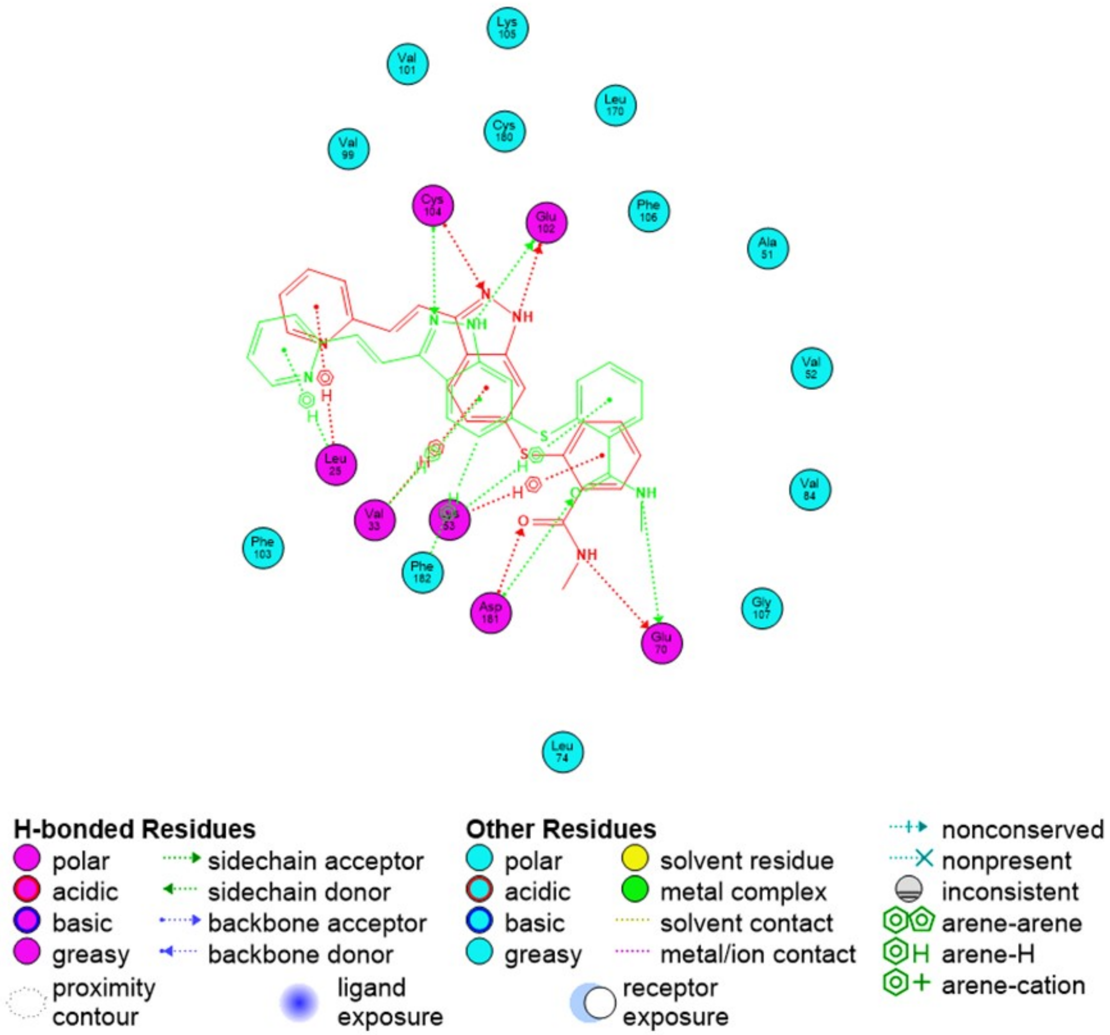

Figure S61. The superimpose structure of X-ray crystal **Axitinib** (green) vs docked **Axitinib** (red).

DockRMSD Results

```
######################################################################
# DockRMSD (v1.1): docking pose distance calculation                 #
#                                                                    #
# If you use DockRMSD in your work, please cite:                     #
#                                                                    #
# Bell, E.W., Zhang, Y. DockRMSD: an open-source tool for atom       #
# mapping and RMSD calculation of symmetric molecules through graph  #
# isomorphism. Journal of Cheminformatics, 11:40 (2019).             #
######################################################################

Calculated Docking RMSD: 0.430

Total # of Possible Mappings: 432
Optimal mapping (First file -> Second file, * indicates correspondence is not one-to-one):
O  1 -> O  1
C  2 -> C  2
N  3 -> N  3
C  4 -> C  5 *
C  5 -> C  9 *
C  6 -> C 10 *
C  7 -> C 12 *
C  8 -> C 14 *
C  9 -> C 16 *
C 10 -> C 17 *
S 11 -> S 19 *
C 12 -> C 20 *
C 13 -> C 21 *
C 14 -> C 23 *
C 15 -> C 25 *
C 16 -> C 27 *
N 17 -> N 28 *
N 18 -> N 30 *
C 19 -> C 31 *
C 20 -> C 32 *
C 21 -> C 33 *
C 22 -> C 35 *
C 23 -> C 37 *
N 24 -> N 38 *
C 25 -> C 39 *
C 26 -> C 41 *
C 27 -> C 43 *
C 28 -> C 45 *
```

Figure S62. RMSD of redock **Axitinib** data from https://zhanggroup.org/DockRMSD

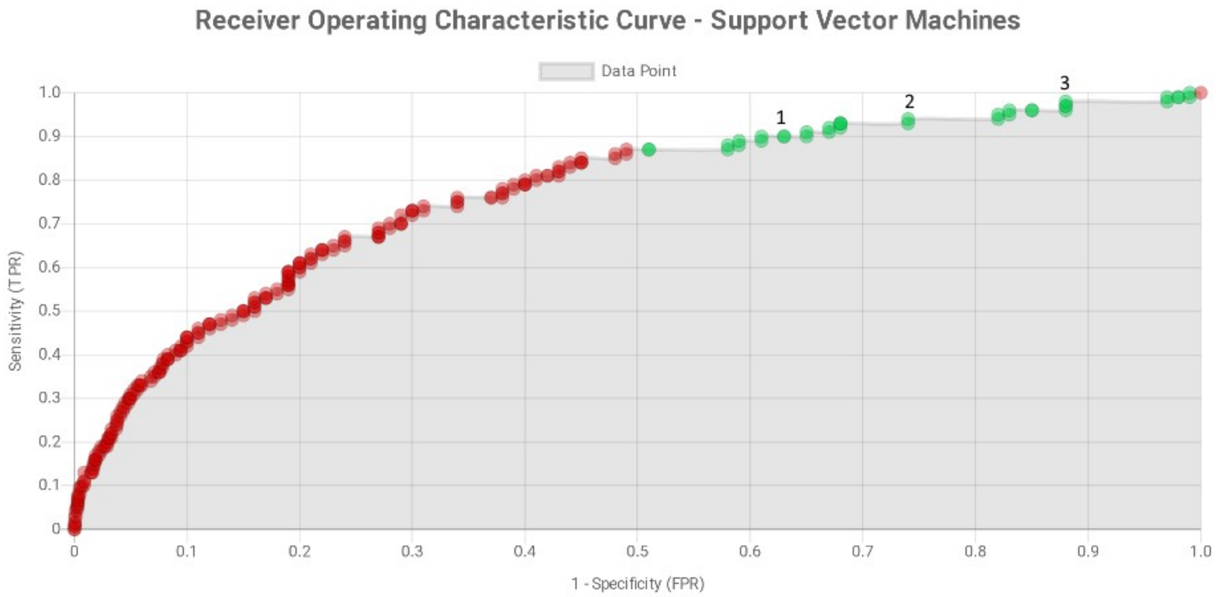

Figure S63. ROC curve for SVM model of **4e** bonded to VEGFR2

Compound	Molecular mass (≤ 500 g/mol)	Hydrogen bond donor (≤ 5)	Hydrogen bond acceptors(≤ 10)	LogP(≤ 5)	Molar Refractivity (40-130)	PASS
**4a**	320	2	6	1.396680	80.357384	⚐
**4b**	334	2	6	1.786780	84.974380	⚐
**4c**	348	2	6	2.176880	89.591377	⚐
**4d**	362	2	6	2.566980	94.208374	⚐
**4e**	396	2	6	2.967080	104.579384	⚐
**4f**	410	2	6	3.275499	109.316383	⚐
**4g**	414	2	6	3.106179	104.537392	⚐
**4h**	410	2	6	3.275499	109.316383	⚐
**4i**	378	2	8	0.939880	91.316383	⚐
**4j**	396	2	6	1.464440	101.665894	⚐
**4k**	424	2	6	2.244640	110.899887	⚐
**4l**	438	2	6	2.634740	115.516884	⚐
**4m**	414	2	6	1.761640	101.716896	⚐
**4n**	442	2	6	2.541840	110.950890	⚐

1S Table. Lipinski's rule of five properties predictions of title compounds

Table S2. The docking scores (kcal/mol) for compounds **4e** and **4h** bond to VEGFR2

Compound	VEGFR2 PDB IDs		
	**4ASE**	**3WZD**	**4AG8**
**4e**	-7.09	-6.23	-8.29
**4h**	-7.46	-6.30	-8.20

Table S3. Similarity between **4e** and Xray ligands of different VEGFR2 proteins

	**VEGFR2 PDB IDs**		
	**4ASE**<br>**Resolution: 1.83 Å**	**3WZD**<br>**Resolution: 1.57 Å**	**4AG8**<br>**Resolution: 1.95 Å**
Xray ligand<br>(**4e** similarity)	Tivozanib<br>(0.608)	Lenvatinib<br>(0.578)	Axitinib<br>(0.646)

Table S4. RMSD for redocking x-ray ligands on11 different PDB IDs for VEGFR2

	1Y6B	2OH4	3C7Q	3CJF	3CJG	3VHE	3VNT	3VO3	5EW3	6GQO	6GQQ
RMSD (Å)	2.01	0.29	2.13	2.33	1.54	1.83	1.01	0.43	0.40	0.41	0.70

Table S5. specificity and sensitivity three points in ROC curve

**Point no**	**Specificity**	**Sensitivity**	**Prediction**
1	0.63	0.90	Active
2	0.74	0.94	Active
3	0.88	0.96	Active
